# Multi-Source Error Compensation for Weighing Rain Gauge Based on Adaptive GOOSE-BP Network

**DOI:** 10.3390/s26144654

**Published:** 2026-07-22

**Authors:** Chenyang Huang, Aiping Xiao

**Affiliations:** Intelligent Equipment Laboratory, College of Engineering, Beijing Forestry University, 35 Qinghua East Road, Haidian District, Beijing 100083, China

**Keywords:** rain gauge, error analysis, adaptive goose optimization algorithm

## Abstract

**Highlights:**

**What are the main findings?**

- The nonlinear error modeling framework is established by integrating multiple disturbance factors, including creep, vibration, and temperature, enabling a dynamic compensation mechanism that adapts to complex environmental conditions.
- An improved adaptive GOOSE (ADGOOSE) algorithm is applied to optimize the BP neural network for error compensation, which significantly enhances model convergence and stability.
- The proposed ADGOOSE-BP method reduces the root mean square error (RMSE) to 0.0494 and increases the coefficient of determination (R^2^) to 0.9835, substantially outperforming conventional filtering and other optimization approaches.

**What are the implications of the main findings?**

- The framework offers a robust, data-driven software solution for multi-source error compensation in weighing rain gauges, improving measurement accuracy without requiring hardware modifications.
- The methodology is extensible to other sensor error compensation tasks where high precision is required under complex, time-varying environmental disturbances, and it provides a foundation for future field deployment and adaptive recalibration.

**Abstract:**

Purpose: To address the measurement inaccuracies of weighing-type rain gauges caused by environmental disturbances such as vibration, temperature drift, and creep, this study aims to develop a robust error modeling and compensation framework adaptable to complex conditions. Method: A nonlinear error model was constructed by analyzing multi-source disturbance factors and incorporating both linear and nonlinear temperature terms. A BP neural network was employed to compensate for complex error patterns, and several intelligent optimization algorithms (a genetic Algorithm (GA), a particle swarm algorithm (PSO), and a GOOSE algorithm (GOOSE)) were used to enhance training performance. An improved adaptive GOOSE algorithm (ADGOOSE) was further proposed to optimize the BP network by integrating dynamic control coefficients and perturbation-based restart strategies. Results: Experiments under various rainfall intensities and temperatures demonstrated that the ADGOOSE-BP model outperformed traditional filtering and other optimization methods, achieving the lowest RMSE of 0.0494 and the highest R2 of 0.9835. Conclusion: The proposed method effectively models and compensates for environmentally induced errors in weighing rain gauges, demonstrating strong potential as a high-precision, adaptive compensation framework that provides a solid foundation for future field-deployable hydrological monitoring systems.

## 1. Introduction

Accurate measurement of precipitation is important in meteorology and hydrology [1]; accurate recording of precipitation intensity is a prominent issue even in the current state of the art; at the same time, the practical role of accurate recording of precipitation intensity has been greatly underestimated [2]. The great uncertainty in the time and space of rainfall occurrence and the complexity of field environments make it difficult for existing techniques to meet the requirements of accurate rainfall monitoring in different contexts [3,4,5].

The measurement accuracy of existing rain gauges is easily affected by low rainfall power, small measurement ranges, and large measurement errors for heavy precipitation. Now, in the development of rain gauges, improvements in measurement accuracy and upgrading are required to make them more intelligent [6]. In recent years, the Pluvio weighing rain gauge, which has a high measurement accuracy and low power consumption, has been gradually applied in rainfall monitoring [7,8].

Based on the principle of the weighing method, Zhan Xiaoyun et al. designed a weighing rain gauge that integrates the functions of sample collection and measurement, data transmission and calculation, remote control and diagnosis, etc. The instrument takes an STM32 microcontroller as the core and utilizes an A/D converter chip to amplify and process the voltage signal of the weighing sensor to obtain rainfall data with a resolution of 0.01 mm at the minute level. The test results show that the standard deviation of the rain gauge measurement is 0.02 mm/min, and the measurement accuracy reaches up to 98.67%. The weighing rain gauge has a high resolution and sensitive response to tiny raindrops, such that it can accurately monitor the whole process of rainfall in real time and provide a reference for improvement in the precision and automation level of rainfall monitoring technology [9].

Wang et al. developed a load-cell-based siphon-drainage self-compensating rainfall measurement device to overcome the errors of traditional siphon rain gauges that limit automated field applications. The load cell converts rainfall weight into an electrical signal, which is then differentially amplified, digitized, and processed to obtain rainfall data. Based on experimental siphon data, they established a high-precision compensation model that eliminates siphon-induced errors, reducing the overall measurement error to within 1%. The system also supports online real-time monitoring and features convenient power supply, stable communication, and high accuracy [10].

Xu Kewen et al. used the precipitation data of an automatic rain gauge and a siphon rain gauge in the Shanxi Observatory from 2004 to 2011 to compare and analyze self-recorded records of precipitation to find out the reasons for and the laws governing the errors generated by the measurement characteristics of the two instruments and to summarize the possible error range caused by precipitation of different intensities, as well as the characteristics of the error distribution of different intensities of precipitation, so as to provide a scientific basis for the measurement and reporting of precipitation quality control. To this end, a revised formula and an error range for precipitation of different intensities was derived; the error range of the rain gauge at the existing automatic station was provided in the data for short-term heavy precipitation, and the characteristics of precipitation recorded by different rain gauges were analyzed. The reasons for the error of the instrument and the advantages and disadvantages of the collection were also analyzed [11].

A resistive strain load cell has been widely used in the measurement of mass or force under static and dynamic conditions due to its simple fabrication, mature technology and high accuracy; it has been applied in a large number of applications in the fields of industrial production process detection, control and automatic weighing [12].

Wang Yougui et al. reported that the resistance strain load cell exhibits a serious creep error that directly affects the accuracy of weighing results and put forward a neural network based on a load-cell creep error automatic compensation model and gave the training algorithm of the model [13].

Building on this approach, Cheng et al. proposed an error-suppression algorithm for shipboard load cells based on a period-sliding-average Kalman filter. In their method, the raw data are first processed by a conventional Kalman filter to remove random noise. The filtered data are then subjected to the short-time Fourier transform (STFT) to extract the frequency characteristics of the periodic errors. Finally, a sliding window mean filter is applied to eliminate the residual periodic errors from the system [14].

Dutton, M et al. designed a simple-to-use intelligent instrument to minimize and possibly eliminate under-catch measurement errors balancing out the water budget. The best aerodynamic shape for a precipitation gauge was determined to minimize out-splash and maximize catch. Comparison field work1 and Computational Fluid Dynamic4 (CFD) research was undertaken on standard straight-sided, ‘chimney’-shaped, aerodynamically shaped and pit-installed (out of the wind) gauges. These new instruments use ultrasonic wind sensors and Doppler shift measuring techniques to obtain wind-versus-rainfall catch data. Also, using optical and/or impact sensing techniques, we can measure the individual drop size and count the drops involved in a rain event. By adding weighing technology to the tipping bucket design and improving calibration methods, we can improve resolution and detect evaporation losses. Also, power-efficient and controlled heating allows the inclusion of solid precipitation measurements. Then, machine learning (ML) techniques can be used to correct the errors [15].

Xiong, Z. et al. proposed a two-layer genetic algorithm–backpropagation (GA-BP) model. The first layer uses GA-optimized BP for fault recognition, the second for data fusion. Compared to a single BP, it reduces fusion runtime by 2.37 s; improves recognition accuracy for lost signals, high bias, and low bias by 26.09%, 18.18%, and 7.15%; lowers the MSE by 3.49 mm; and produces smoother outputs, enhancing robustness and generalization [16].

Simões, C.F. et al. developed a laboratory method for the traceability of a rainfall weighing gauge, including an evaluation of the measurement uncertainty. The adopted procedure is similar to the one used for non-automatic weighing instruments. A static approach is followed to achieve the calibration deviation of the precipitation scale. The method was used to evaluate measurement uncertainty based on a nonlinear mathematical model. The Monte Carlo method was used to calculate uncertainties and validate estimates following the conventional Guide to the Expression of Uncertainty in Measurement (GUM) approach. Measurement uncertainty contributions of input quantities in the mathematical model used to calculate rainfall also require specific calibration procedures. The results show the accuracy level achievable with rainfall weighing gauges commonly used as a reference for meteorological monitoring networks and data modeling [17].

In order to carry out rapid, continuous and accurate measurement and to obtain steady states for the values measured, it is required to correctly describe and analyze the weighing force transducer, signal conditioning, processing, displaying, recording, and resulting dynamic weighing force measurement system, and it is also required to reduce the uncertainty of the dynamic measurement to improve the dynamic response speed and to decouple the multi-component components in order to carry out the dynamic compensation [18]. The main sources of error in weighing rain gauges are creep error [19], temperature error [20], vibration disturbance error [21,22], and random disturbance error [23]. For this reason, this paper will take the load cell used to measure and record precipitation as the object and carry out its error analysis and fitting compensation when recording precipitation.

Simões et al. proposed and validated a laboratory traceability method for weighing rain gauges, including the evaluation of measurement uncertainty. The calibration procedure draws on the verification approach for non-automatic weighing instruments, using standard weights as the calibration reference instead of water volume. This circumvents errors introduced by environmental factors such as temperature and density that are inherent in conventional water-volume-based calibration. Through static calibration under laboratory conditions, the method achieves traceability of weighing rain gauge measurements to a mass reference, thereby providing a traceable technical pathway for rain gauge calibration.

Lanza and Cauteruccio, in their editorial for a special issue of *Water*, pointed out that although precipitation is a crucial environmental variable closely linked to human activities and economic production, current operational precipitation measurements neither achieve the accuracy required for high-precision applications nor possess a rigorous standardization framework [24]. Different countries adopt varying approaches to measurement accuracy and uncertainty handling in precipitation records, which severely limits the comparability and usability of measurement data on a global scale. The editorial summarizes the main sources of error in precipitation measurement instruments (covering both liquid rain and solid snow) and their impacts on scientific research and applications. It also introduces the first international standard for precipitation gauge accuracy issued in Europe (EN 17277:2019) [25], while noting that standard-setting at the global level (ISO and WMO) still lags relatively behind.

## 2. Weighing Rain Gauge Error Analysis and Compensation

### 2.1. Structural Principle of Weighing Rain Gauge

**Figure 1:**
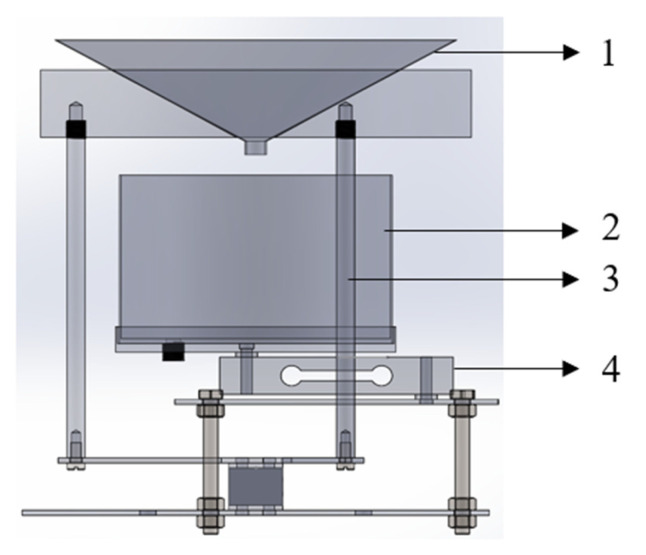
Internal structure of rainfall collection measurements for weighing rain gauges (1—collection funnel, 2—measuring bucket, 3—support rod, 4—load cell).

The overall mechanical structure of the system consists of three main parts: the anti-foreign-body mechanism, the housing structure and the internal precipitation measurement unit. The anti-foreign-object mechanism adopts a stepping motor-driven rotary structure to realize the active covering and opening of the measuring area of the rain gauge. The housing is made of corrosion-resistant alloy material with high sealing performance, which can effectively prevent rainwater infiltration and dust interference. The internal precipitation measurement part adopts the weighing measurement module, whose structure is integrated with the shell to ensure the rigidity and stability of the measurement platform and reduce the error propagation caused by mechanical interference. Figure 1 shows the internal structure of rainfall collection measurements for weighing rain gauges.

### 2.2. Measuring Principle

Rainfall enters the rain gauge through the collection opening and passes into the funnel; the funnel outlet then directs the water into the measuring bucket. The combined weight of the funnel and bucket causes a change in the load-cell voltage, which is transmitted to the control board. The control board filters and processes the received signal, converts the analog signal into digital form, and finally sends the digitized data to the host computer. The rainfall calculation formula is as follows:

(1) $$h=\frac{\left(G_{h}-G_{0}\right)}{\pi r^{2}\rho }\times 10$$

where *h* is the rainfall in mm, $G_{h}$ is the current mass in g, $G_{0}$ is the initial mass in g, r is the radius of the collection bucket in cm, ρ is the density of water in g/cm, and 10 is the conversion factor.

### 2.3. Sources of Error

The main sources of error in the measurement process of weighing rain gauges are creep error, vibration interference error, temperature error and random interference error.

#### 2.3.1. Creep Error

Creep error is defined as the time-dependent variation in the output signal of a load cell under a constant applied load and stable environmental conditions. Such creep behavior significantly degrades both measurement accuracy and long-term stability, making effective compensation essential for high-precision applications.

The creep of the load cell generally changes according to the exponential law; the strain in the elastomer under external load is

(2) $$\varepsilon =\varepsilon_{lin}+\varepsilon_{vis}-\frac{e^{4}}{t}$$

where $\varepsilon_{lin}$ represents the linear strain of the elastomer, $\varepsilon_{vis}$ represents the viscous–elastic strain of the adhesive layer of the strain adhesive, and e is natural constant.

Strain is linearly related to stress, so the creep error, ε, is simplified as $R_{1}+R_{2}M$. $R_{1}$ is related to specific working conditions, and $R_{2}$ represents the elastic strain factor.

#### 2.3.2. Vibration Errors

Load cells are often adversely affected by low-frequency vibrations that can be caused by the design of the mechanical structure itself as well as by internal or external shocks. Ambient vibration adversely affects the response of the piezometric element, which in turn can deteriorate transducer performance. Overcoming this problem by low-pass filtering is often ineffective; low cut-off frequencies are often used to eliminate the effects of vibration by introducing delays and increasing the time between filtered signals, which is not in line with the current trend of fast weighing.

Let *m*_1_ represent the mass of the weighing bucket, *m*_2_ represent the mass of the base; *k*_1_ and *c*_1_ represent the connection stiffness and damping coefficient of the weighing bucket and the base, *k*_2_ and *c*_2_ represent the connection stiffness and damping coefficient of the base and load cell, *x*_1_ and *x*_2_ represent the displacement of the weighing bucket and the base, *F*_1_ represent the impact force of the bucket on precipitation fall, and v represent the coefficients of the vibration error. According to Newton’s second law, a set of differential equations is obtained as shown below:

(3) $$m_{1}\ddot{x_{1}}+k_{1}\left(x_{1}-x_{2}\right)+c_{1}\left(\dot{x_{1}}-\dot{x_{2}}\right)=0$$

(4) $$m_{2}\ddot{x_{2}}+k_{2}x_{2}+c_{2}\dot{x_{2}}-k_{1}\left(x_{1}-x_{2}\right)-c_{1}\left(\dot{x_{1}}-\dot{x_{2}}\right)=0$$

(5) $$F_{1}=vP$$

#### 2.3.3. Temperature Errors

The Wheatstone bridge in the load cell will generate zero drift after energization, and the zero drift will be amplified with the temperature change, so that its measurement accuracy is seriously deteriorated. Methods for software compensation according to calibration data to build the pressure to be measured and the sensor output of the function for the purpose of temperature compensation can be divided into artificial intelligence methods and numerical calculation methods.

Circuit characteristics are affected by temperature.

(6) $$U_{0}=\frac{R^{2}\left(r_{1}^{\prime }+r_{3}^{\prime }-r_{2}^{\prime }-r_{4}^{\prime }\right)+a_{1}}{4R^{2}+2Rr_{sum}+a_{2}}\cdot U_{i}$$

The sum of various deviations caused by changes in the length of the sensitive grids of the resistive strain gauges, the effect of temperature changes, and inconsistencies in the tuning resistance are denoted by *r*_1_, *r*_2_, *r*_3_, and *r*_4_, respectively.

Strain characteristics are affected by temperature.

(7) $$\varepsilon =\frac{\alpha }{K}∆T+\left(\beta_{m}+\beta_{g}\right)∆T+\varepsilon_{\sigma }$$

Temperature error also includes the thermal hysteresis effect of the sensor. Thermal hysteresis refers to the delayed response of the sensor to temperature changes, which is caused by its thermal inertia (e.g., material heat capacity and thermal resistance). This phenomenon directly affects both temperature measurement accuracy and dynamic response characteristics. In load cells, thermal hysteresis is mainly attributed to the thermal resistance of the encapsulation: the thermal conduction delay through the sensor housing and filling materials causes the internal temperature to lag behind the ambient temperature. The resulting first-order thermal-force coupling model is expressed as

(8) $$∆P_{o}\left(t\right)=K_{P}\;*\;P_{t}\left(t\right)+K_{T}\;*\;(T\left(t\right)+\tau \frac{dT\left(t\right)}{dt})$$

where $∆P_{o}\left(t\right)\;$ is the sensor output pressure error, $K_{P}$ is the pressure sensitivity coefficient, $K_{T}$ is the temperature sensitivity coefficient, τ is the thermal hysteresis time constant (related to the material heat capacity and thermal resistance), and T(t) is the ambient temperature change function.

#### 2.3.4. Step Response Error

The assessment of the step response of the weighing gauges illustrates some of the drawbacks that can affect their suitability for rainfall intensity measurement. There was a definite time delay with respect to the sudden variation in intensity (the step response). For weighing gauges measuring rainfall intensity, the step response error refers to the time required for the output signal to stabilize after a sudden change in rainfall intensity. When the temporal resolution is less than one minute, the step response error increases significantly; conversely, it decreases as the duration of the constant pulse increases. While the step response error is critical for high-frequency rainfall intensity measurements under unsteady-state conditions, we note that the primary focus of our study is total accumulated rainfall depth.

Finally, the response time resolution set in this paper is 1 min.

### 2.4. Construction of Mathematical Model of Error

The main influences on the sources of error are the current collected rainfall, M; the current temperature, T; the change in temperature over time, dT; the rainfall intensity, P; and the change in rainfall intensity over time, dP.

These environmental variables cause significant perturbations to the sensor output data in the rainfall monitoring system, resulting in the measured values deviating from the actual sediment concentration and forming a superposition of systematic and random errors. Therefore, the joint effect of multiple disturbance sources needs to be fully considered in the actual measurement process in order to establish a more adaptive error compensation model.

The individual error sources analyzed above can be traced to the following physical origins: creep (Equation (2)), vibration (Equations (3)–(5)), and temperature effects (Equations (6) and (7)). Each of these mechanisms independently contributes to the total measurement error. Since the sensor output is the linear superposition of forces/masses and the error terms originate from independent physical processes (mechanical relaxation, external excitation, and thermal response), the total systematic error can be expressed as the sum of their individual contributions. In addition, residual errors due to rapid fluctuations in rainfall intensity are incorporated as an empirical dynamic term. Consequently, the composite error model is formulated in Equation (8), where each term corresponds to a specific error source identified in the preceding sections.

(9) $$E=R_{1}+R_{2}M+vP+t_{1}T+t_{2}a^{dT}+r_{1}b^{dP}$$

where $R_{1}$ and $R_{2}$ are random values in different intervals, v, representing the coefficients of the vibration error; $t_{1}$ and $t_{2}$ represent two different coefficients of the temperature error; and 0 < a < 1 and b > 1 are used to characterize the amplitude modulation of the error under different temperature and shock conditions. The range of values for these parameters can be determined by fitting the actual data, so that the error can be modeled and compensated more accurately.

In order to verify the generality and practical adaptability of the constructed error model, experiments were conducted to compensate and predict the error by introducing data-driven methods under different rainfall conditions and temperatures. Especially for the nonlinear interference characteristics such as high-frequency perturbation and temperature drift, the traditional linear filters such as the Kalman filter have a certain smoothing effect, but their response ability is still limited under multi-factor coupling scenarios. Therefore, this paper introduces the BP neural network to establish a data-driven error compensation model and learn the mapping relationship between the measurement error and the environmental factors through the training model to improve the nonlinear expression ability of the compensation model.

In addition, in order to overcome the problems of the BP neural network easily falling into local optima and slow convergence, this paper introduces GA, PSO and GOOSE to optimize the parameters of the BP network for global search. Especially for the high-dimensional search space and complex disturbance inputs, this paper further proposes ADGOOSE, which enhances the algorithm’s global search and local convergence ability through adaptive parameter regulation and a disturbance restart mechanism.

In the following sections, we will systematically compare the effects of different optimization methods on the error compensation ability of the BP network; analyze its performance under the indicators of RMSE, MAE and R2; and verify the superiority of the adaptive optimization strategy under the dynamic working conditions through experimental data.

### 2.5. Different Filter Compensation Principles

#### 2.5.1. Wavelet Filter and Kalman Filter After Preprocessing

(10) $$H_{filter}\left(k\right)=\sum_{i=k-(L_{w}-1)/2}^{k+(L_{w}-1)/2}c_{i}H_{LS}\left(i\right);i=0,\;1,\cdots \;,N-1$$

where *c_i_* is the weighting factor, taken as 1/10.

The equations of state and observation for the stochastic linear discretization of the Kalman filter are

(11) $$x_{k+1}=\Phi_{k+1/k}x_{k}+\Gamma_{k}u_{k}+G_{k}w_{k}$$

(12) $$y_{k}=C_{k}x_{k}+v_{k}$$

where *x_k_* is the state vector of the system, *y_k_* is the observation sequence of the system, *w_k_* is the process noise sequence of the system, *v_k_* is the observation noise sequence, *u_k_* is the control input of the system, *Φ_k_*_+1_/k is the state transfer matrix, *Γ_k_* and *G_k_* are the coefficient matrices, and *C_k_* is the observation matrix.

After several sets of parameter selection and comparison, the process noise is finally set to Q1 = 0.01; the measurement noise is set to Q2 = 0.1.

#### 2.5.2. The BP Neural Network and Optimization Algorithm

The BP neural network error compensation model consists of an input layer, a hidden layer and an output layer, with full connections established between neuron layers using weights and no connections between neurons in the same layer. The number of layers in the input layer and the output layer is one and only one; the hidden layer can have more than one; and the dataset characteristics and the number of target datasets determine the number of neurons in the input layer and output layer, respectively. After one set of data are preprocessed and input into the input layer, the input data are calculated by the hidden layer and output by the output layer, comparing the difference between the output data and the expected data, so that the actual error is transmitted in the reverse direction along the direction of error reduction, i.e., gradient descent, and a cycle of iterating the weights and thresholds layer by layer to improve the model is performed, so as to make the deviation of the output value satisfy the error of the expected value. The mean square error is a general performance indicator in the BP neural network, which is calculated as in the following equation:

(13) $$E=\frac{1}{n}\sum_{s=1}^{n}{\left(yP_{s}-yD_{s}\right)}^{2}$$

The input parameters are the current weighing bucket sensor voltage, *V_P_*; the sensor voltage of the funnel with its support, *V_D_*; the temperature sensor voltage, *V_T_*; and the output fitted, *V_C_*.

To ensure unbiased evaluation and generalizability, stratified sampling was employed to partition the dataset into a training set (70%), a validation set (15%), and a test set (15%). Stratification was performed based on rainfall intensity and temperature, ensuring a consistent distribution of environmental conditions across each subset. This approach mitigates evaluation bias arising from disparities in data distribution.

Configure the BP neural network structure with five input nodes, one output training value, and 10 hidden layer nodes. The BP neural network process involves initialization: randomly setting weights, w, and bias, b; iterative training: forward propagation to compute layer outputs; backpropagation to calculate error and gradients; parameter updating to adjust w and b; and termination upon reaching the maximum iteration count or loss convergence. The ReLU activation function is selected for the BP neural network model training.

PSO has the advantages of fast convergence, few parameters, and a simple and easy-to-implement algorithm (for high-dimensional optimization problems, it converges to the optimal solution faster than the genetic algorithm), but it also suffers from the problem of falling into a locally optimal solution and therefore relies on a good initialization [26].

(14) $$v_{i}^{d}=\omega v_{i}^{d-1}+c_{1}r_{1}\left({pbest}_{i}^{d}-x_{i}^{d}\right)+c_{2}r_{2}\left({gbest}^{d}-x_{i}^{d}\right)$$

where $v_{i}^{d}$ refers to the speed of the dth step; $v_{i}^{d-1}$ refers to the speed of the previous step; ${pbest}_{i}^{d}$ refers to the best position that the ith particle passes through until the dth iteration; ${gbest}^{d}$ refers to the best position that all the particles pass through until the dth iteration; $x_{i}^{d}$ refers to the dth iteration, the position where the ith particle is located; and *c*_1_ and *c*_2_ are the individual and social acceleration factors of the particles, respectively.

(1) Initialize the parameters such as the maximum number of iterations, population size, individual learning factor, social learning factor, and inertia weight; initialize the position of the particle swarm; and calculate the initial particle fitness to obtain the initial optimal particle.
(2) Calculate the population fitness and update the position of the current particle swarm optimal particle.
(3) Update the position and fitness of the global optimal particle so far.
(4) Loop steps (2)~(3) until the optimal individual position and optimal fitness are obtained, and jump out of the loop. After a finite number of iterations, each particle in the particle swarm will approach the optimal solution.

In this study, the initial swarm size was set at 30, the maximum number of iterations at 1000, the inertia weight at 0.6, and *c*_1_ and *c*_2_ both at 2.

GA is a global optimization algorithm that simulates natural selection and genetic mechanisms, and it is particularly suitable for solving complex nonlinear, multi-peaked, and non-convex optimization problems. GA readily approaches the optimal solution through population evolution, which involves a population composed of a set of potential solutions (individuals) undergoing selection, crossover, and mutation operations in continuous iterations, each generation exhibiting superiority and inferiority, with a view to obtaining the globally optimal solution [27].

First of all, the individual coding the variable to be optimized will be coded as a chromosome, which can be used more widely with number coding; the variable itself is a chromosome, so there is no need to encode/decode; high expression accuracy depends on the original data such that limitation of the number of binary digits can be avoided; and a fast convergence speed and smoother changes are possible in the continuous search space.

In this study, the corresponding fitness function is chosen to measure the quality of an individual; fitness determines the individual genetic probability through the roulette selection method, and the roulette selection probability formula is as follows:

(15) $$p_{i}=\frac{f_{i}}{\sum_{j=1}^{N}f_{j}}$$

where $p_{i}$ is the probability that the *i*th individual is selected.

The crossover operation is then performed to simulate genetic combinations to generate new offspring, and real number coding generally uses linearizable crossover. Finally, the mutation operation is performed to simulate genetic mutation to break the homogeneity of the population, introduce new genetic information, increase diversity, and jump out of the local optimum. Real number coding is generally performed to add a perturbation term; this study uses Gaussian variation, such that the probability of a small perturbation is large and the probability of a large perturbation is small, which is in line with the natural perturbation law.

The termination conditions are then based on whether the maximum number of iterations has been reached, whether the change in the optimal solution is less than a threshold, or whether the average fitness is no longer improving. In this study, the initial population size was set at 30, the maximum number of iterations at 1000, the crossover probability at 0.7, and the mutation probability at 0.02.

GOOSE is a population intelligent optimization algorithm. Rebwar Khalid Hamad proposed a novel meta-heuristic algorithm based on the resting and foraging behavior of geese. Geese stand on one leg and maintain balance to protect and defend other individuals in the flock. The algorithm is characterized by fast convergence and high solution accuracy by adaptively adjusting the resolution of the search space and the search speed in order to find the optimal solution quickly and accurately [28].

Initial development stage:

(16) $$F\_F\_S=T\_o\_A\_O_{it}\;*\;\frac{\sqrt[{2}]{S\_W_{it}}}{9.81}$$

where $T\_o\_A_{O_{it}}$ is the time it takes for the goose’s stone to reach the earth as it falls, $S\_W_{it}$ is the weight of the stone that the goose has stored in its feet, and F_F_S is the speed at which the goose’s stone falls.

(17) $$X_{\left(it+1\right)}=F\_F\_S+D\_G_{it}\;*\;T\_A^{2}$$

where $X_{it}$ refers to the location of the goose, $D\_G_{it}$ refers to the distance of the goose, and T_A refers to the response time of the goose.

Exploration phase:

(18) $$X_{\left(it+1\right)}=randn\left(1,dim\right)*\left(M_{T}\;*\;alpha\right)+Best\_pos$$

(19) $$alpha=(2-(\frac{loop}{\frac{Max\_It}{2}}))$$

where dim is the number of dimensions; Best_pos refers to the best position found; and the value of alpha ranges from 2 to 0, decreasing with the number of iterations.

In this study, the number of iterations is optimized by an adaptive mechanism, which sets a threshold for the population diversity and reduces the number of iterations when the population diversity (e.g., inter-individual distance or fitness difference) is below the threshold.

Adaptive Improvement Design of the GOOSE Algorithm

In order to improve the search ability and convergence accuracy of the GOOSE optimization algorithm in high-dimensional complex problems, this paper carries out a multifaceted design of the adaptive mechanism of the original algorithm, which mainly includes adaptive control parameter adjustments, individual behavior dynamic selection, and step size updating based on adaptive feedback.

This study utilizes the chaotic Cubic map to adjust the quality of the initial population, thereby influencing the algorithm’s exploration and convergence behavior within the search space. Not only does this approach serve as an alternative to traditional random initialization, enhancing population diversity, but it also employs a chaotic search strategy to escape local optima. The Cubic map equations are presented below.

(20) $$Z_{k+1}=\rho Z_{k}(1-{Z_{k}}^{2})$$

Adaptive Control Parameter Mechanism

The search adjustment factor, coe, in the original algorithm is a stochastic parameter with a fixed value limitation, which fails to reflect the change of the search demand in the iterative process. In this paper, by designing a linear decreasing function, coe is dynamically adjusted with the number of iterations, so that the more iterations, the more coe converges to 0.17; this method helps to enhance the global exploration ability in the early stage and focus on local development in the later stage so as to improve the overall convergence performance. The expression is

(21) $$coe(t)=0.17+0.83\cdot (1-\frac{t}{Max\_IT})$$

where coe(t) is the adaptive search regulator, Max_IT represents the maximum number of iterations, and t is the current number of iterations.

Dynamic Selection Mechanism for Individual Behavior

In response to the problem that the selection of individual behavior in the algorithm relies on static probability parameters, an iteration-driven dynamic probability function is introduced for adaptively controlling the ratio of exploitation to exploration. This mechanism makes the algorithm more inclined to random exploration in the early stage and gradually converges to the development near the optimal solution in the later stage, which improves the overall optimization efficiency. In this study, a nonlinear decreasing strategy is chosen, and the probability function’s expression is

(22) $$P(t)=P_{min}+\left(P_{max}-P_{min}\right)\cdot e^{-\varepsilon \cdot \frac{t}{T}}$$

where P(t) represents the behavioral selection probability of the tth generation, *T* represents the maximum number of iterations, and $P_{max}$ and $P_{min}$ represent the behavioral bias parameters at the beginning and the end.

Adaptive Step Update Based on Adaptive Feedback

To avoid the loss of solution accuracy caused by the excessive update amplitude of the excellent individual, an adaptive_differential-driven scaling factor adaptive_factor is introduced, which dynamically adjusts the perturbation amplitude according to the relative difference between the individual’s current fitness and the global optimal fitness. This mechanism ensures the stable evolution of the good solutions, while endowing the bad solutions with stronger exploration capabilities.

To further enhance the ability of the algorithm to jump out of the local extremes in the later search stage, this paper introduces a perturbation-based reinitialization mechanism. This operates by judging that the current population has fallen into a local optimal region when no more optimal solutions appear in successive generations. At this time, some non-elite individuals are randomly selected, and Gaussian perturbation is introduced on the basis of their original positions, so as to artificially break the convergence state of the population and activate the potential solution space region. This strategy effectively improves the diversity of the algorithm and the ability to jump out of the local extreme value without increasing the computational complexity.

In Table 1, it can be observed that removing any of the three components leads to a clear degradation in both RMSE and R^2^, confirming that each modification plays a positive role. The adaptive coefficient has the most pronounced effect on RMSE reduction, as it dynamically balances global exploration and local exploitation throughout the iterations. The perturbation restart mechanism contributes significantly to R^2^ improvement by helping the algorithm escape local optima, thereby enhancing the model’s generalization capability. The dynamic probability facilitates a smooth transition from exploration to exploitation, which stabilizes convergence. The full ADGOOSE achieves the best overall performance, demonstrating that the three enhancements are complementary and collectively yield a substantial improvement over the standard GOOSE.

**Figure 2:**
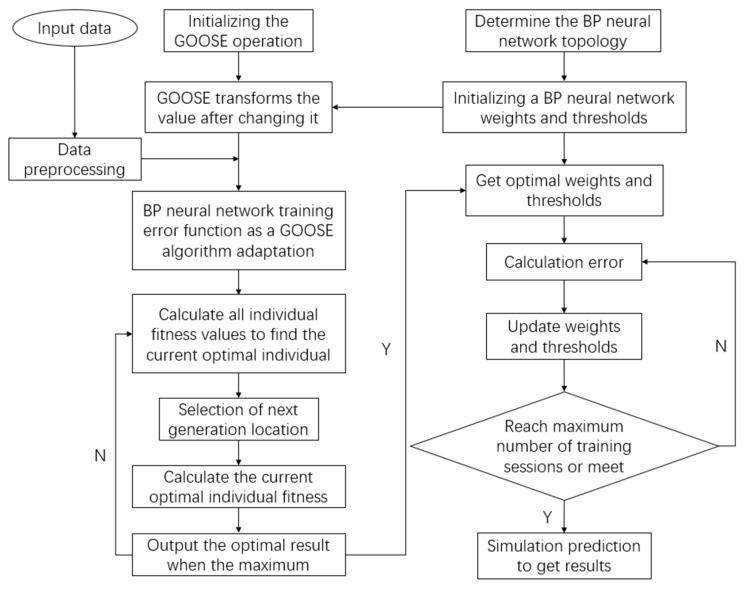
Flowchart of the GOOSE algorithm to optimize the BP network.

In this paper, the improved GOOSE optimization algorithm is used to train and optimize the BP neural network. Firstly, the weights and bias parameters are flattened into vector form as the optimization object. The ADGOOSE with dynamic control, adaptive perturbation and the local restart mechanism is used to search for the parameter combination that minimizes the mean square error (RMSE). In each iteration, the current individual solution is mapped to the BP network weights, and its prediction error is calculated and used as the fitness value. The final output of the optimal parameters is used to construct the BP neural network and for the performance evaluation on the test set. The flow chart of ADGOOSE’s optimization of the BP network is shown in Figure 2.

Table 2 presents a performance comparison of different optimization algorithms applied to BP neural network training. Compared with the standard BP algorithm, both GOOSE and its adaptive version (ADGOOSE) demonstrate improved convergence speed and accuracy. Notably, the proposed ADGOOSE-BP maintains strong global search capability while effectively enhancing convergence stability and efficiency through a dynamic regulation mechanism. This makes it particularly suitable for modeling tasks that are high-dimensional, multi-modal, and nonlinear in nature.

**Figure 3:**
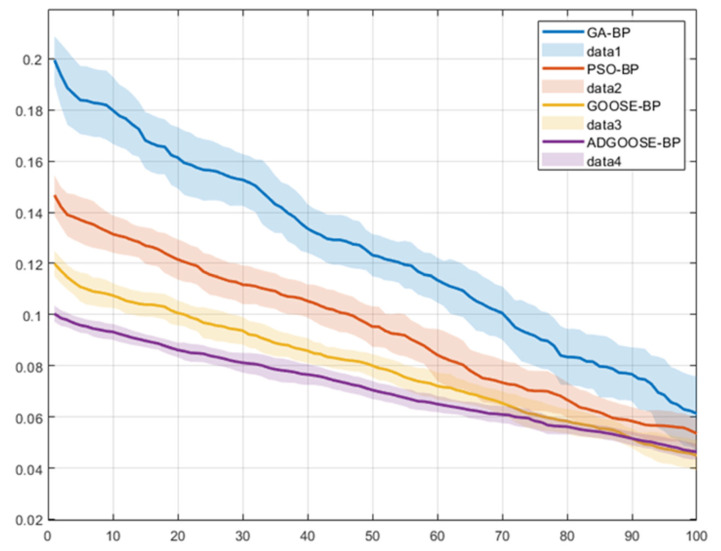
Comparison of convergence curves for different optimization algorithms.

Figure 3 shows the curves depicting the variation in the fitness value (RMSE) over the number of iterations for GA, PSO, GOOSE and ADGOOSE during the optimization of the BP neural network. Each algorithm was run independently 30 times. The solid lines in the figure represent the mean fitness of each generation, whilst the shaded areas indicate ±1 standard deviation, which is used to characterize the convergence stability of the algorithms.

## 3. Experimental Simulation Verification

### 3.1. Device Introduction

An upper load cell is connected to a tray, a lower cell is connected to a support plate, the tray holding up a bucket fitted with a shell whose opening has a diameter of 200, while another support plate is connected to a third support plate with leveling bolts, as shown in the figure, and this device is used to measure and record precipitation.

**Figure 4:**
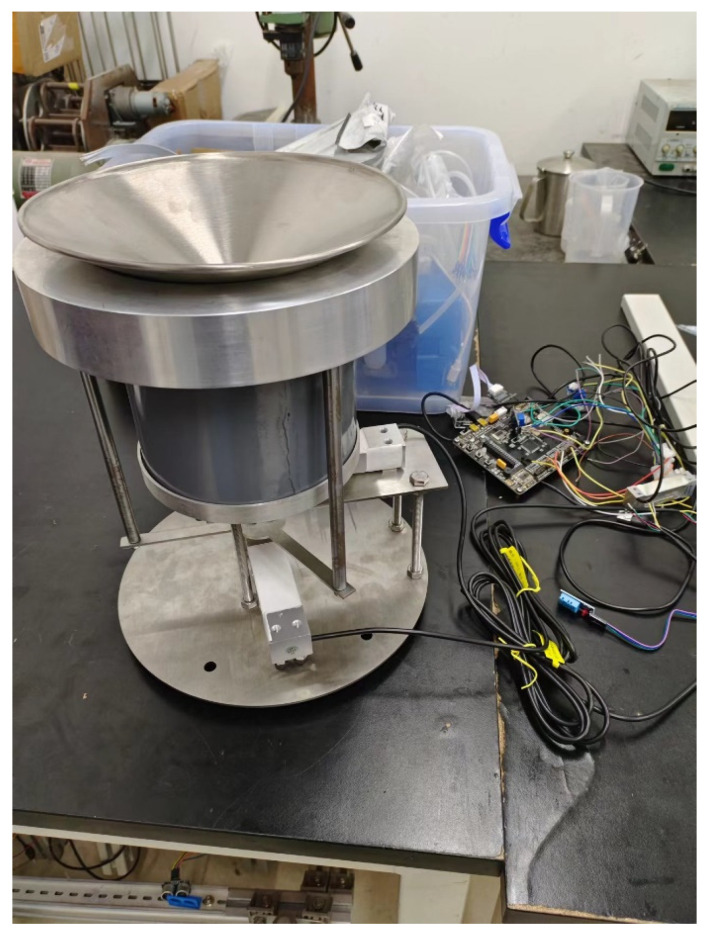
Photograph of the device.

A rainfall simulation system with manually adjustable rainfall intensity was designed using an infinitely variable peristaltic pump that can be adjusted by the knob on the adapter to adjust the size of the flow rate (12–100 mL/min), and the current flow rate is displayed through the flowmeter connected to it. The outlet of the flowmeter flows into the funnel, the outlet of the funnel flows into the bucket, the weight change of the funnel and the bucket is transmitted to the control board through the voltage of the load cell, and the control board will filter and process the received signals and then convert the analog quantity into a digital quantity and send it to the upper computer on the computer terminal. Figure 4 is a photograph of the device.

### 3.2. Load-Cell Calibration

The voltage data measured at different weights were preprocessed, as shown in the table below, and the calibrated fitted first-order linear functions corresponding to multiple sets of data for multiple weights are shown below. The RMSE values for the error between the fitted curves and the data itself for the first three sets of data are 0.01237, 0.01412 and 0.01485, respectively; since the direction and magnitude of the error after creep is unpredictable, the average of the multiple sets of data was taken as the final calibration datum, and the RMSE error of the data was reduced to 0.0094. Table 3 shows the weighing data.

### 3.3. Vibration Errors for Different Rainfall Intensities

**Figure 5:**
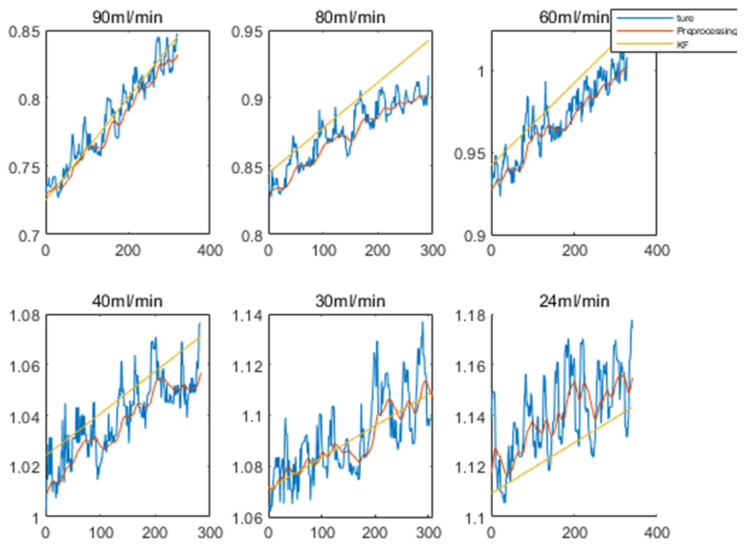
Measured data, preprocessed data and Kalman-filtered data under different rainfall intensities.

The following figure shows the initial data, preprocessed data and Kalman-filtered processed data for rainfall intensities of 24 mL/min, 30 mL/min, 40 mL/min, 60 mL/min, 80 mL/min, and 90 mL/min. The sampling period of the data is 0.5 s, and the amount of data collected in each group is 300–350 measurements. Figure 5 shows the data under different rainfall intensities.

With the increase in rainfall intensity, the high-frequency fluctuation and mutation values in the initial measurement data increased significantly, especially under the conditions of 80 mL/min and 90 mL/min, and the interference amplitude in the original data reached the maximum. Through preprocessing and Kalman filtering, the measurement noise can be significantly suppressed, the data stability can be improved, the filtered curves can be made smoother, and the trend changes can be made more in line with the physical process.

Based on the above experimental data, some parameters in Equation (8) were inversely derived to obtain their optimal fitting range, and representative parameter combinations were screened on this basis. It was finally determined that v = −0.0014, $r_{1}$ = 0.6, and b = 0.8 were the best values for the overall optimization and had good generalization ability under different rainfall intensities.

### 3.4. Vibration Errors for Different Temperatures

**Figure 6:**
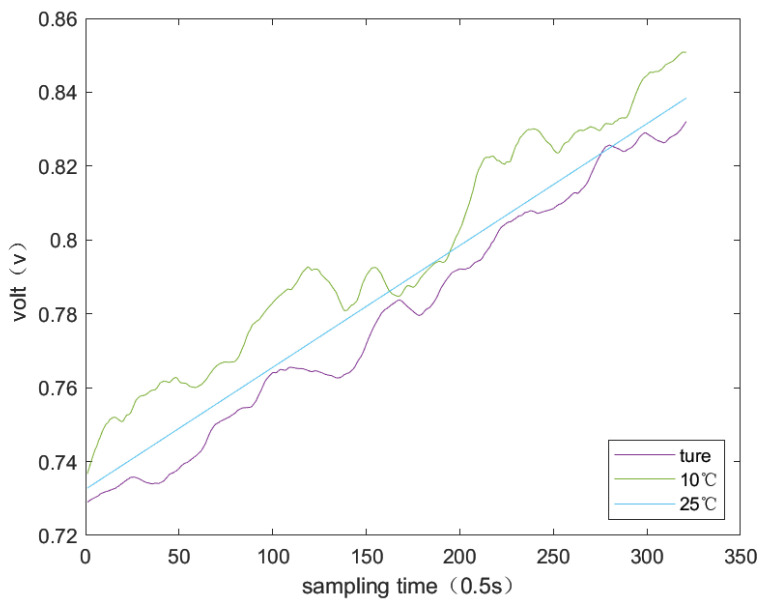
Rainfall data at different temperatures at 90 mL/min.

The rainfall measurements at different temperatures (10 °C and 25 °C) at 90 mL/min are shown in Figure 6.

The figure above shows the rainfall measurements collected under different temperature conditions. The results show that temperature changes mainly caused baseline drift and sensitivity changes in the measurements. Under the condition of 25 °C, the measurement curves are uplifted as a whole, and more systematic deviations appear. The analyzed results verify the validity of the nonlinear enhancement coefficient set in the temperature error model.

Based on the above experimental data, $t_{1}$, $t_{2}$, and a in Equation (8) were deduced inversely to obtain their optimal fitting ranges, and representative parameter combinations were screened on this basis. It was finally determined that selecting $t_{1}$ = −0.01, $t_{2}$ = 0.3, and a = 0.5 is better for overall optimization.

Combined with the rainfall intensity and temperature experimental results, this paper corrected and fitted the comprehensive error modeling formula and finally obtained the following general error compensation expression:

(23) $$E=R_{1}+R_{2}M-0.0014P-0.01T+0.3\;*\;0.5^{dT}+0.6\;*\;0.8^{dP}$$

### 3.5. Variable-Flow Experiment

The data were sampled with a sampling period of 0.5 s and at least a 2.5 min per measurement period. The initial data collected were preprocessed and then errors were compensated for using Kalman filtering, a basic BP neural network, and a BP network model after the introduction of several intelligent optimization algorithms.

**Figure 7:**
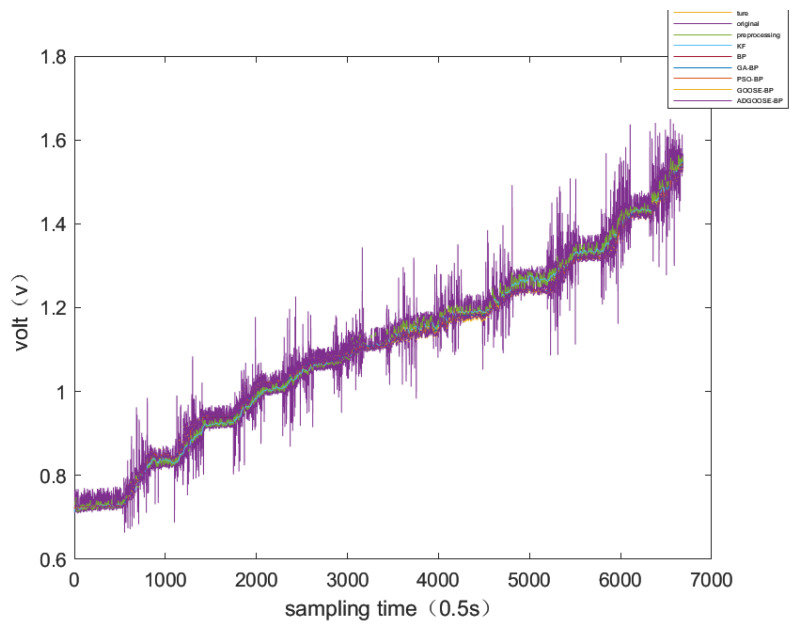
Comparison of overall data.

The overall compensation effect is reflected in the 90 mL/min_80 mL/min_60 mL/min_40 mL/min_30 mL/min_24 mL/min_30 mL/min_40 mL/min_60 mL/min_80 mL/min_90 mL/min variable-flow-rate experiments, and the effect graph is shown in Figure 7.

The entire variable-flow-rate experiment was split into two working conditions: 90–80–60–40–30–24 and 24–30–40–60–80–90 (mL/min). The performance of various error compensation methods for rainfall measurement tasks was assessed accordingly.

In order to comprehensively evaluate the performance of different error compensation methods in rainfall measurement tasks, this paper systematically compares the preprocessing methods, Kalman filtering, the basic BP neural network, and the BP network model after the introduction of multiple intelligent optimization algorithms. The evaluation indexes include the root mean square error (RMSE), the mean absolute error (MAE), and the coefficient of determination (R2).

The traditional preprocessing and Kalman filtering methods improved the data quality to some extent, but their prediction errors were still at a high level. After the introduction of the underlying BP neural network, the model performance was significantly improved, with the RMSE decreasing to 0.0921/0.1104 and the R2 reaching 0.9054/0.8985, indicating that the neural network possesses a significant advantage in nonlinear mapping modeling.

Further, the introduction of GA and PSO significantly improves the global searching ability of the BP network, and the prediction error is further reduced. Although PSO-BP outperforms GA-BP in terms of RMSE, it is slightly lower in terms of R2, and there is some risk of local convergence.

The BP network optimized with GOOSE improves the model stability and global search performance while maintaining a lower prediction error. And on this basis, the adaptive GOOSE proposed in this paper further compresses the error space by introducing the dynamic parameter control mechanism, adaptive perturbation restart, the elite bootstrapping and mutation strategy, and other improvements, which reduces the RMSE to 0.0494/0.0543, while the R2 reaches as high as 0.9835/0.9789; this demonstrates the optimal overall fitting ability and generalization performance. Table 4 and Table 5 are comparison tables of the compensation effects of various methods of processing.

From the table, it can be seen that with the gradual enhancement of the optimization strategy, the effect of error compensation continues to improve. Preprocessing and Kalman filtering, as traditional methods, improve the data quality to a certain extent, but there is still a high prediction error. After the adoption of the BP neural network, the error is significantly reduced, and the model performance is significantly enhanced after the introduction of intelligent optimization strategies (e.g., GA, PSO, and GOOSE). Among them, the adaptive GOOSE-optimized BP network (ADGOOSE-BP) exhibits the optimal integrated prediction performance with the lowest RMSE (0.0494/0.0543) and the highest coefficient of determination (0.9835/0.9789), which verifies the effectiveness and superiority of the proposed improved method in the error compensation task.

Table 6 summarizes the mean values, standard deviations, and 95% confidence intervals for RMSE for all methods based on the 30 runs.

It can be observed that the RMSE improvement of ADGOOSE-BP over GOOSE-BP is approximately 7–8% on average, and the 95% confidence intervals do not overlap, indicating a statistically meaningful difference.

Regarding the cross-check against actual data, actual rainfall refers to the total volume of water collected, as measured by a high-precision electronic balance (with a resolution of 0.01 grammes) positioned beneath the collection system. This measured mass value serves as an absolute reference and is independent of the load-cell reading. The comparison was performed for the entire variable-flow sequence (24→90 and 90→24 mL/min combined, total duration: 25 min). The ADGOOSE-BP compensated cumulative depth (38.12 mm) is within 0.08 mm (0.21%) of the actual reference (38.20 mm).

This paper establishes a nonlinear error model that comprehensively considers multi-source disturbances, rather than addressing single errors. The study introduces ADGOOSE and other intelligent optimization algorithms, significantly enhancing the convergence performance and compensation accuracy of the BP network, with results superior to those of traditional filtering and common optimization methods such as GA and PSO. Validation in simulated environments featuring variable intensity and temperature demonstrates the method’s strong adaptability and robustness under dynamic, complex conditions. This provides an effective software solution that demonstrates strong potential as a high-precision, adaptive compensation framework.

## 4. Conclusions

This paper focuses on the study of the error sources, modeling methods and compensation strategies of weighing rain gauges in complex environments, focusing on the analysis of the formation mechanism of five major error factors, namely, creep error, vibration interference, temperature drift, residual error and random error, and using experimental data to construct an environmentally adaptable error mathematical model. On this basis, three types of error compensation schemes are designed, including a traditional filtering method (Kalman filtering), a standard BP neural network, and a BP neural network model optimized by various intelligent optimization algorithms (GA, PSO, GOOSE and ADGOOSE).

The experimental results show that:

1. Under different rainfall intensity and temperature-change conditions, the original data reveal obvious high frequency fluctuations and system drift. Traditional filtering means (such as Kalman filtering) can improve measurement accuracy to some extent, but they find it difficult to adequately model complex perturbation characteristics.
2. The BP neural network has a good nonlinear fitting ability and is better than the traditional methods in terms of modeling and error compensation, but the stability of convergence is limited and it easily falls into local optima.
3. The introduction of intelligent algorithms, such as GA and particle swarm optimization, can effectively improve the performance of the BP network, and the optimized model significantly outperforms the basic network in several evaluation indexes (RMSE, MAE, and R^2^).
4. The ADGOOSE optimization algorithm proposed in this paper combines dynamic search regulation, a perturbation restart mechanism, and an elite bootstrap strategy, and it can significantly improve the convergence speed and robustness of the global model, while the error rate is higher than that of the traditional method. Its speed and robustness, as well as its error compensation performance, were optimized in multiple rounds of experiments.

The results show that the adaptive GOOSE-BP error compensation model not only achieves the lowest RMSE and the highest R^2^ on the simulation data, but also that it has good generalization ability and practical deployment potential. The research results can provide a theoretical basis and engineering reference for error compensation and intelligent optimization of high-precision rainfall monitoring equipment in the future.

## 5. Discussion

It should be noted that Equation (8) assumes additive independence among error sources. While this simplification is physically justified by the linear superposition principle of load-cell outputs and the independence of the underlying mechanisms, it does not explicitly account for potential cross-coupling effects (e.g., temperature-dependent creep). However, the experimental validation under dynamically varying temperature and flow conditions (Section 3.4 and Section 3.5) demonstrates that the independent model, combined with ADGOOSE-BP optimization, provides sufficient accuracy and robustness for practical hydrological monitoring. Future work will explore the incorporation of coupled terms using more advanced identification techniques such as NARX or Gaussian process regression.

It should be noted that the temperature compensation model (Equation (8)) was validated only at 10 °C and 25 °C, which represent typical ambient temperatures for rainfall monitoring in temperate regions. The exponential term was derived from the well-established temperature-dependent resistance characteristics of strain gauges (see Section 2.3.3), and the fitted parameters are consistent with the sensor’s datasheet behavior. Within the tested range, the model effectively captures the baseline drift and sensitivity changes. However, the model’s performance outside this range has not been experimentally verified; for temperatures below 10 °C or above 25 °C, the compensation may be less accurate due to potential nonlinearities in thermal hysteresis or material property changes. Nevertheless, since the model is data-driven and the BP network can be retrained with additional calibration data, the current framework is adaptable to extended temperature ranges by incorporating more experimental points. Future work will include a systematic temperature sweep from 0 °C to 40 °C to fully validate and, if necessary, recalibrate the model.

In the context of the present study, it is important to clarify that our experiments were conducted under controlled laboratory conditions using a simulated rainfall system without cross-wind. Water was delivered vertically into the collection funnel, and no horizontal wind component was present. Consequently, horizontal precipitation effects were not included in the experimental data, and our error model (Equation (8)) does not contain a specific term for wind-induced horizontal precipitation error. We acknowledge that in real-world field deployment, wind-induced undercatch due to horizontal precipitation would indeed influence measurement accuracy and would require separate consideration.

## Figures and Tables

**Table 1:** Ablation study results for ADGOOSE components.

	90–80–60–40–30–24 mL/min (RMSE/R^2^)	24–30–40–60–80–90 mL/min (RMSE/R^2^)
ADGOOSE	0.0494/0.9835	0.0543/0.9789
GOOSE	0.0531/0.9779	0.0578/0.9721
ADGOOSE-CCM	0.0511/0.9768	0.0552/0.9741
ADGOOSE-AC	0.0537/0.9793	0.0566/0.9754
ADGOOSE-DSM	0.0512/0.9784	0.0545/0.9747
ADGOOSE-ASU	0.0515/0.9778	0.0554/0.9764

**Table 2:** Comparison of the effects of the algorithms.

Algorithm Name	Convergence Rate	Convergence Stability	Computational Complexity (per Generation)	Brief Analysis of Advantages and Disadvantages
KF	Very fast	Comparatively good	Lower	High accuracy but narrow range of application
BP	Moderate	Mediocre	Middle	Prone to localized extremes
GA-BP	Slower	Moderate	Above average	Global and time-consuming
PSO-BP	Relatively fast	Comparatively good	Mid-to-high	Simple and efficient, prone to early convergence affecting the quality of the final optimization
GOOSE-BP	Moderate	Comparatively good	Mid-to-high	Good stability, complex tuning parameters
ADGOOSE-BP	Relatively fast	Rare	Relatively high	Convergent equilibrium, slightly complex structure

**Table 3:** Weighing data table.

W/g	U1/v	U2/v	U3/v	Arg/v
0	0.431	0.4246	0.4278	0.4278
1	0.4294	0.4302	0.4318	0.4305
2	0.4326	0.4351	0.431	0.4329
3	0.4423	0.4342	0.4487	0.4417
4	0.4399	0.4439	0.4536	0.4458
5	0.4471	0.4399	0.4568	0.4479
6	0.4576	0.456	0.4584	0.4573
7	0.4665	0.4455	0.46	0.4573
8	0.4624	0.4479	0.4745	0.4616
9	0.4528	0.489	0.4826	0.4748
10	0.4697	0.485	0.4802	0.4783
20	0.4786	0.5124	0.5204	0.5038
30	0.5503	0.5406	0.5156	0.5355
40	0.5551	0.535	0.5357	0.5419
50	0.564	0.5873	0.597	0.5828
60	0.5889	0.601	0.6188	0.6029
70	0.6123	0.6397	0.6236	0.6252
80	0.655	0.6614	0.6405	0.6523
90	0.6896	0.705	0.6832	0.6926
100	0.705	0.7227	0.7033	0.7103
200	1.0055	0.9732	1.0103	0.9963
300	1.2439	1.2335	1.252	1.2431
400	1.5026	1.513	1.4881	1.5012
500	1.7805	1.7773	1.7596	1.7725

**Table 4:** Error compensation results (90–80–60–40–30–24 mL/min).

Error Compensation Methods	RMSE	MAE	R2
Preprocessing	0.7030	0.5679	0.8710
KF	0.0921	0.0859	0.9054
BP	0.0849	0.0653	0.9302
GA-BP	0.0832	0.0650	0.9487
PSO-BP	0.0564	0.0545	0.9686
GOOSE-BP	0.0531	0.0412	0.9779
ADGOOSE-BP	0.0494	0.0425	0.9835

**Table 5:** Error compensation results (24–30–40–60–80–90 mL/min).

Error Compensation Methods	RMSE	MAE	R2
Preprocessing	0.7598	0.6452	0.8531
KF	0.1104	0.0987	0.8985
BP	0.0864	0.0759	0.9124
GA-BP	0.0845	0.0744	0.9351
PSO-BP	0.0637	0.0593	0.9618
GOOSE-BP	0.0578	0.0486	0.9721
ADGOOSE-BP	0.0543	0.0493	0.9789

**Table 6:** Statistical comparison of RMSE values.

Method	Mean ± Std/95%CI (90–80–60–40–30–24 mL/min)	Mean ± Std/95%CI (24–30–40–60–80–90 mL/min)
GA-BP	0.083 ± 0.003/0.0819–0.0841	0.084 ± 0.003/0.0829–0.0851
PSO-BP	0.056 ± 0.002/0.0553–0.0567	0.064 ± 0.003/0.0629–0.0651
GOOSE-BP	0.053 ± 0.002/0.0523–0.0537	0.058 ± 0.002/0.0573–0.0587
ADGOOSE-BP	0.049 ± 0.0015/0.0485–0.0495	0.054 ± 0.0018/0.0534–0.0546

## Data Availability

All data generated or analyzed during this study are included in the published article and its Supplementary Information Files.

## Supplementary Materials

The following supporting information can be downloaded at https://www.mdpi.com/article/10.3390/s26144654/s1.
