# Supplementary material for: Multi-Source Error Compensation for Weighing Rain Gauge Based on Adaptive GOOSE-BP Network
Source: Sensors (Basel). 2026 Jul 22;26(14):4654. doi: 10.3390/s26144654 (PMC13416980; doi:10.3390/s26144654)
Supplement: Supplementary file 1 [file sensors-26-04654-s001.zip › sensors-4404230-supplementary.pdf]

0.724962659	0.7254435	0.99997
0.725061244	0.7254435	0.999965153
0.725204589	0.7254435	0.99992461
0.72620588	0.7254435	0.999884087
0.72620927	0.7254435	0.999843589
0.72601425	0.7254435	0.999803201
0.729159961	0.7254435	0.999762823
0.729170678	0.7254435	0.999722438
0.729181421	0.7254435	0.999682373
0.72919219	0.7254435	0.999642321
0.726946528	0.7254435	0.999602282
0.725667117	0.7254435	0.999562256
0.725669184	0.7254435	0.999522021
0.725671255	0.7254435	0.999481671
0.725921987	0.7254435	0.999441334
0.727086487	0.7254435	0.999401008
0.727612818	0.7254435	0.999360719
0.739577173	0.7254435	0.999320556
0.7396609	0.7254435	0.999280455
0.731250462	0.7254435	0.999241544
0.731266218	0.7254435	0.999202646
0.731282011	0.7254435	0.999162936
0.727804519	0.7254435	0.999123239
0.725061862	0.7254435	0.999083556
0.72506246	0.7254435	0.999043541
0.72506306	0.7254435	0.999003271
0.725063661	0.7254435	0.998963013
0.725029034	0.7254435	0.99892277
0.725029553	0.7254435	0.99888254
0.725030074	0.7254435	0.998842319
0.725030595	0.7254435	0.99880211
0.725031118	0.7254435	0.998761915
0.725010686	0.7254435	0.998721733
0.725001436	0.7254435	0.998681564
0.725001889	0.7254435	0.998641407
0.725002343	0.7254435	0.998601262
0.725002798	0.7254435	0.998561129
0.725023411	0.7254435	0.998521011
0.725023916	0.7254435	0.998480905
0.725099003	0.7254435	0.998440814
0.72509969	0.7254435	0.998400737
0.725100379	0.7254435	0.998360681
0.725115957	0.7254435	0.998320639
0.725116685	0.7254435	0.99828061

0.725117415	0.7254435	0.998240595
0.725062482	0.7254435	0.998200593
0.725050796	0.7254435	0.998160606
0.725028522	0.7254435	0.998120628
0.725008345	0.7254435	0.998080661
0.724999204	0.7254435	0.998040705
0.725009285	0.7254435	0.99800076
0.725030601	0.7254435	0.997960827
0.725000538	0.7254435	0.997920908
0.725000989	0.7254435	0.997881005
0.725021405	0.7254435	0.997841111
0.7250327	0.7254435	0.99780123
0.725033228	0.7254435	0.997761365
0.725069756	0.7254435	0.997721514
0.72511235	0.7254435	0.997681677
0.725113069	0.7254435	0.997641858
0.725113791	0.7254435	0.997602058
0.725099697	0.7254435	0.997562275
0.725243107	0.7254435	0.997522507
0.726127265	0.7254435	0.997482753
0.726130462	0.7254435	0.997443031
0.726133666	0.7254435	0.997403412
0.726136879	0.7254435	0.997363811
0.725373308	0.7254435	0.997324228
0.725374658	0.7254435	0.997284664
0.725376013	0.7254435	0.99724504
0.72537737	0.7254435	0.997205434
0.726223201	0.7254435	0.997165845
0.726026211	0.7254435	0.997126272
0.72521227	0.7254435	0.997086799
0.725807708	0.7254435	0.997047324
0.725194461	0.7254435	0.997007786
0.725977223	0.7254435	0.996968323
0.72598005	0.7254435	0.996928817
0.72519722	0.7254435	0.996889404
0.725985725	0.7254435	0.996850008
0.725199071	0.7254435	0.996810553
0.725180968	0.7254435	0.996771192
0.725242085	0.7254435	0.996731771
0.725243118	0.7254435	0.996692367
0.725244153	0.7254435	0.996652985
0.725166254	0.7254435	0.996613622
0.725167103	0.7254435	0.996574278
0.725167955	0.7254435	0.996534944

0.726710776	0.7254435	0.996495628
0.725188109	0.7254435	0.996456332
0.726367213	0.7254435	0.996417206
0.726219812	0.7254435	0.99637795
0.725137812	0.7254435	0.996338827
0.72622667	0.7254435	0.996299709
0.725123354	0.7254435	0.996260507
0.7251241	0.7254435	0.996221431
0.725124848	0.7254435	0.996182269
0.725330559	0.7254435	0.996143127
0.725142525	0.7254435	0.996104007
0.725307442	0.7254435	0.996064929
0.72516123	0.7254435	0.996025854
0.725362646	0.7254435	0.995986816
0.725363971	0.7254435	0.995947785
0.725163748	0.7254435	0.995908795
0.72536663	0.7254435	0.995869825
0.726344714	0.7254435	0.995830857
0.725887864	0.7254435	0.995791929
0.725292261	0.7254435	0.995753117
0.726136946	0.7254435	0.995714279
0.725294572	0.7254435	0.9956754
0.726143394	0.7254435	0.995636625
0.725273072	0.7254435	0.995597785
0.725323152	0.7254435	0.995559049
0.726153127	0.7254435	0.995520247
0.725409493	0.7254435	0.995481469
0.725326849	0.7254435	0.995442795
0.725412374	0.7254435	0.99540407
0.725329328	0.7254435	0.995365357
0.725330572	0.7254435	0.995326674
0.725331819	0.7254435	0.995288004
0.725333069	0.7254435	0.995249353
0.725334322	0.7254435	0.995210724
0.725219064	0.7254435	0.995172116
0.725220041	0.7254435	0.995133529
0.725059546	0.7254435	0.995094952
0.725147337	0.7254435	0.995056394
0.725148141	0.7254435	0.995017839
0.725184563	0.7254435	0.994979312
0.725185457	0.7254435	0.994940803
0.725186353	0.7254435	0.994902317
0.725187251	0.7254435	0.99486385
0.725898361	0.7254435	0.9948254

0.725900994	0.7254435	0.994786969
0.725189958	0.7254435	0.994748624
0.725705326	0.7254435	0.994710298
0.727172156	0.7254435	0.994671919
0.725623147	0.7254435	0.994633607
0.727183743	0.7254435	0.994595458
0.725586882	0.7254435	0.994557174
0.72591961	0.7254435	0.994519061
0.725922295	0.7254435	0.99448081
0.725307498	0.7254435	0.99444261
0.725927686	0.7254435	0.99440443
0.725930391	0.7254435	0.994366207
0.72531108	0.7254435	0.994328064
0.72531228	0.7254435	0.994289941
0.725997249	0.7254435	0.994251777
0.725314688	0.7254435	0.994213634
0.725646977	0.7254435	0.994175579
0.726352366	0.7254435	0.994137477
0.725463593	0.7254435	0.994099428
0.726359879	0.7254435	0.99406147
0.725434487	0.7254435	0.994023445
0.72636743	0.7254435	0.993985529
0.72637122	0.7254435	0.993947544
0.725852166	0.7254435	0.993909672
0.725854686	0.7254435	0.993871822
0.72646499	0.7254435	0.993833942
0.726386471	0.7254435	0.993796085
0.726311735	0.7254435	0.99375831
0.727195448	0.7254435	0.993720551
0.726319048	0.7254435	0.993682807
0.726322718	0.7254435	0.993645173
0.725519121	0.7254435	0.993607474
0.725875002	0.7254435	0.993569799
0.725877578	0.7254435	0.993532069
0.725827328	0.7254435	0.993494398
0.725829787	0.7254435	0.993456751
0.725832252	0.7254435	0.993419124
0.725529417	0.7254435	0.993381522
0.725531147	0.7254435	0.993343946
0.726279402	0.7254435	0.993306363
0.726282974	0.7254435	0.993268805
0.726286554	0.7254435	0.993231345
0.726290144	0.7254435	0.99319391
0.727697619	0.7254435	0.9931565

0.731082446	0.7254435	0.993119114
0.726835642	0.7254435	0.993081892
0.726840592	0.7254435	0.993045028
0.726647594	0.7254435	0.993007768
0.726652077	0.7254435	0.992970534
0.725514091	0.7254435	0.992933306
0.725515785	0.7254435	0.992896102
0.725517482	0.7254435	0.992858811
0.725334427	0.7254435	0.992821544
0.725335683	0.7254435	0.992784301
0.725220122	0.7254435	0.992747066
0.725221102	0.7254435	0.992709856
0.725265494	0.7254435	0.992672661
0.725266583	0.7254435	0.992635491
0.725184614	0.7254435	0.99259835
0.725117507	0.7254435	0.992561234
0.725134016	0.7254435	0.992524134
0.725187302	0.7254435	0.992487053
0.725188203	0.7254435	0.992449999
0.725153051	0.7254435	0.992412977
0.725171494	0.7254435	0.992375982
0.725210465	0.7254435	0.99233901
0.725803179	0.7254435	0.992302066
0.725174086	0.7254435	0.992265153
0.725213341	0.7254435	0.992228327
0.725917084	0.7254435	0.992191468
0.726038308	0.7254435	0.992154641
0.725306378	0.7254435	0.992117915
0.725217208	0.7254435	0.99208123
0.72521818	0.7254435	0.992044503
0.725309951	0.7254435	0.992007796
0.725311149	0.7254435	0.99197112
0.725221112	0.7254435	0.991934484
0.725313551	0.7254435	0.991897878
0.725603944	0.7254435	0.991861291
0.725267687	0.7254435	0.991824744
0.725462116	0.7254435	0.991788253
0.725949674	0.7254435	0.991751759
0.725952434	0.7254435	0.991715312
0.726143731	0.7254435	0.991678941
0.726146968	0.7254435	0.991642599
0.725577721	0.7254435	0.991606303
0.726153466	0.7254435	0.991570036
0.725581423	0.7254435	0.991533739

0.72532701	0.7254435	0.991497528
0.72571197	0.7254435	0.99146129
0.725414008	0.7254435	0.991425057
0.725415458	0.7254435	0.99138889
0.726105615	0.7254435	0.991352723
0.725418368	0.7254435	0.991316586
0.725419828	0.7254435	0.991280544
0.725421292	0.7254435	0.991244464
0.725422759	0.7254435	0.991208411
0.725455903	0.7254435	0.991172389
0.725457455	0.7254435	0.991136395
0.72545901	0.7254435	0.991100432
0.72600328	0.7254435	0.991064499
0.725399681	0.7254435	0.991028594
0.725401096	0.7254435	0.990992771
0.725653181	0.7254435	0.990956918
0.725655214	0.7254435	0.990921096
0.726821148	0.7254435	0.990885327
0.726826061	0.7254435	0.990849586
0.726830987	0.7254435	0.99081399
0.728565501	0.7254435	0.990778421
0.72857475	0.7254435	0.990742881
0.727065538	0.7254435	0.990707539
0.727733489	0.7254435	0.990672226
0.730842558	0.7254435	0.990636791
0.727747872	0.7254435	0.990601448
0.727755091	0.7254435	0.990566438
0.726489601	0.7254435	0.990531151
0.726493693	0.7254435	0.990495892
0.726497795	0.7254435	0.990460537
0.725524411	0.7254435	0.99042521
0.725182884	0.7254435	0.990389913
0.725266646	0.7254435	0.990354549
0.725267738	0.7254435	0.990319183
0.725268833	0.7254435	0.990283856
0.72526993	0.7254435	0.99024856
0.72537354	0.7254435	0.990213295
0.725374891	0.7254435	0.99017806
0.725376246	0.7254435	0.990142866
0.725377604	0.7254435	0.990107703
0.726086982	0.7254435	0.990072571
0.729426746	0.7254435	0.990037471
0.729438117	0.7254435	0.990002471
0.727065595	0.7254435	0.989967829

0.728403054	0.7254435	0.989933217
0.728411901	0.7254435	0.989898401
0.72842077	0.7254435	0.989863747
0.72842966	0.7254435	0.989829124
0.726251415	0.7254435	0.989794533
0.726254918	0.7254435	0.989759975
0.725774992	0.7254435	0.989725233
0.725561391	0.7254435	0.989690522
0.725779659	0.7254435	0.989655795
0.725782001	0.7254435	0.989621079
0.725566829	0.7254435	0.989586415
0.725568651	0.7254435	0.989551782
0.725497513	0.7254435	0.98951716
0.725499165	0.7254435	0.98948257
0.725500822	0.7254435	0.989448002
0.725615599	0.7254435	0.989413465
0.727043704	0.7254435	0.989378958
0.727049171	0.7254435	0.989344493
0.725621436	0.7254435	0.989310197
0.725623391	0.7254435	0.989275932
0.726234066	0.7254435	0.989241557
0.728403146	0.7254435	0.989207212
0.728411993	0.7254435	0.989172957
0.728420862	0.7254435	0.989138947
0.729035995	0.7254435	0.989104967
0.729046406	0.7254435	0.989071019
0.726115167	0.7254435	0.989037162
0.726118334	0.7254435	0.989003339
0.729077795	0.7254435	0.988969258
0.726891168	0.7254435	0.98893521
0.725459099	0.7254435	0.988901487
0.725460659	0.7254435	0.98886758
0.726801892	0.7254435	0.988833566
0.726806758	0.7254435	0.988799585
0.725499226	0.7254435	0.98876577
0.725500883	0.7254435	0.988731989
0.725502544	0.7254435	0.988698112
0.725504209	0.7254435	0.988664269
0.725505878	0.7254435	0.988630457
0.725581548	0.7254435	0.988596677
0.725509229	0.7254435	0.988562928
0.72541266	0.7254435	0.988529219
0.725414106	0.7254435	0.988495534
0.725514285	0.7254435	0.988461872

0.725481279	0.7254350	.988428241
0.725333317	0.7254350	.988394651
0.725484509	0.7254350	.988361089
0.725637284	0.7254350	.988327541
0.726777792	0.7254350	.98829404
0.725524509	0.7254350	.988260585
0.72564328	0.7254350	.988227274
0.725734126	0.7254350	.988193871
0.725736357	0.7254350	.988160512
0.725738593	0.7254350	.988127192
0.725740835	0.7254350	.988093905
0.725743082	0.7254350	.988060649
0.725844981	0.7254350	.988027426
0.725847484	0.7254350	.987994236
0.725849992	0.7254350	.987961088
0.725852507	0.7254350	.987927972
0.726026809	0.7254350	.987894889
0.726160252	0.7254350	.987861839
0.726032715	0.7254350	.987828838
0.726166816	0.7254350	.987795881
0.726657017	0.7254350	.987762942
0.726661523	0.7254350	.987730049
0.726666041	0.7254350	.987697234
0.72667057	0.7254350	.987664451
0.72667511	0.7254350	.9876317
0.726679661	0.7254350	.98759898
0.726121657	0.7254350	.987566292
0.72612484	0.7254350	.987533635
0.727506952	0.7254350	.987500953
0.726200212	0.7254350	.987468302
0.725462295	0.7254350	.987435818
0.726206974	0.7254350	.987403236
0.72852031	0.7254350	.987370611
0.726144101	0.7254350	.98733809
0.72540551	0.7254350	.987305827
0.726150586	0.7254350	.987273325
0.726087192	0.7254350	.987240745
0.725409808	0.7254350	.987208234
0.725411247	0.7254350	.987175711
0.72541269	0.7254350	.987143116
0.725414136	0.7254350	.987110515
0.725415586	0.7254350	.987077909
0.72541704	0.7254350	.987045297
0.725449911	0.7254350	.987012681

0.726180162	0.7254435	0.986980058
0.726183489	0.7254435	0.986947433
0.726258759	0.7254435	0.986914874
0.727359457	0.7254435	0.98688231
0.727365709	0.7254435	0.986849748
0.727371977	0.7254435	0.986817288
0.72737826	0.7254435	0.986784824
0.726802171	0.7254435	0.986752353
0.725950091	0.7254435	0.986719877
0.726811916	0.7254435	0.986687339
0.726816807	0.7254435	0.986654711
0.725958392	0.7254435	0.986622161
0.725961173	0.7254435	0.986589604
0.725963961	0.7254435	0.986556955
0.725966755	0.7254435	0.986524299
0.725545495	0.7254435	0.986491635
0.726308851	0.7254435	0.986458964
0.72922828	0.7254435	0.986426243
0.729239164	0.7254435	0.986393588
0.726319812	0.7254435	0.986361213
0.726323483	0.7254435	0.986328831
0.728439502	0.7254435	0.986296152
0.730307652	0.7254435	0.986263465
0.728457399	0.7254435	0.986230978
0.726338261	0.7254435	0.986198666
0.726341979	0.7254435	0.986166162
0.725941878	0.7254435	0.986133441
0.725944618	0.7254435	0.986100714
0.725947365	0.7254435	0.986067939
0.725840137	0.7254435	0.986035156
0.725842628	0.7254435	0.986002366
0.725845124	0.7254435	0.985969559
0.725405577	0.7254435	0.985936744
0.725407006	0.7254435	0.985903922
0.725408439	0.7254435	0.985871049
0.725581674	0.7254435	0.98583817
0.725583532	0.7254435	0.985805281
0.725412758	0.7254435	0.985772402
0.725414204	0.7254435	0.985739515
0.725550835	0.7254435	0.985706605
0.725552618	0.7254435	0.985673687
0.725554405	0.7254435	0.985640775
0.725556197	0.7254435	0.985607856
0.725557993	0.7254435	0.985574929

0.725598561	0.7254335	0.985541995
0.725456113	0.7254335	0.985509055
0.725457665	0.7254335	0.985476111
0.725604272	0.7254335	0.985443145
0.725835265	0.7254335	0.985410171
0.726069018	0.7254335	0.985377205
0.726072071	0.7254335	0.985344253
0.725791699	0.7254335	0.985311316
0.725794071	0.7254335	0.985278372
0.725796448	0.7254335	0.985245393
0.726372089	0.7254335	0.985212407
0.727858994	0.7254335	0.985179415
0.728198525	0.7254335	0.985146472
0.732545378	0.7254335	0.98511367
0.728215224	0.7254335	0.985080894
0.725810836	0.7254335	0.98504854
0.725415707	0.7254335	0.985015753
0.725417161	0.7254335	0.984982723
0.725418619	0.7254335	0.984949649
0.725361703	0.7254335	0.984916568
0.725285719	0.7254335	0.98488348
0.725286858	0.7254335	0.984850379
0.725287999	0.7254335	0.984817261
0.725289143	0.7254335	0.984784134
0.725340984	0.7254335	0.984750998
0.725342257	0.7254335	0.984717854
0.725399961	0.7254335	0.984684704
0.725497762	0.7254335	0.984651543
0.72565353	0.7254335	0.984618376
0.725655564	0.7254335	0.984585206
0.725657602	0.7254335	0.984552039
0.725659646	0.7254335	0.984518859
0.725661694	0.7254335	0.984485667
0.725507743	0.7254335	0.984452461
0.725545596	0.7254335	0.984419241
0.725511103	0.7254335	0.984385993
0.725512789	0.7254335	0.984352735
0.725514479	0.7254335	0.984319459
0.725516173	0.7254335	0.984286169
0.725554443	0.7254335	0.984252866
0.725556234	0.7254335	0.984219549
0.72555803	0.7254335	0.984186224
0.725559831	0.7254335	0.984152886
0.725561635	0.7254335	0.984119536

0.72552638	0.7254435	0.984086173
0.725528104	0.7254435	0.984052799
0.725529831	0.7254435	0.98401941
0.725531563	0.7254435	0.983986011
0.725533298	0.7254435	0.983952602
0.725612043	0.7254435	0.983919182
0.725845333	0.7254435	0.983885751
0.726368475	0.7254435	0.983852318
0.740629196	0.7254435	0.983818896
0.740662427	0.7254435	0.983785513
0.740695701	0.7254435	0.983753522
0.740729016	0.7254435	0.983721522
0.746037711	0.7254435	0.983689511
0.755643258	0.7254435	0.983657489
0.755679592	0.7254435	0.983625977
0.732621459	0.7254435	0.983595397
0.726764165	0.7254435	0.983564806
0.725594933	0.7254435	0.983531932
0.725521298	0.7254435	0.983498469
0.725523009	0.7254435	0.983464878
0.725641527	0.7254435	0.983431267
0.725780061	0.7254435	0.983397643
0.725782404	0.7254435	0.983364018
0.725784753	0.7254435	0.983330392
0.725787108	0.7254435	0.983296752
0.725789468	0.7254435	0.983263098
0.725791835	0.7254435	0.983229432
0.725794207	0.7254435	0.983195754
0.726217569	0.7254435	0.983162063
0.726220988	0.7254435	0.983128361
0.726298253	0.7254435	0.983094687
0.726301871	0.7254435	0.983061001
0.726305499	0.7254435	0.983027307
0.725860405	0.7254435	0.9829936
0.725414302	0.7254435	0.982959879
0.72558926	0.7254435	0.9829261
0.725868044	0.7254435	0.982892261
0.726402983	0.7254435	0.982858425
0.726406861	0.7254435	0.982824601
0.727220755	0.7254435	0.982790813
0.72906938	0.7254435	0.98275701
0.730336122	0.7254435	0.982723272
0.727950819	0.7254435	0.982689701
0.727958543	0.7254435	0.98265624

0.727966286	0.7254435	0.98262253
0.727974048	0.7254435	0.982588807
0.727671247	0.7254435	0.982555071
0.727678275	0.7254435	0.982521321
0.726717183	0.7254435	0.982487527
0.725958638	0.7254435	0.98245372
0.725617945	0.7254435	0.982419807
0.725580013	0.7254435	0.982385806
0.725543885	0.7254435	0.982351759
0.725545651	0.7254435	0.982317696
0.725476737	0.7254435	0.982283615
0.725549196	0.7254435	0.982249522
0.725550974	0.7254435	0.982215409
0.725481556	0.7254435	0.982181291
0.725450102	0.7254435	0.98214716
0.72545164	0.7254435	0.982113008
0.72548641	0.7254435	0.982078839
0.725454726	0.7254435	0.982044654
0.725456275	0.7254435	0.982010456
0.725491299	0.7254435	0.98197624
0.725565362	0.7254435	0.981942008
0.72556718	0.7254435	0.981907765
0.725569003	0.7254435	0.981873515
0.725789518	0.7254435	0.981839249
0.726917671	0.7254435	0.981804968
0.728727847	0.7254435	0.981770694
0.728737498	0.7254435	0.981736515
0.728747173	0.7254435	0.981702502
0.728756872	0.7254435	0.981668476
0.728766594	0.7254435	0.981634436
0.726948785	0.7254435	0.981600381
0.725808612	0.7254435	0.981566311
0.725670079	0.7254435	0.981532047
0.725672153	0.7254435	0.981497655
0.725674232	0.7254435	0.981463236
0.725676316	0.7254435	0.981428801
0.725678405	0.7254435	0.981394351
0.725596883	0.7254435	0.981359885
0.725682599	0.7254435	0.981325403
0.725684703	0.7254435	0.981290897
0.725780195	0.7254435	0.981256383
0.72626992	0.7254435	0.981221853
0.728309459	0.72580972	0.981187316
0.728318073	0.72618444	0.981152812

0.727822837	0.72655916	0.981118493
0.727830242	0.72693388	0.981084159
0.727837666	0.7273086	0.98104976
0.727845108	0.72768332	0.981015345
0.731747087	0.72805804	0.980980914
0.727860047	0.72843276	0.980946468
0.727867545	0.72880748	0.980912389
0.727875062	0.7291822	0.98087791
0.727311048	0.72955692	0.980843415
0.728052902	0.72993164	0.980808906
0.72806088	0.73030636	0.980774325
0.728068877	0.73068108	0.980739801
0.728076894	0.7310558	0.980705262
0.731211152	0.73143052	0.980670707
0.729770853	0.73180524	0.980636138
0.731935304	0.73217996	0.980601862
0.731952653	0.73255468	0.98056743
0.731970041	0.7329294	0.980533194
0.731987469	0.73330412	0.980498943
0.732004937	0.73367884	0.980464676
0.732022445	0.73405356	0.980430393
0.728326799	0.73442828	0.980396095
0.728335457	0.7348030	0.980361781
0.726622444	0.73517772	0.980327085
0.726626865	0.73555244	0.980292372
0.726151263	0.73592716	0.980257472
0.726087836	0.73630188	0.980222554
0.725803978	0.7366766	0.980187571
0.726094043	0.73705132	0.980152566
0.726309477	0.73742604	0.980117515
0.726653621	0.73780076	0.980082477
0.726316778	0.73817548	0.980047442
0.726862236	0.7385502	0.980012425
0.728826158	0.73892492	0.979977359
0.72687228	0.73929964	0.97994233
0.726877321	0.73967436	0.979907478
0.728855913	0.74004908	0.979872418
0.730337393	0.7404238	0.979837342
0.730643366	0.74079852	0.979802443
0.739720081	0.74117324	0.979767706
0.739752108	0.74154796	0.979733014
0.73978418	0.74192268	0.979699231
0.730700841	0.7422974	0.979665466
0.730715295	0.74267212	0.979631719

0.734161558	0.74304684	0.979597092
0.734183806	0.74342156	0.97956248
0.737395501	0.74379628	0.979528221
0.745928531	0.744171	0.97949398
0.739340397	0.74454572	0.979460069
0.737480907	0.74492044	0.979427012
0.731466397	0.74529516	0.979393322
0.737538087	0.74566988	0.979359463
0.739466626	0.7460446	0.979325026
0.740866528	0.74641932	0.979291203
0.740900054	0.74679404	0.979257585
0.740933622	0.74716876	0.97922412
0.747874119	0.74754348	0.979190673
0.74791259	0.7479182	0.979157243
0.747951068	0.74829292	0.97912451
0.766494358	0.74866764	0.979091795
0.766521033	0.74904236	0.979059098
0.776640111	0.74941708	0.979028242
0.777216666	0.7497918	0.978997402
0.775428669	0.75016652	0.978967572
0.769756332	0.75054124	0.978937811
0.769779484	0.75091596	0.978907887
0.75911538	0.75129068	0.978877418
0.752333566	0.7516654	0.978846965
0.759182695	0.75204012	0.978815476
0.752409657	0.75241484	0.978783332
0.755576014	0.75278956	0.978751877
0.755612393	0.75316428	0.978719768
0.755648748	0.753539	0.978687984
0.755685078	0.75391372	0.978656218
0.762733043	0.75428844	0.978624469
0.760794172	0.75466316	0.978592739
0.760826362	0.75503788	0.978561717
0.770989436	0.7554126	0.978530518
0.760890655	0.75578732	0.978499337
0.760922758	0.75616204	0.97846917
0.759583295	0.75653676	0.978438021
0.752067225	0.75691148	0.978406889
0.752105369	0.7572862	0.978375639
0.752143501	0.75766092	0.978343662
0.752181619	0.75803564	0.978311702
0.763690803	0.75841036	0.97827976
0.774425783	0.75878508	0.978247835
0.774442968	0.7591598	0.978217056

0.780785844	0.75953452	0.978187348
0.780793301	0.75990924	0.978157654
0.780800747	0.76028396	0.978128599
0.782740735	0.76065868	0.978099558
0.782746987	0.7610334	0.978070533
0.77857678	0.76140812	0.978041713
0.778120656	0.76178284	0.978012909
0.778132156	0.76215756	0.977983708
0.77764564	0.76253228	0.977954478
0.766389339	0.762907	0.977925263
0.766416121	0.76328172	0.977896015
0.777152205	0.76365644	0.977865673
0.77716526	0.76403116	0.977835348
0.766496304	0.76440588	0.977806096
0.763572959	0.7647806	0.977776859
0.767101912	0.76515532	0.977746588
0.777217273	0.76553004	0.977716046
0.777230225	0.76590476	0.977685866
0.767179968	0.76627948	0.977656698
0.767205933	0.7666542	0.977627547
0.764959462	0.76702892	0.977597421
0.764987685	0.76740364	0.977567315
0.767820682	0.76777836	0.977537003
0.76784597	0.76815308	0.977506711
0.76787123	0.7685278	0.977476713
0.763896776	0.76890252	0.977446734
0.761369457	0.76927724	0.977416772
0.761401121	0.76965196	0.977386434
0.761432756	0.77002668	0.977355863
0.764013672	0.7704014	0.97732531
0.771418726	0.77077612	0.977294776
0.771439904	0.77115084	0.977264513
0.761559003	0.77152556	0.977234993
0.761590491	0.77190028	0.977205492
0.760956334	0.772275	0.977175033
0.758914685	0.77264972	0.977144593
0.758948528	0.77302444	0.977114108
0.758982341	0.77339916	0.977083436
0.763661923	0.77377388	0.977052784
0.763691417	0.7741486	0.977022149
0.758369692	0.77452332	0.97699199
0.756941341	0.77489804	0.976961849
0.75697676	0.77527276	0.9769312
0.763809111	0.77564748	0.976900426

0.76837015	0.7760222	0.97686967
0.771733662	0.77639692	0.976839603
0.777596634	0.77677164	0.976809999
0.77177521	0.77714636	0.97678074
0.771795945	0.77752108	0.976752072
0.771816655	0.7778958	0.976722845
0.777645895	0.77827052	0.976693633
0.777658161	0.77864524	0.976664436
0.780542653	0.77901996	0.976635827
0.783327881	0.77939468	0.976607232
0.782797362	0.7797694	0.976578933
0.781328134	0.78014412	0.976550921
0.784080508	0.78051884	0.97652287
0.786762751	0.78089356	0.976494688
0.791297482	0.78126828	0.976466792
0.791321405	0.781643	0.976439173
0.790385895	0.78201772	0.976412015
0.790407683	0.78239244	0.976384874
0.790429523	0.78276716	0.976357655
0.788418	0.78314188	0.976330454
0.788435045	0.7835166	0.976303271
0.788452132	0.78389132	0.976275905
0.790064865	0.78426604	0.976248557
0.785036934	0.78464076	0.976221228
0.785046115	0.78501548	0.976194074
0.782891615	0.7853902	0.976166442
0.783582062	0.78576492	0.976138828
0.785073768	0.78613964	0.976111018
0.783595727	0.78651436	0.976083294
0.784209212	0.78688908	0.976055733
0.784216889	0.7872638	0.976028042
0.784224578	0.78763852	0.976000427
0.782016632	0.78801324	0.975972828
0.778892791	0.78838796	0.975945245
0.775398803	0.78876268	0.975917459
0.775414567	0.7891374	0.975889381
0.775430308	0.78951212	0.975860975
0.775446024	0.78988684	0.975832586
0.775461718	0.79026156	0.975804214
0.774444578	0.79063628	0.97577586
0.774461736	0.791011	0.975747524
0.775508657	0.79138572	0.975719104
0.775524257	0.79176044	0.975690702
0.781403506	0.79213516	0.975662419

0.78301185	0.79250988	0.975634154
0.785243534	0.7928846	0.975606486
0.785253137	0.79325932	0.975578994
0.783030939	0.79363404	0.975551736
0.783037309	0.79400876	0.975524497
0.787252301	0.79438348	0.975497055
0.787266512	0.7947582	0.975469628
0.792220096	0.79513292	0.975442632
0.797487869	0.79550764	0.975415654
0.811662816	0.79588236	0.975389179
0.811712968	0.79625708	0.975363239
0.799196158	0.7966318	0.97533871
0.799235601	0.79700652	0.9753142
0.799275105	0.79738124	0.975288474
0.794832825	0.79775596	0.975262768
0.799354299	0.79813068	0.975237082
0.794896088	0.7985054	0.975210976
0.787424743	0.79888012	0.975185332
0.787439372	0.79925484	0.975159267
0.787454035	0.79962956	0.975132483
0.787468735	0.80000428	0.975105718
0.78416356	0.8003790	0.975078973
0.784171165	0.80075372	0.975052247
0.784178782	0.80112844	0.975025213
0.789303884	0.80150316	0.974998198
0.785272254	0.80187788	0.974971202
0.793915636	0.8022526	0.974944729
0.797416177	0.80262732	0.974917878
0.797452658	0.80300204	0.974891896
0.789400334	0.80337676	0.974866276
0.789419764	0.80375148	0.974840676
0.789439241	0.8041262	0.974814302
0.800904542	0.80450092	0.974787947
0.827407558	0.80487564	0.974761614
0.815023335	0.80525036	0.97473643
0.815073314	0.80562508	0.974713874
0.815123281	0.8059998	0.974690119
0.836543559	0.80637452	0.974666388
0.811013941	0.80674924	0.974642683
0.836589385	0.80712396	0.974621107
0.836612232	0.80749868	0.974597039
0.836635034	0.8078734	0.974575508
0.830789696	0.80824812	0.974554
0.816514512	0.80862284	0.974532514

0.81656401	0.80899756	0.974510471
0.816613488	0.80937228	0.974487043
0.816662946	0.809747	0.974463637
0.796630578	0.81012172	0.974440256
0.816761799	0.81049644	0.9744169
0.816811194	0.81087116	0.974391591
0.816860567	0.81124588	0.974368283
0.822871129	0.8116206	0.974344999
0.816959249	0.81199532	0.974321739
0.800904979	0.81237004	0.974299087
0.827407966	0.81274476	0.974275872
0.827447025	0.81311948	0.974251094
0.801031038	0.8134942	0.974228945
0.820246334	0.81386892	0.974206819
0.814123003	0.81424364	0.974182111
0.796318227	0.81461836	0.974159316
0.796352715	0.81499308	0.974135938
0.789616869	0.8153678	0.974110827
0.799555233	0.81574252	0.97408574
0.812267378	0.81611724	0.974060011
0.812317593	0.81649196	0.974035282
0.812367812	0.81686668	0.974011824
0.812418033	0.8172414	0.973988392
0.811415041	0.81761612	0.973964985
0.812518484	0.81799084	0.973941603
0.815722352	0.81836556	0.973918143
0.815772136	0.81874028	0.973894813
0.828064154	0.819115	0.973871817
0.828102235	0.81948972	0.973848846
0.828140257	0.81986444	0.973827105
0.814924385	0.82023916	0.973805386
0.823933124	0.82061388	0.97378369
0.832554759	0.8209886	0.973760712
0.832585288	0.82136332	0.97373864
0.832615762	0.82173804	0.973717438
0.836544037	0.82211276	0.973696257
0.83607608	0.82248748	0.973675099
0.836099926	0.8228622	0.973654347
0.816368223	0.82323692	0.973633568
0.805033475	0.82361164	0.97361281
0.836171184	0.82398636	0.97359013
0.840923355	0.82436108	0.973566355
0.836703646	0.8247358	0.973545667
0.840953013	0.82511052	0.973525469

0.843169898	0.82548524	0.973504876
0.847606094	0.82585996	0.973484722
0.850168254	0.82623468	0.973464808
0.850180103	0.8266094	0.973445353
0.850191959	0.82698412	0.973426171
0.851496731	0.82735884	0.973407013
0.85098015	0.82773356	0.973387878
0.849023025	0.82810828	0.973368891
0.851268194	0.828483	0.973349873
0.849045262	0.82885772	0.973330683
0.847700167	0.82923244	0.973311733
0.843051632	0.82960716	0.973292583
0.843063528	0.82998188	0.973273319
0.832677648	0.8303566	0.973253616
0.832707952	0.83073132	0.973233935
0.832738201	0.83110604	0.973213253
0.832768395	0.83148076	0.973192595
0.832798533	0.83185548	0.97317196
0.826977854	0.8322302	0.973151347
0.827017536	0.83260492	0.973130758
0.836242886	0.83297964	0.973109615
0.843170021	0.83335436	0.973088497
0.84318179	0.83372908	0.973068306
0.843193547	0.8341038	0.973048817
0.843205292	0.83447852	0.973029349
0.846557595	0.83485324	0.9730099
0.847429689	0.83522796	0.972990474
0.8512434	0.83560268	0.972971397
0.851256023	0.8359774	0.972952428
0.847460833	0.83635212	0.972933855
0.847471221	0.83672684	0.972915305
0.845333886	0.83710156	0.972896401
0.837884194	0.83747628	0.972877521
0.831322349	0.837851	0.972858451
0.834479483	0.83822572	0.972838669
0.834506411	0.83860044	0.972818262
0.830003136	0.83897516	0.972798187
0.830038127	0.83934988	0.972778136
0.836172294	0.8397246	0.972757662
0.836195953	0.84009932	0.972737212
0.836219565	0.84047404	0.972717387
0.832888927	0.84084876	0.972697586
0.84341465	0.84122348	0.972677807
0.847826475	0.8415982	0.97265772

0.847836996	0.84197292	0.972638692
0.845262066	0.84234764	0.972620119
0.84527239	0.84272236	0.972601568
0.845282709	0.84309708	0.972582784
0.845293026	0.8434718	0.972564021
0.849023546	0.84384652	0.972545281
0.849034662	0.84422124	0.972526562
0.852377129	0.84459596	0.97250823
0.852966579	0.84497068	0.97248992
0.852980533	0.8453454	0.972471959
0.854206875	0.8453454	0.972454077
0.856200251	0.8453454	0.972436216
0.856216769	0.8453454	0.972418496
0.854251724	0.8453454	0.972400993
0.853050464	0.8453454	0.972383512
0.85220319	0.8453454	0.97236586
0.849621496	0.8453454	0.972348112
0.847140931	0.8453454	0.972330302
0.839619425	0.8453454	0.97231226
0.847161525	0.8453454	0.972293996
0.847171827	0.8453454	0.972275014
0.84718213	0.8453454	0.972256797
0.847192437	0.8453454	0.972238603
0.853779116	0.8453454	0.972220431
0.854416695	0.8453454	0.972202282
0.8544318	0.8453454	0.972184803
0.854446917	0.8453454	0.972167406
0.854462046	0.8453454	0.972150029
0.85948122	0.8453454	0.972132675
0.855139438	0.8453454	0.972115344
0.855155118	0.8453454	0.97209853
0.853590554	0.8453454	0.97208131
0.851068052	0.8453454	0.972064113
0.84910161	0.8453454	0.972046783
0.845385845	0.8453454	0.972029226
0.845611457	0.8453454	0.972011497
0.84562169	0.8453454	0.971993426
0.845631922	0.8453454	0.9719754
0.845642152	0.8453454	0.971957397
0.84565238	0.8453454	0.971939417
0.845662606	0.8453454	0.97192146
0.84567283	0.8453454	0.971903526
0.845683053	0.8453454	0.971885584
0.845693274	0.8453454	0.971867633

0.845703492	0.8453454	0.971849672
0.848770252	0.8453454	0.971831703
0.848781225	0.8453454	0.971813724
0.848792203	0.8453454	0.971796037
0.848803188	0.8453454	0.971778342
0.846395585	0.8453454	0.971760638
0.844678391	0.8453454	0.971742925
0.844688959	0.8453454	0.971724966
0.844699522	0.8453454	0.971706828
0.84471008	0.8453454	0.971688681
0.844720632	0.8453454	0.971670527
0.843823135	0.8453454	0.971652363
0.840895153	0.8453454	0.971634192
0.84091004	0.8453454	0.971615923
0.840924902	0.8453454	0.971597355
0.844098845	0.8453454	0.971578779
0.846293913	0.8453454	0.971560194
0.842402839	0.8453454	0.971541911
0.840004924	0.8453454	0.971523833
0.838929353	0.8453454	0.971505363
0.833621025	0.8453454	0.971486645
0.834236657	0.8453454	0.971467813
0.834264048	0.8453454	0.971448448
0.839002562	0.8453454	0.971429136
0.83902078	0.8453454	0.971409817
0.839038963	0.8453454	0.971390957
0.839057112	0.8453454	0.971372089
0.839447397	0.8453454	0.971353214
0.839464794	0.8453454	0.971334333
0.843789451	0.8453454	0.971315481
0.84380063	0.8453454	0.971296623
0.844044165	0.8453454	0.971278182
0.84405514	0.8453454	0.971259734
0.844066107	0.8453454	0.9712413
0.839568967	0.8453454	0.971222857
0.839586144	0.8453454	0.971204405
0.838047679	0.8453454	0.9711855
0.833507319	0.8453454	0.971166587
0.838087687	0.8453454	0.971147512
0.833564801	0.8453454	0.97112798
0.837257931	0.8453454	0.971108889
0.837279478	0.8453454	0.971089343
0.84296936	0.8453454	0.97107015
0.842981348	0.8453454	0.971050948

0.846793292	0.8453454	0.971032297
0.850229249	0.8453454	0.971013637
0.857180166	0.8453454	0.970995342
0.864257073	0.8453454	0.970977377
0.864279457	0.8453454	0.970960086
0.864301856	0.8453454	0.970943484
0.86432427	0.8453454	0.970926875
0.855857628	0.8453454	0.97091026
0.843575713	0.8453454	0.970893639
0.848422937	0.8453454	0.970876177
0.848433727	0.8453454	0.970857498
0.848444522	0.8453454	0.970839289
0.848455323	0.8453454	0.970821073
0.846283949	0.8453454	0.970802849
0.840954863	0.8453454	0.970784617
0.840969653	0.8453454	0.970766162
0.843183138	0.8453454	0.970747172
0.846324655	0.8453454	0.970728175
0.852870298	0.8453454	0.970709387
0.862770936	0.8453454	0.970690898
0.86279232	0.8453454	0.970673044
0.851782377	0.8453454	0.970656156
0.845304145	0.8453454	0.97063926
0.844657843	0.8453454	0.970621271
0.844668423	0.8453454	0.970602635
0.844678997	0.8453454	0.970583927
0.844689565	0.8453454	0.970565211
0.844700127	0.8453454	0.970546486
0.844033697	0.8453454	0.970527753
0.84404468	0.8453454	0.970509012
0.841775254	0.8453454	0.970490197
0.841203094	0.8453454	0.970471374
0.836175007	0.8453454	0.97045232
0.84123186	0.8453454	0.970433202
0.843392897	0.8453454	0.970413581
0.84340444	0.8453454	0.970394451
0.84389025	0.8453454	0.970375527
0.843901346	0.8453454	0.970356597
0.845252655	0.8453454	0.970337707
0.843923511	0.8453454	0.970318812
0.84393458	0.8453454	0.97030004
0.84394564	0.8453454	0.970281127
0.847660007	0.8453454	0.970262207
0.851257601	0.8453454	0.970243278

0.852940302	0.8453454	0.970224706
0.854799386	0.8453454	0.970206478
0.856855294	0.8453454	0.970188407
0.861170304	0.8453454	0.97017051
0.861190567	0.8453454	0.970152806
0.861210845	0.8453454	0.970135517
0.861231137	0.8453454	0.970118221
0.861251444	0.8453454	0.970100918
0.858826281	0.8453454	0.970083607
0.852486904	0.8453454	0.970066287
0.84584682	0.8453454	0.970048719
0.843632981	0.8453454	0.970030517
0.839973156	0.8453454	0.970011652
0.83998961	0.8453454	0.969992561
0.837682005	0.8453454	0.969973099
0.837702731	0.8453454	0.969953629
0.839689529	0.8453454	0.969933921
0.841332353	0.8453454	0.969914206
0.841639151	0.8453454	0.969894676
0.841360717	0.8453454	0.969875299
0.842224715	0.8453454	0.969855941
0.842237619	0.8453454	0.969836545
0.846824554	0.8453454	0.969817225
0.85156344	0.8453454	0.969797894
0.851576304	0.8453454	0.969779004
0.851589177	0.8453454	0.969760569
0.851602061	0.8453454	0.969742124
0.851082302	0.8453454	0.969723668
0.847535303	0.8453454	0.969705202
0.84912522	0.8453454	0.969686675
0.849136396	0.8453454	0.969667789
0.845847192	0.8453454	0.96964905
0.845857389	0.8453454	0.969630303
0.845867584	0.8453454	0.969611223
0.844121844	0.8453454	0.969592136
0.844132762	0.8453454	0.96957304
0.844143671	0.8453454	0.969553763
0.841612134	0.8453454	0.969534479
0.845704397	0.8453454	0.969515185
0.84809213	0.8453454	0.969495632
0.846579734	0.8453454	0.969476472
0.846589928	0.8453454	0.969457536
0.84138914	0.8453454	0.969438444
0.841403246	0.8453454	0.969419344

0.84445643	0.8453454	0.969399723
0.855157079	0.8453454	0.969380094
0.855172772	0.8453454	0.969360758
0.855188478	0.8453454	0.969342465
0.847965009	0.8453454	0.969324163
0.8456025	0.8453454	0.969305853
0.845612736	0.8453454	0.969286822
0.848673133	0.8453454	0.969267548
0.848684053	0.8453454	0.969248264
0.848694979	0.8453454	0.969229271
0.845653658	0.8453454	0.969210269
0.845663884	0.8453454	0.969191258
0.844360275	0.8453454	0.969171939
0.841304569	0.8453454	0.969152612
0.846335786	0.8453454	0.969133148
0.846132372	0.8453454	0.969113373
0.842467144	0.8453454	0.969094085
0.84074683	0.8453454	0.969074766
0.839757536	0.8453454	0.969055076
0.839774373	0.8453454	0.969035205
0.840137162	0.8453454	0.969015226
0.840153329	0.8453454	0.968995237
0.842801488	0.8453454	0.968975273
0.844921341	0.8453454	0.9689553
0.842825834	0.8453454	0.968935576
0.842837985	0.8453454	0.968916048
0.844056683	0.8453454	0.968896301
0.849600522	0.8453454	0.968876543
0.849611992	0.8453454	0.968856892
0.849623469	0.8453454	0.968837774
0.845212286	0.8453454	0.968818645
0.845222625	0.8453454	0.968799504
0.845232961	0.8453454	0.968779915
0.846957888	0.8453454	0.968760314
0.846968142	0.8453454	0.968740702
0.848521371	0.8453454	0.968721246
0.845919025	0.8453454	0.968701778
0.842467603	0.8453454	0.968682449
0.842480191	0.8453454	0.968662851
0.844198612	0.8453454	0.968642899
0.848575626	0.8453454	0.968622937
0.845325836	0.8453454	0.968603131
0.84170873	0.8453454	0.968583745
0.841722357	0.8453454	0.968564028

0.841735963	0.8453454	0.968543944
0.84174955	0.8453454	0.968523851
0.843090001	0.8453454	0.968503749
0.843101856	0.8453454	0.968483638
0.843113698	0.8453454	0.968463649
0.843125528	0.8453454	0.968443649
0.841529884	0.8453454	0.968423639
0.837621541	0.8453454	0.968403619
0.837642384	0.8453454	0.96838343
0.837663187	0.8453454	0.968362842
0.836295255	0.8453454	0.968342245
0.835295966	0.8453454	0.968321639
0.835321325	0.8453454	0.968300886
0.836365376	0.8453454	0.968280023
0.836388658	0.8453454	0.968259153
0.836411896	0.8453454	0.968238374
0.837807688	0.8453454	0.968217586
0.838249164	0.8453454	0.96819679
0.843030967	0.8453454	0.968176121
0.838288402	0.8453454	0.968155485
0.838307965	0.8453454	0.968135308
0.838327491	0.8453454	0.968114655
0.839858893	0.8453454	0.968093992
0.839875549	0.8453454	0.968073322
0.844952899	0.8453454	0.968052793
0.851107839	0.8453454	0.968032256
0.844973771	0.8453454	0.96801221
0.840281986	0.8453454	0.96799276
0.840297902	0.8453454	0.967972697
0.841262176	0.8453454	0.967952162
0.841276478	0.8453454	0.967931622
0.844584954	0.8453454	0.967911168
0.844595573	0.8453454	0.967890707
0.844606186	0.8453454	0.967870561
0.840392822	0.8453454	0.967850406
0.839362949	0.8453454	0.96783024
0.839380501	0.8453454	0.967809648
0.839398021	0.8453454	0.967788943
0.839042085	0.8453454	0.967768227
0.838672686	0.8453454	0.967747501
0.838691521	0.8453454	0.967726727
0.838710321	0.8453454	0.967705905
0.839114458	0.8453454	0.967685072
0.839132467	0.8453454	0.967664229

0.840549279	0.8453454	0.967643413
0.843348063	0.8453454	0.967622587
0.846255065	0.8453454	0.967601889
0.84626524	0.8453454	0.967581454
0.846489086	0.8453454	0.967561291
0.846499272	0.8453454	0.967541116
0.846509459	0.8453454	0.967520949
0.848717873	0.8453454	0.967500771
0.847174188	0.8453454	0.967480579
0.847184492	0.8453454	0.967460592
0.847194799	0.8453454	0.967440442
0.843463417	0.845678484	0.967420281
0.843474891	0.846011568	0.967400109
0.843486354	0.846344652	0.967379557
0.841074877	0.846677736	0.967358995
0.843980435	0.84701082	0.967338421
0.853855576	0.847343904	0.967317599
0.844901405	0.847676988	0.967297051
0.849544354	0.848010072	0.967277465
0.846214734	0.848343156	0.967256986
0.846224909	0.84867624	0.967236952
0.846876889	0.849009324	0.967216579
0.84624526	0.849342408	0.967196194
0.8491265	0.849675492	0.967175862
0.849137677	0.850008576	0.967155458
0.84914886	0.85034166	0.967135327
0.846285963	0.850674744	0.967115186
0.846296139	0.851007828	0.967095037
0.842923784	0.851340912	0.967074595
0.842680174	0.851673996	0.967054142
0.842692499	0.85200708	0.967033344
0.842443235	0.852340164	0.967012511
0.844166431	0.852673248	0.966991669
0.844177317	0.853006332	0.96697079
0.844188195	0.853339416	0.96695007
0.844199065	0.8536725	0.966929338
0.845099127	0.854005584	0.966908595
0.845326533	0.854338668	0.96688784
0.845552465	0.854671752	0.966867161
0.849306131	0.855004836	0.966846493
0.845572955	0.85533792	0.966825835
0.845797729	0.855671004	0.966805534
0.851889497	0.856004088	0.966784853
0.856237434	0.856337172	0.96676418

0.856253981	0.856670256	0.966744095
0.856270542	0.85700334	0.966724425
0.856287115	0.857336424	0.966704744
0.855618951	0.857669508	0.966685052
0.860101745	0.858002592	0.966665347
0.855651083	0.858335676	0.966645563
0.860140739	0.85866876	0.966626207
0.863183479	0.859001844	0.966606399
0.862308784	0.859334928	0.96658702
0.870229768	0.859668012	0.966567926
0.882925717	0.860001096	0.966548731
0.882306464	0.86033418	0.966530301
0.870307723	0.860667264	0.966513106
0.874302769	0.861000348	0.966495835
0.869279953	0.861333432	0.966477367
0.869305401	0.861666516	0.966459279
0.874386812	0.8619996	0.966440684
0.860356229	0.862332684	0.96642208
0.866786369	0.862665768	0.966403964
0.855877432	0.862998852	0.966384455
0.851361118	0.863331936	0.966365568
0.851373829	0.86366502	0.966345595
0.855926261	0.863998104	0.966325168
0.852503169	0.864331188	0.966304731
0.857011997	0.864664272	0.966284731
0.85702915	0.864997356	0.966264382
0.857046316	0.86533044	0.966244465
0.857063495	0.865663524	0.966224537
0.852859464	0.865996608	0.966204597
0.852873333	0.866329692	0.966184646
0.855715667	0.866662776	0.966164268
0.855731803	0.86699586	0.966143879
0.855747951	0.867328944	0.966123756
0.855096547	0.867662028	0.966103621
0.855112192	0.867995112	0.966083474
0.858316243	0.868328196	0.966063273
0.85514352	0.86866128	0.966043084
0.85483303	0.868994364	0.966023221
0.858370753	0.869327448	0.966003057
0.85838895	0.869660532	0.965982872
0.86166417	0.869993616	0.965963045
0.854894847	0.8703267	0.965943229
0.854910332	0.870659784	0.965923746
0.85492583	0.870992868	0.965903606

0.859658505	0.871325952	0.965883479
0.859278401	0.871659036	0.965863363
0.856320646	0.87199212	0.965843726
0.871226486	0.872325204	0.965824062
0.856353884	0.872658288	0.965804118
0.878368119	0.872991372	0.965785653
0.878397793	0.873324456	0.965765733
0.878427477	0.87365754	0.965747992
0.878457172	0.873990624	0.965730264
0.878486877	0.874323708	0.965712546
0.861093287	0.874656792	0.965694842
0.877924643	0.874989876	0.965677149
0.881108765	0.87532296	0.965657752
0.881139357	0.875656044	0.965640023
0.913890607	0.875989128	0.965622617
0.881200568	0.876322212	0.965605225
0.884491388	0.876655296	0.965591067
0.884522875	0.87698838	0.965573698
0.887220645	0.877321464	0.965556663
0.902156306	0.877654548	0.965539642
0.902187952	0.877987632	0.965522896
0.89342516	0.878320716	0.965507631
0.893457691	0.8786538	0.965492377
0.893490223	0.878986884	0.965476269
0.893522756	0.879319968	0.965460171
0.893555288	0.879653052	0.965444085
0.880257457	0.879986136	0.965428012
0.880287782	0.88031922	0.96541195
0.880318117	0.880652304	0.96539459
0.873637179	0.880985388	0.965377243
0.87974455	0.881318472	0.965359909
0.879774706	0.881651556	0.965341927
0.879804872	0.88198464	0.965324557
0.876120834	0.882317724	0.965307199
0.876149628	0.882650808	0.965289855
0.879895431	0.882983892	0.965272157
0.876207251	0.883316976	0.965254472
0.876236081	0.88365006	0.965237165
0.876264923	0.883983144	0.965219502
0.891964593	0.884316228	0.965201849
0.887926047	0.884649312	0.965184206
0.891347833	0.884982396	0.965168116
0.883333324	0.88531548	0.965151638
0.91691127	0.885648564	0.965135503

0.90360364	0.885981648	0.965118587
0.885406476	0.886314732	0.965104985
0.885438159	0.886647816	0.965090078
0.885469848	0.8869809	0.965073386
0.885501544	0.887313984	0.965056706
0.880318433	0.887647068	0.965040039
0.880348777	0.887980152	0.965023383
0.880379132	0.888313236	0.965006226
0.88562839	0.88864632	0.964989081
0.885660118	0.888979404	0.964971948
0.88972867	0.889312488	0.96495534
0.884397625	0.889645572	0.964938743
0.884429091	0.889978656	0.964922555
0.895278725	0.89031174	0.964905849
0.881232624	0.890644824	0.964889155
0.881263253	0.890977908	0.964873536
0.881293892	0.891310992	0.964856542
0.88132454	0.891644076	0.964839559
0.872244097	0.89197716	0.964822589
0.867930128	0.892310244	0.964805632
0.867954801	0.892643328	0.96478779
0.861358081	0.892976412	0.964769535
0.864062883	0.893309496	0.964751293
0.863621658	0.89364258	0.964732413
0.86364362	0.893975664	0.964713811
0.863665597	0.894308748	0.964695176
0.871895061	0.894641832	0.964676555
0.870806829	0.894974916	0.964657946
0.87251489	0.895308	0.964640158
0.884965906	0.895641084	0.964622273
0.888344272	0.895974168	0.964604567
0.888376448	0.896307252	0.964588097
0.879234397	0.896640336	0.964571969
0.888440812	0.89697342	0.964555854
0.913920418	0.897306504	0.964538848
0.904042493	0.897639588	0.96452276
0.919432734	0.897972672	0.964509192
0.919457225	0.898305756	0.964494661
0.919481701	0.89863884	0.964481656
0.919506163	0.898971924	0.964468664
0.919530609	0.899305008	0.964455683
0.919555041	0.899638092	0.964442715
0.922059187	0.899971176	0.964429759
0.922557654	0.90030426	0.964416813

0.922580179	0.900637344	0.964404122
0.93044637	0.900970428	0.964391488
0.922625186	0.901303512	0.964378865
0.922647668	0.901636596	0.964367023
0.919212552	0.90196968	0.964354417
0.908945539	0.902302764	0.96434182
0.915491857	0.902635848	0.964328891
0.919286376	0.902968932	0.964314958
0.918266536	0.903302016	0.964301678
0.918291715	0.9036351	0.964288781
0.907841236	0.903968184	0.964275792
0.8980005	0.904301268	0.964262814
0.907901336	0.904634352	0.964248816
0.909181701	0.904967436	0.964233858
0.899448919	0.90530052	0.964219882
0.909240823	0.905633604	0.964206041
0.899513162	0.905966688	0.964191249
0.895474399	0.906299772	0.96417743
0.899577387	0.906632856	0.964162661
0.890086051	0.90696594	0.964147503
0.899641593	0.907299024	0.964132758
0.899673689	0.907632108	0.964117086
0.89970578	0.907965192	0.964102365
0.891576129	0.908298276	0.964087655
0.904416754	0.90863136	0.964072956
0.891641082	0.908964444	0.964057467
0.893720155	0.909297528	0.964043254
0.902537752	0.909630612	0.964027794
0.902569316	0.909963696	0.964012549
0.922287259	0.91029678	0.963998184
0.902632424	0.910629864	0.96398383
0.922332649	0.910962948	0.963971427
0.907872387	0.911296032	0.963957097
0.907902432	0.911629116	0.963944716
0.918393202	0.9119622	0.96393092
0.930310622	0.912295284	0.963917136
0.930327733	0.912628368	0.963904394
0.930344832	0.912961452	0.963892834
0.930717297	0.913294536	0.963881284
0.926513647	0.91362762	0.963869747
0.932449077	0.913960704	0.963858255
0.928938453	0.914293788	0.963846358
0.928956552	0.914626872	0.963835054
0.928974639	0.914959956	0.963823414

0.928992712	0.91529304	0.963811783
0.924482701	0.915626124	0.963800162
0.923600444	0.915959208	0.963788551
0.913756694	0.916292292	0.963776505
0.899194109	0.916625376	0.963764379
0.909006228	0.91695846	0.963751294
0.909035861	0.917291544	0.963736785
0.907179926	0.917624628	0.963723254
0.907210207	0.917957712	0.963709735
0.909124692	0.918290796	0.963696044
0.909154281	0.91862388	0.963682366
0.909183857	0.918956964	0.963668887
0.909213423	0.919290048	0.963655422
0.912867601	0.919623132	0.96364197
0.921507313	0.919956216	0.963628531
0.921530518	0.9202893	0.96361546
0.921553707	0.920622384	0.96360325
0.922536405	0.920955468	0.963591052
0.925710673	0.921288552	0.963578866
0.925731064	0.921621636	0.963566786
0.915918072	0.92195472	0.963555028
0.925771804	0.922287804	0.963543281
0.933483862	0.922620888	0.963530574
0.933498722	0.922953972	0.963518845
0.933513571	0.923287056	0.963507883
0.933528409	0.92362014	0.963496929
0.929083541	0.923953224	0.963485984
0.929101536	0.924286308	0.963475046
0.920852676	0.924619392	0.963463678
0.917249462	0.924952476	0.963452321
0.91727522	0.92528556	0.96344016
0.917300964	0.925618644	0.963427654
0.912811842	0.925951728	0.963415162
0.915145194	0.926284812	0.963402684
0.909243285	0.926617896	0.963389776
0.909272828	0.92695098	0.963377108
0.915225819	0.927284064	0.96336387
0.916914243	0.927617148	0.963350647
0.926534405	0.927950232	0.963338022
0.926554219	0.928283316	0.963325574
0.930785285	0.9286164	0.963314084
0.926593806	0.928949484	0.963302606
0.926613578	0.929282568	0.963291555
0.92264919	0.929615652	0.963280102

0.922671655	0.929948736	0.963268661
0.922219775	0.93028182	0.963256839
0.922242521	0.930614904	0.963245027
0.920805641	0.930947988	0.96323318
0.920829289	0.931281072	0.963221344
0.921345558	0.931614156	0.963209374
0.91936173	0.93194724	0.963197415
0.921392159	0.932280324	0.963185514
0.921415437	0.932613408	0.963173426
0.9214387	0.932946492	0.963161545
0.924716512	0.933279576	0.963149674
0.929227326	0.93361266	0.963137813
0.929245218	0.933945744	0.963126283
0.932418773	0.934278828	0.963115207
0.933079609	0.934611912	0.963104142
0.933094749	0.934944996	0.963093398
0.935508478	0.93527808	0.963082729
0.933124997	0.935611164	0.96307207
0.932496683	0.935944248	0.96306166
0.935548972	0.936277332	0.963051025
0.93252777	0.936610416	0.963040341
0.929030323	0.9369435	0.963029969
0.924526931	0.937276584	0.963019309
0.924548139	0.937609668	0.963008317
0.924569332	0.937942752	0.962996891
0.924590511	0.938275836	0.962985478
0.924611675	0.93860892	0.962974078
0.923732479	0.938942004	0.96296269
0.917814397	0.939275088	0.962951316
0.917839836	0.939608172	0.962939865
0.92556871	0.939941256	0.962927842
0.9255892	0.94027434	0.962915833
0.92727758	0.940607424	0.962904595
0.918470173	0.940940508	0.962893369
0.918495234	0.941273592	0.962882319
0.927335403	0.941606676	0.962870411
0.92735465	0.94193976	0.962858516
0.938420197	0.942272844	0.9628475
0.946941013	0.942272844	0.962836495
0.942587545	0.942272844	0.962826589
0.946293402	0.942272844	0.962817531
0.947654207	0.942272844	0.962808052
0.951869869	0.942272844	0.962798945
0.951882715	0.942272844	0.96278998

0.947687006	0.942272844	0.962781437
0.947697947	0.942272844	0.962772905
0.94547778	0.942272844	0.962763969
0.945488181	0.942272844	0.962755044
0.94398166	0.942272844	0.962745908
0.945508987	0.942272844	0.962736783
0.94400225	0.942272844	0.962727518
0.943364002	0.942272844	0.962718412
0.943374321	0.942272844	0.962709167
0.942733341	0.942272844	0.962699869
0.942743725	0.942272844	0.96269058
0.942754107	0.942272844	0.962681237
0.94298181	0.942272844	0.962671903
0.939381983	0.942272844	0.96266258
0.943002515	0.942272844	0.962653288
0.939404718	0.942272844	0.962643651
0.943023216	0.942272844	0.96263438
0.943033565	0.942272844	0.962624765
0.940604292	0.942272844	0.962615514
0.942181036	0.942272844	0.962606274
0.946325445	0.942272844	0.962596802
0.942201981	0.942272844	0.962587494
0.94395088	0.942272844	0.962578602
0.947027183	0.942272844	0.962569313
0.942888685	0.942272844	0.962560204
0.947048658	0.942272844	0.962551406
0.943775936	0.942272844	0.962542208
0.943786234	0.942272844	0.962533428
0.947080893	0.942272844	0.962524332
0.943806831	0.942272844	0.962515246
0.940506191	0.942272844	0.962506492
0.943827427	0.942272844	0.962497425
0.941432588	0.942272844	0.962488042
0.940308945	0.942272844	0.962478995
0.941229913	0.942272844	0.962469721
0.941240612	0.942272844	0.962460345
0.940341927	0.942272844	0.962451068
0.940352913	0.942272844	0.962441801
0.940363895	0.942272844	0.962432455
0.93871443	0.942272844	0.96242312
0.938969626	0.942272844	0.962413795
0.936690382	0.942272844	0.962404317
0.936703158	0.942272844	0.962394875
0.937245238	0.942272844	0.962385217

0.936994802	0.942272844	0.96237557
0.937007399	0.942272844	0.962365988
0.937019989	0.942272844	0.96235639
0.937295008	0.942272844	0.962346805
0.937307433	0.942272844	0.962337231
0.937834642	0.942272844	0.962327694
0.940265028	0.942272844	0.96231817
0.940276042	0.942272844	0.962308709
0.937871004	0.942272844	0.962299499
0.940298058	0.942272844	0.962290302
0.94030906	0.942272844	0.962280879
0.937652074	0.942272844	0.962271706
0.940331051	0.942272844	0.962262544
0.940110805	0.942272844	0.962253133
0.937943553	0.942272844	0.962243997
0.937955622	0.942272844	0.962234851
0.937967684	0.942272844	0.962225504
0.93797974	0.942272844	0.962216169
0.938242946	0.942272844	0.962206848
0.938254859	0.942272844	0.962197539
0.94041886	0.942272844	0.962188269
0.940429818	0.942272844	0.96217901
0.938290559	0.942272844	0.962169977
0.940451723	0.942272844	0.962160956
0.942473695	0.942272844	0.962151736
0.940473613	0.942272844	0.962142742
0.940484552	0.942272844	0.962133959
0.942504957	0.942272844	0.962124989
0.942515374	0.942272844	0.962116032
0.940287453	0.942272844	0.962107285
0.940298459	0.942272844	0.962098551
0.943632306	0.942272844	0.962089608
0.94364261	0.942272844	0.962080679
0.943652913	0.942272844	0.96207209
0.946273296	0.942272844	0.962063515
0.946283842	0.942272844	0.962054954
0.94149691	0.942272844	0.962046665
0.937456116	0.942272844	0.962038391
0.93746845	0.942272844	0.962029658
0.942629858	0.942272844	0.962020543
0.938993274	0.942272844	0.962011443
0.93698246	0.942272844	0.962002866
0.939016359	0.942272844	0.961993945
0.939027893	0.942272844	0.961984839

0.939039422	0.942272844	0.961975949
0.939050946	0.942272844	0.961967075
0.939062465	0.942272844	0.961958195
0.939552662	0.942272844	0.961949307
0.942286557	0.942272844	0.961940411
0.939810879	0.942272844	0.961931553
0.938374081	0.942272844	0.961922955
0.941209118	0.942272844	0.961914102
0.941219823	0.942272844	0.961905098
0.938409601	0.942272844	0.961896362
0.938421428	0.942272844	0.961887617
0.94213983	0.942272844	0.961878583
0.942150312	0.942272844	0.961869537
0.942160791	0.942272844	0.961860845
0.942171269	0.942272844	0.961852141
0.945874762	0.942272844	0.961843425
0.945885228	0.942272844	0.961834699
0.945895696	0.942272844	0.961826327
0.94177209	0.942272844	0.961817943
0.944177704	0.942272844	0.96180955
0.944620504	0.942272844	0.96180074
0.94068121	0.942272844	0.961792156
0.942910114	0.942272844	0.961783605
0.944651444	0.942272844	0.961774655
0.942930835	0.942272844	0.961765911
0.940724661	0.942272844	0.961757325
0.940735515	0.942272844	0.961748557
0.940746366	0.942272844	0.96173956
0.942754918	0.942272844	0.961730551
0.940539987	0.942272844	0.961721532
0.940550903	0.942272844	0.961712697
0.940100364	0.942272844	0.961703633
0.940111439	0.942272844	0.961694557
0.938195919	0.942272844	0.961685424
0.939665236	0.942272844	0.961676281
0.941507758	0.942272844	0.961666936
0.94174078	0.942272844	0.961657725
0.94130547	0.942272844	0.961648682
0.941316148	0.942272844	0.961639651
0.941326823	0.942272844	0.961630562
0.940659576	0.942272844	0.961621462
0.941348166	0.942272844	0.961612349
0.941358833	0.942272844	0.961603158
0.941369496	0.942272844	0.961594023

0.946179088	0.942272844	0.961584878
0.941390816	0.942272844	0.961575721
0.940033936	0.942272844	0.961567026
0.944466363	0.942272844	0.961557847
0.942087882	0.942272844	0.961548524
0.942098374	0.942272844	0.961539628
0.948994997	0.942272844	0.961530486
0.945157451	0.942272844	0.961521335
0.941465343	0.942272844	0.961512852
0.945178171	0.942272844	0.961503979
0.945188533	0.942272844	0.961494733
0.945198896	0.942272844	0.961485842
0.94520926	0.942272844	0.961476943
0.945219625	0.942272844	0.961468034
0.942192709	0.942272844	0.961459118
0.942203181	0.942272844	0.961450194
0.942213651	0.942272844	0.961440964
0.944394349	0.942272844	0.961431726
0.944404651	0.942272844	0.961422482
0.939280856	0.942272844	0.961413444
0.938065081	0.942272844	0.961404399
0.938575204	0.942272844	0.961394842
0.942056776	0.942272844	0.961385157
0.939086145	0.942272844	0.961375514
0.938855096	0.942272844	0.961366204
0.941867539	0.942272844	0.961356591
0.941878077	0.942272844	0.961346946
0.939132122	0.942272844	0.961337587
0.940089515	0.942272844	0.961328218
0.942568411	0.942272844	0.961318569
0.944312322	0.942272844	0.961309003
0.944755105	0.942272844	0.961299671
0.942599629	0.942272844	0.961290499
0.942610032	0.942272844	0.961281359
0.942620433	0.942272844	0.961271997
0.942630832	0.942272844	0.961262627
0.939945494	0.942272844	0.961253247
0.942651627	0.942272844	0.961243857
0.94178353	0.942272844	0.961234192
0.939028974	0.942272844	0.961224783
0.938797388	0.942272844	0.961215277
0.938315567	0.942272844	0.961205488
0.938327442	0.942272844	0.961195665
0.938832283	0.942272844	0.961185786

0.938598811	0.942272844	0.961175898
0.938610544	0.942272844	0.961166049
0.942962502	0.942272844	0.961156167
0.938386729	0.942272844	0.961146275
0.939132242	0.942272844	0.961136801
0.943643415	0.942272844	0.961126866
0.943653718	0.942272844	0.961116995
0.943664021	0.942272844	0.961107559
0.943674324	0.942272844	0.961098114
0.946740259	0.942272844	0.96108866
0.946750917	0.942272844	0.961079196
0.939923326	0.942272844	0.961070023
0.93993447	0.942272844	0.96106084
0.944806828	0.942272844	0.961050973
0.944817154	0.942272844	0.961041098
0.940887721	0.942272844	0.961031691
0.945489969	0.942272844	0.961022275
0.949386782	0.942272844	0.961012463
0.945292704	0.942272844	0.961003094
0.940930908	0.942272844	0.960994097
0.940941696	0.942272844	0.960984688
0.942506422	0.942272844	0.960974838
0.942516839	0.942272844	0.960964979
0.942527254	0.942272844	0.960955263
0.942537668	0.942272844	0.960945538
0.944497869	0.942272844	0.960935805
0.944508175	0.942272844	0.960926063
0.944518482	0.942272844	0.960916503
0.944528789	0.942272844	0.960906936
0.944539097	0.942272844	0.960897359
0.946074662	0.942272844	0.960887775
0.946085166	0.942272844	0.960878184
0.944570023	0.942272844	0.960868738
0.944580333	0.942272844	0.960859285
0.948385525	0.942272844	0.960849675
0.947244237	0.942272844	0.96084006
0.94178375	0.942272844	0.960830811
0.939744933	0.942272844	0.960821442
0.939756149	0.942272844	0.960811528
0.941815415	0.942272844	0.960801404
0.941825965	0.942272844	0.960791272
0.93978977	0.942272844	0.960781332
0.939086804	0.942272844	0.960771383
0.938363588	0.942272844	0.960761223

0.938375439	0.942272844	0.960750984
0.939121294	0.942272844	0.960740662
0.93913278	0.942272844	0.960730331
0.939384056	0.942272844	0.960720064
0.943004402	0.942272844	0.960709787
0.938681374	0.942272844	0.960699524
0.938693068	0.942272844	0.960689608
0.939190133	0.942272844	0.960679257
0.943045799	0.942272844	0.960668899
0.944353843	0.942272844	0.960658581
0.940628061	0.942272844	0.960648631
0.940638947	0.942272844	0.960638801
0.941551346	0.942272844	0.960628594
0.94156196	0.942272844	0.960618378
0.941572571	0.942272844	0.960608242
0.943767514	0.942272844	0.960598097
0.943777813	0.942272844	0.960587943
0.941604388	0.942272844	0.960577995
0.941614988	0.942272844	0.960568039
0.94337631	0.942272844	0.960557859
0.943386628	0.942272844	0.96054767
0.94005729	0.942272844	0.960537645
0.938634666	0.942272844	0.96052761
0.942329396	0.942272844	0.960517237
0.942339843	0.942272844	0.960506713
0.938669797	0.942272844	0.960496544
0.938681496	0.942272844	0.960486366
0.944755803	0.942272844	0.960475819
0.940595495	0.942272844	0.960465263
0.939677773	0.942272844	0.960455295
0.941296012	0.942272844	0.960444907
0.941306693	0.942272844	0.96043442
0.941540841	0.942272844	0.960424082
0.940649943	0.942272844	0.960413736
0.939733946	0.942272844	0.960403404
0.940671697	0.942272844	0.960392977
0.94158329	0.942272844	0.960382449
0.940693436	0.942272844	0.960372004
0.939778801	0.942272844	0.96036164
0.939075536	0.942272844	0.960351179
0.939087043	0.942272844	0.96034062
0.940046309	0.942272844	0.960329983
0.939110042	0.942272844	0.960319339
0.943190666	0.942272844	0.96030878

0.944931384	0.942272844	0.96029812
0.941006972	0.942272844	0.960287853
0.941017737	0.942272844	0.960277748
0.944962396	0.942272844	0.960267248
0.944972735	0.942272844	0.96025674
0.947646183	0.942272844	0.960246613
0.945428289	0.942272844	0.960236478
0.943273571	0.942272844	0.960226599
0.945449078	0.942272844	0.960216493
0.943294222	0.942272844	0.960206167
0.944817853	0.942272844	0.960196049
0.947029643	0.942272844	0.960185711
0.944838507	0.942272844	0.960175518
0.944848835	0.942272844	0.960165536
0.946391248	0.942272844	0.960155333
0.944869494	0.942272844	0.960145125
0.945314159	0.942272844	0.960135061
0.946422973	0.942272844	0.96012484
0.945334917	0.942272844	0.960114655
0.946444135	0.942272844	0.960104572
0.945792899	0.942272844	0.960094373
0.945803351	0.942272844	0.960084275
0.945813804	0.942272844	0.960074106
0.948543256	0.942272844	0.96006393
0.945179736	0.942272844	0.960053747
0.945190098	0.942272844	0.960043825
0.942818858	0.942272844	0.960033564
0.94282923	0.942272844	0.960023298
0.943273679	0.942272844	0.96001279
0.940168251	0.942272844	0.960002276
0.94329433	0.942272844	0.959991797
0.940190347	0.942272844	0.959981003
0.940201389	0.942272844	0.959970507
0.940212426	0.942272844	0.959959696
0.939991284	0.942272844	0.959948877
0.940693946	0.942272844	0.95993805
0.94070481	0.942272844	0.959927192
0.94071567	0.942272844	0.959916393
0.945761937	0.942272844	0.959905586
0.945772383	0.942272844	0.95989477
0.939824399	0.942272844	0.959884441
0.946677588	0.942272844	0.959874103
0.94668823	0.942272844	0.95986317
0.941456423	0.942272844	0.959852902

0.947613651	0.942272844	0.959842626
0.940802426	0.942272844	0.959831825
0.947635487	0.942272844	0.959821622
0.94719113	0.942272844	0.959810739
0.940834902	0.942272844	0.959800521
0.947212701	0.942272844	0.959790248
0.947223491	0.942272844	0.95977934
0.941093224	0.942272844	0.959769051
0.942434379	0.942272844	0.959758753
0.94725588	0.942272844	0.959747843
0.942016184	0.942272844	0.959737055
0.945720009	0.942272844	0.959726732
0.942037195	0.942272844	0.959715883
0.940704923	0.942272844	0.959705389
0.942058197	0.942272844	0.959694522
0.94072664	0.942272844	0.959683514
0.942079191	0.942272844	0.959672629
0.942528176	0.942272844	0.959661605
0.942319928	0.942272844	0.959650705
0.942330376	0.942272844	0.95963984
0.943644971	0.942272844	0.959628946
0.942351267	0.942272844	0.959618045
0.943665577	0.942272844	0.959607264
0.94367588	0.942272844	0.959596346
0.943686182	0.942272844	0.959585548
0.943696484	0.942272844	0.959574742
0.945439549	0.942272844	0.959563927
0.94350105	0.942272844	0.959553103
0.942860826	0.942272844	0.959542442
0.94352167	0.942272844	0.95953158
0.940889353	0.942272844	0.959520645
0.947040994	0.942272844	0.959509766
0.945501943	0.942272844	0.959498617
0.950393787	0.942272844	0.959488065
0.945741269	0.942272844	0.959477353
0.950417953	0.942272844	0.959467114
0.945762155	0.942522657	0.959456407
0.950442143	0.94277247	0.95944615
0.950454247	0.943022283	0.959435426
0.948275521	0.943272096	0.959425152
0.948286671	0.94352191	0.959414871
0.948297826	0.943771723	0.959404364
0.948308985	0.944021536	0.959393849
0.951822824	0.944271349	0.959383326

0.957919032	0.944521162	0.959372794
0.953521537	0.944770975	0.959362599
0.957951759	0.945020788	0.959352995
0.953549093	0.945270601	0.959342947
0.953562883	0.945520414	0.959333326
0.953576681	0.945770227	0.959323262
0.953590486	0.946020041	0.959313189
0.946139308	0.946269854	0.959303108
0.946149825	0.946519667	0.959293018
0.945502051	0.94676948	0.959282182
0.945512455	0.947019293	0.959271337
0.945522861	0.947269106	0.959260417
0.94205869	0.947518919	0.959249488
0.945543678	0.947768732	0.95923855
0.946880758	0.948018545	0.959227262
0.949703804	0.948268358	0.959216305
0.950721742	0.948518172	0.95920547
0.952327587	0.948767985	0.959194903
0.951797337	0.949017798	0.959184423
0.955588353	0.949267611	0.959174089
0.953207196	0.949517424	0.959163691
0.955618333	0.949767237	0.959153653
0.961582872	0.95001705	0.959143369
0.955648348	0.950266863	0.959133311
0.960478983	0.950516676	0.959123826
0.964452486	0.950766489	0.959113746
0.958001092	0.951016303	0.959104128
0.957675129	0.951266116	0.959094889
0.957691345	0.951515929	0.959085003
0.957707571	0.951765742	0.959075072
0.956394198	0.952015555	0.959065132
0.950648855	0.952265368	0.959055183
0.950661062	0.952515181	0.959045093
0.952262683	0.952764994	0.959034425
0.950685494	0.953014807	0.959023747
0.950697719	0.95326462	0.959013214
0.949458809	0.953514434	0.959002512
0.95098049	0.953764247	0.958991798
0.949482094	0.95401406	0.958980948
0.946255231	0.954263873	0.958970235
0.947614674	0.954513686	0.958959362
0.95236738	0.954763499	0.95894816
0.949774515	0.955013312	0.958937081
0.946519108	0.955263125	0.958926459

0.949798089	0.955512938	0.958915572
0.946540316	0.955762751	0.958904353
0.952158584	0.956012565	0.958893448
0.951631735	0.956262378	0.958882212
0.951644453	0.956512191	0.958871519
0.955723698	0.956762004	0.958860764
0.954807648	0.957011817	0.958849998
0.952777378	0.95726163	0.958839624
0.954836705	0.957511443	0.958829148
0.954851246	0.957761256	0.958818461
0.952817448	0.958011069	0.958807967
0.95283082	0.958260882	0.958797464
0.9528442	0.958510696	0.95878675
0.95045494	0.958760509	0.958776026
0.95046705	0.959010322	0.958765292
0.950479166	0.959260135	0.958754313
0.950491289	0.959509948	0.958743325
0.954064815	0.959759761	0.95873233
0.954078904	0.960009574	0.958721326
0.953798823	0.960259387	0.958710665
0.953812757	0.9605092	0.958699995
0.970171355	0.960759013	0.958689286
0.95384065	0.961008827	0.958678569
0.970215933	0.96125864	0.958669452
0.977595502	0.961508453	0.958658716
0.965336372	0.961758266	0.958649582
0.965356673	0.962008079	0.958641164
0.977668315	0.962257892	0.958631527
0.977692595	0.962507705	0.958621881
0.984456136	0.962757518	0.958613435
0.987100571	0.963007331	0.95860498
0.987125528	0.963257144	0.958597177
0.987150484	0.963506958	0.958589623
0.999886816	0.963756771	0.958582058
0.999909881	0.964006584	0.958574483
0.99993294	0.964256397	0.958568149
0.97839828	0.96450621	0.958561805
0.968677006	0.964756023	0.958555449
0.968698735	0.965005836	0.958546959
0.968720472	0.965255649	0.958537497
0.968742218	0.965505462	0.958528024
0.968763972	0.965755275	0.958518538
0.966549686	0.966005089	0.95850904
0.966570534	0.966254902	0.958499532

0.979621839	0.966504715	0.958489794
0.979646422	0.966754528	0.958480045
0.991146897	0.967004341	0.958471569
0.991171572	0.967254154	0.958463082
0.993255689	0.967503967	0.958455714
0.97467444	0.96775378	0.958448352
0.971294284	0.968003593	0.9584412
0.977232532	0.968253406	0.958432222
0.977256733	0.96850322	0.958422915
0.977280938	0.968753033	0.958414196
0.977305147	0.969002846	0.958405483
0.977329361	0.969252659	0.958396776
0.974840062	0.969502472	0.958388075
0.974863746	0.969752285	0.95837938
0.971476433	0.970002098	0.958370446
0.971499161	0.970251911	0.958361517
0.963531574	0.970501724	0.958352259
0.963551004	0.970751537	0.958343007
0.96566307	0.971001351	0.958332974
0.957953464	0.971251164	0.958322948
0.956957371	0.971500977	0.958313136
0.956973166	0.97175079	0.958302569
0.95698897	0.972000603	0.95829191
0.966197629	0.972250416	0.958281258
0.977136659	0.972500229	0.958270612
0.977160841	0.972750042	0.958260878
0.977185027	0.972999855	0.958252226
0.977209219	0.973249668	0.958243581
0.974227457	0.973499482	0.958234944
0.9777666	0.973749295	0.958226311
0.977790897	0.973999108	0.958217388
0.990826401	0.974248921	0.958208816
0.99085111	0.974498734	0.958200248
1.010309238	0.974748547	0.958192968
0.989341082	0.97499836	0.958185692
0.989365923	0.975248173	0.958180334
0.989390761	0.975497986	0.958172914
0.98470631	0.975747799	0.958165499
0.993572915	0.975997613	0.95815809
0.983186136	0.976247426	0.958150223
0.983211049	0.976497239	0.958143233
0.979083269	0.976747052	0.958135223
0.972115567	0.976996865	0.95812722
0.972138497	0.977246678	0.958118814

0.970261821	0.977496491	0.958109727
0.97713691	0.977746304	0.958100646
0.977161093	0.977996117	0.958091385
0.967137095	0.97824593	0.958082805
0.965419329	0.978495744	0.958074231
0.968982432	0.978745557	0.958064674
0.969004279	0.97899537	0.958054953
0.969026134	0.979245183	0.958045587
0.979353051	0.979494996	0.958036228
0.96906987	0.979744809	0.958026875
0.96773109	0.979994622	0.958018542
0.966425977	0.980244435	0.9580092
0.967773799	0.980494248	0.95799973
0.967795166	0.980744061	0.957990135
0.976438808	0.980993875	0.957980675
0.976462851	0.981243688	0.957971219
0.9764869	0.981493501	0.957962617
0.976510954	0.981743314	0.957954019
0.967902136	0.981993127	0.957945424
0.967923556	0.98224294	0.957936834
0.976079628	0.982492753	0.957927398
0.976607218	0.982742566	0.957917969
0.968895356	0.982992379	0.957909346
0.977161974	0.983242192	0.95790078
0.968938991	0.983492006	0.957891457
0.968960821	0.983741819	0.957882952
0.981349768	0.983991632	0.957873641
0.970867117	0.984241445	0.957864336
0.970889643	0.984491258	0.957856254
0.974299168	0.984741071	0.957847142
0.972852794	0.984990884	0.957838036
0.974842036	0.985240697	0.957829269
0.97486572	0.98549051	0.957820361
0.973410026	0.985740323	0.957811651
0.974913105	0.985990137	0.957802947
0.973456653	0.98623995	0.957794102
0.972991804	0.986489763	0.957785407
0.973503306	0.986739576	0.957776572
0.975007945	0.986989389	0.957767696
0.980633422	0.987239202	0.957758873
0.988497833	0.987489015	0.957750202
0.988522729	0.987738828	0.957742089
0.988547623	0.987988641	0.957734753
0.988572516	0.988238454	0.957727421

0.988597408	0.988488268	0.957720095
0.988622298	0.988738081	0.957712774
0.995340938	0.988987894	0.957705459
0.988672073	0.989237707	0.95769815
0.994883233	0.98948752	0.957691506
0.989764269	0.989737333	0.957684208
0.990308629	0.989987146	0.957677525
0.977840264	0.990236959	0.957670341
0.989317664	0.990486772	0.957663215
0.998985719	0.990736585	0.957654864
0.987800731	0.990986399	0.957647646
0.999032286	0.991236212	0.957641382
0.999055562	0.991486025	0.957634019
1.000049946	0.991735838	0.957627766
0.998612623	0.991985651	0.957621517
1.000095988	0.992235464	0.957615371
0.998659367	0.992485277	0.957609087
1.000142005	0.99273509	0.957602953
0.99870609	0.992984903	0.95759668
0.989069404	0.993234716	0.957590556
1.002118913	0.99348453	0.957584294
1.00400383	0.993734343	0.957577087
1.004025751	0.993984156	0.957571168
0.993307035	0.994233969	0.957565439
1.005884455	0.994483782	0.957559715
0.988697218	0.994733595	0.957552939
0.995413354	0.994983408	0.957547404
0.9954374	0.995233221	0.957540179
0.995461442	0.995483034	0.957533618
0.995485479	0.995732847	0.957527062
0.995509512	0.995982661	0.95752051
0.995533541	0.996232474	0.957513962
0.979993484	0.996482287	0.957507417
0.995581584	0.9967321	0.957500875
0.985781414	0.996981913	0.957492803
0.989988362	0.997231726	0.957486266
1.002477661	0.997481539	0.957478764
1.002500029	0.997731352	0.957471677
1.009651992	0.997981165	0.957465819
1.010923239	0.998230978	0.957459964
1.009692174	0.998480792	0.957454812
1.009712254	0.998730605	0.957449786
1.009732328	0.998980418	0.95744464
1.00402655	0.999230231	0.957439496

1.013440703	0.999480044	0.957434355
1.01345951	0.999729857	0.957428653
1.004092269	0.99997967	0.957423878
1.004114163	1.000229483	0.957419105
1.006837661	1.000479296	0.957413411
1.006858691	1.000729109	0.957407721
1.006879715	1.000978923	0.957402302
0.992966252	1.001228736	0.957396886
0.992990699	1.001478549	0.957391475
0.991470279	1.001728362	0.957384695
0.987328274	1.001978175	0.957377919
0.9925504	1.002227988	0.957370994
1.005671716	1.002477801	0.957363663
0.992599404	1.002727614	0.957356849
0.992623901	1.002977427	0.95735133
0.992648395	1.00322724	0.957344524
0.994715509	1.003477054	0.957337724
0.997746169	1.003726867	0.957330928
0.998753885	1.00397668	0.957324339
1.010982922	1.004226493	0.957318051
1.011002564	1.004476306	0.957311864
1.010192669	1.004726119	0.957306884
0.998847219	1.004975932	0.957301906
0.999358717	1.005225745	0.95729685
0.998893852	1.005475558	0.957290677
0.999405104	1.005725371	0.957284556
1.006859458	1.005975185	0.95727839
1.016619361	1.006224998	0.957272275
1.011975776	1.006474811	0.957266894
1.011995079	1.006724624	0.957262474
1.016672639	1.006974437	0.957257597
1.01243656	1.00722425	0.957252722
1.012455707	1.007474063	0.957248308
1.01891572	1.007723876	0.957243478
1.018932788	1.007973689	0.957238651
1.01251311	1.008223502	0.95723446
1.01824651	1.008473316	0.957230273
1.011335455	1.008723129	0.957225455
1.010529718	1.008972942	0.957221204
1.010549515	1.009222755	0.957216274
1.010569304	1.009472568	0.957211268
1.012627739	1.009722381	0.957206266
1.017254755	1.009972194	0.957201269
1.011860016	1.010222007	0.957196478

1.01187936	1.01047182	0.957192144
1.011898696	1.010721633	0.957187281
1.004137075	1.010971447	0.957182422
1.01193735	1.01122126	0.957177567
1.016619546	1.011471073	0.95717195
1.008641254	1.011720886	0.957167102
1.001421622	1.011970699	0.957162717
1.000488295	1.012220512	0.957157548
1.000511206	1.012470325	0.95715167
1.001489589	1.012720138	0.957145704
1.001512233	1.012969951	0.957139742
1.005694123	1.013219764	0.95713388
1.010471012	1.013469578	0.957128021
1.010490828	1.013719391	0.957122574
1.010510638	1.013969204	0.957117599
1.017917857	1.014219017	0.957112625
1.017935221	1.01446883	0.957107653
1.023183605	1.014718643	0.957103411
1.023199584	1.014968456	0.957099171
1.017987279	1.015218269	0.957095448
1.020497921	1.015468082	0.957091727
1.02051455	1.015717895	0.957087494
1.020531175	1.015967709	0.957083509
1.016209769	1.016217522	0.957079526
1.016227664	1.016467335	0.957075547
1.011550033	1.016717148	0.957071143
1.016263437	1.016966961	0.957066743
1.01035218	1.017216774	0.957061884
1.010372037	1.017466587	0.957057491
1.016317054	1.0177164	0.957052517
1.016334914	1.017966213	0.957047548
1.021027714	1.018216026	0.957043166
1.016370619	1.01846584	0.95703879
1.012111166	1.018715653	0.95703488
1.009230551	1.018965466	0.957030514
1.0088246	1.019215279	0.957025732
1.008844973	1.019465092	0.957020671
1.023502529	1.019714905	0.957015573
1.032470069	1.019964718	0.957010479
1.032484923	1.020214531	0.957006832
1.022880094	1.020464344	0.957004071
1.02289614	1.020714157	0.957001313
1.022912182	1.020963971	0.95699761
1.02292822	1.021213784	0.956993911

1.022268414	1.021463597	0.956990215
1.032574038	1.02171341	0.956986523
1.022300781	1.021963223	0.956982767
1.022316959	1.022213036	0.956980028
1.010352904	1.022462849	0.956976282
1.01037276	1.022712662	0.956972539
1.016317705	1.022962475	0.956967622
1.010412453	1.023212288	0.95696271
1.01635342	1.023462102	0.956958387
1.023423506	1.023711915	0.956953486
1.023439433	1.023961728	0.956949175
1.023455357	1.024211541	0.956945564
1.023471278	1.024211541	0.95694196
1.017553463	1.024211541	0.956938361
1.023503109	1.024211541	0.956934768
1.026146394	1.024211541	0.956930598
1.020115184	1.024211541	0.956927019
1.025201611	1.024211541	0.956923707
1.025543621	1.024211541	0.956919808
1.019459837	1.024211541	0.956916416
1.020531954	1.024211541	0.956913063
1.020548574	1.024211541	0.956909115
1.021944659	1.024211541	0.956905278
1.021960922	1.024211541	0.956901445
1.02197718	1.024211541	0.956897753
1.017762801	1.024211541	0.956894063
1.019931445	1.024211541	0.956890377
1.017797617	1.024211541	0.956886277
1.014816424	1.024211541	0.956882392
1.017832411	1.024211541	0.956878299
1.016371455	1.024211541	0.956873915
1.016389299	1.024211541	0.956869831
1.017150436	1.024211541	0.956865606
1.016797957	1.024211541	0.956861387
1.016815665	1.024211541	0.956857248
1.017203215	1.024211541	0.956853078
1.019392314	1.024211541	0.956848913
1.024199497	1.024211541	0.956844791
1.025853584	1.024211541	0.956840889
1.024231031	1.024211541	0.956837463
1.024246793	1.024211541	0.956834203
1.024262553	1.024211541	0.956830789
1.021928139	1.024211541	0.95682738
1.024294062	1.024211541	0.956823975

1.021960668	1.024211541	0.956820342
1.024325559	1.024211541	0.956816945
1.021993181	1.024211541	0.956813321
1.023360095	1.024211541	0.956809932
1.023376036	1.024211541	0.956806316
1.024058016	1.024211541	0.956802838
1.024073813	1.024211541	0.956799365
1.02104515	1.024211541	0.956795962
1.022430857	1.024211541	0.956792563
1.019679951	1.024211541	0.956788867
1.022463148	1.024211541	0.95678531
1.019713649	1.024211541	0.956781484
1.022495425	1.024211541	0.956777935
1.019747328	1.024211541	0.956774117
1.019764161	1.024211541	0.956770576
1.015382891	1.024211541	0.956766765
1.016532491	1.024211541	0.956762959
1.018746726	1.024211541	0.956758724
1.0158165	1.024211541	0.956754604
1.019138553	1.024211541	0.956750706
1.015852537	1.024211541	0.956746521
1.01699263	1.024211541	0.956742667
1.017010278	1.024211541	0.956738492
1.01920654	1.024211541	0.956734432
1.019223525	1.024211541	0.956730379
1.019240505	1.024211541	0.956726545
1.022042514	1.024211541	0.956722718
1.019629105	1.024211541	0.956718896
1.022074988	1.024211541	0.956715354
1.019662831	1.024211541	0.956711578
1.022107447	1.024211541	0.956708045
1.022123671	1.024211541	0.956704278
1.023488107	1.024211541	0.956700755
1.021127709	1.024211541	0.956697238
1.016478802	1.024211541	0.95669386
1.021160635	1.024211541	0.956690254
1.016514415	1.024211541	0.956686194
1.022220931	1.024211541	0.956682598
1.022237128	1.024211541	0.956678548
1.022253321	1.024211541	0.956675064
1.014688872	1.024211541	0.956671585
1.014320458	1.024211541	0.956668113
1.01433897	1.024211541	0.9566639
1.014357477	1.024211541	0.956659659

1.014762396	1.024211541	0.956655424
1.014004992	1.024211541	0.956651199
1.014799122	1.024211541	0.956647019
1.01443096	1.024211541	0.956642772
1.014449435	1.024211541	0.956638608
1.014854165	1.024211541	0.956634413
1.015255969	1.024211541	0.956630225
1.015274173	1.024211541	0.956626083
1.014909154	1.024211541	0.956621987
1.015691034	1.024211541	0.956617897
1.016461637	1.024211541	0.956613778
1.021144346	1.024211541	0.956609742
1.016497261	1.024211541	0.956605786
1.023217389	1.024211541	0.956602298
1.0222211	1.024211541	0.956598358
1.022237297	1.024211541	0.956595087
1.02225349	1.024211541	0.956591725
1.022269679	1.024211541	0.95658837
1.028178142	1.024211541	0.956585023
1.024310714	1.024211541	0.956581684
1.031357076	1.024211541	0.956578933
1.024342204	1.024211541	0.956575807
1.03138687	1.024211541	0.956573381
1.024373682	1.024211541	0.956570269
1.028586504	1.024211541	0.956567855
1.028601612	1.024211541	0.956564756
1.019291769	1.024211541	0.956562077
1.024765984	1.024211541	0.956559404
1.024781644	1.024211541	0.956555819
1.031178408	1.024211541	0.956552777
1.026440102	1.024211541	0.956549741
1.026455482	1.024211541	0.95654734
1.026470859	1.024211541	0.956544476
1.026486235	1.024211541	0.956541603
1.026501608	1.024211541	0.95653872
1.026516978	1.024211541	0.956535829
1.026532347	1.024211541	0.956532927
1.027190397	1.024211541	0.956530016
1.023948423	1.024211541	0.956527097
1.023964242	1.024211541	0.956524233
1.01917353	1.024211541	0.95652104
1.019190524	1.024211541	0.956517839
1.019207514	1.024211541	0.956514155
1.019224498	1.024211541	0.956510463

1.019241478	1.024211541	0.956506762
1.014818336	1.024211541	0.956503051
1.021718016	1.024211541	0.95649933
1.01637285	1.024211541	0.956495161
1.022769683	1.024211541	0.956491658
1.016781627	1.024211541	0.956487615
1.028344879	1.024211541	0.956484188
1.037113598	1.024211541	0.956480159
1.027099269	1.024211541	0.956477255
1.037143364	1.024211541	0.9564752
1.027129839	1.024211541	0.956472145
1.034058451	1.024211541	0.956470065
1.025218977	1.024211541	0.956466987
1.034088109	1.024211541	0.956464579
1.031610775	1.024211541	0.956461288
1.03162566	1.024211541	0.956458858
1.031640545	1.024211541	0.956456173
1.023648002	1.024211541	0.956453477
1.031670312	1.024211541	0.95645077
1.023679759	1.024211541	0.956447263
1.031700077	1.024211541	0.956444536
1.02437483	1.024211541	0.95644101
1.037649579	1.024211541	0.956438264
1.024735389	1.024211541	0.956434786
1.024751055	1.024211541	0.956432605
1.027678564	1.024211541	0.956429141
1.021079405	1.024211541	0.956425667
1.027708984	1.024211541	0.95642247
1.021112355	1.024211541	0.956418611
1.021128824	1.024211541	0.956415393
1.021145289	1.024211541	0.956411515
1.02320233	1.024211541	0.956407626
1.020134007	1.024211541	0.956403728
1.023234276	1.024211541	0.956400019
1.019461951	1.024211541	0.956395998
1.019478864	1.024211541	0.956392272
1.023616397	1.024211541	0.956388165
1.020567266	1.024211541	0.956384049
1.022302972	1.024211541	0.95638033
1.02231915	1.024211541	0.956376301
1.022335323	1.024211541	0.956372433
1.023695798	1.024211541	0.956368556
1.023711668	1.024211541	0.956364671
1.023727535	1.024211541	0.956360912

1.023743398	1.024211541	0.956357143
1.020700028	1.024211541	0.956353365
1.020716603	1.024211541	0.956349578
1.020034162	1.024211541	0.95634548
1.022464998	1.024211541	0.956341373
1.022481138	1.024211541	0.956337189
1.022497274	1.024211541	0.956333234
1.016853278	1.024211541	0.956329272
1.022529535	1.024211541	0.956325301
1.016888654	1.024211541	0.956320765
1.016159315	1.024211541	0.956316778
1.021898052	1.024211541	0.956312226
1.022255007	1.024211541	0.956307593
1.022610122	1.024211541	0.956303515
1.022626229	1.024211541	0.956299463
1.02594871	1.024211541	0.956295436
1.02465775	1.024211541	0.956291399
1.02662579	1.024211541	0.95628768
1.024689108	1.024211541	0.956283822
1.024704783	1.024211541	0.956280149
1.024720455	1.024211541	0.956276274
1.02240057	1.024211541	0.95627239
1.022416724	1.024211541	0.956268498
1.024107781	1.024211541	0.956264368
1.02312357	1.024211541	0.95626023
1.021441539	1.024211541	0.956256248
1.021457925	1.024211541	0.95625216
1.019378112	1.024211541	0.956247898
1.021490684	1.024211541	0.956243628
1.021507058	1.024211541	0.956239146
1.023219469	1.024211541	0.956234863
1.027800911	1.024211541	0.956230575
1.023251408	1.024211541	0.956226446
1.021228748	1.024211541	0.95622276
1.029115234	1.024211541	0.956218619
1.02913029	1.024211541	0.95621427
1.018817303	1.024211541	0.956210691
1.02628818	1.024211541	0.956207105
1.02026891	1.024211541	0.956202495
1.026318984	1.024211541	0.956198614
1.020302286	1.024211541	0.956194132
1.020318967	1.024211541	0.956190237
1.026365172	1.024211541	0.95618574
1.026380563	1.024211541	0.956181236

1.026395952	1.024211541	0.95617732
1.026411338	1.024211541	0.956173395
1.032428754	1.024211541	0.956169463
1.033688861	1.024211541	0.956165524
1.033703692	1.024211541	0.95616217
1.035897197	1.024211541	0.95615893
1.035912044	1.024211541	0.956155682
1.035926891	1.024211541	0.956152642
1.026840683	1.024211541	0.956149594
1.035956588	1.024211541	0.95614654
1.026871324	1.024211541	0.956142583
1.026564677	1.024211541	0.956139516
1.033511416	1.024211541	0.956135547
1.016249652	1.024211541	0.956131539
1.016267535	1.024211541	0.956128208
1.016285411	1.024211541	0.956123169
1.018509063	1.024211541	0.956118123
1.01852625	1.024211541	0.956113072
1.017818188	1.024211541	0.956108232
1.017835581	1.024211541	0.956103385
1.017852969	1.024211541	0.956098459
1.029265884	1.024378083	0.956093526
1.029280926	1.024544625	0.956088588
1.029295967	1.024711167	0.956084765
1.039937292	1.024877709	0.956080934
1.030583886	1.025044251	0.956077097
1.042183913	1.025210793	0.956074299
1.030613766	1.025377335	0.956070571
1.042214247	1.025543877	0.956067977
1.030956972	1.025710419	0.956064237
1.042244585	1.025876961	0.956061633
1.030986808	1.026043503	0.956057912
1.025267327	1.026210045	0.956055296
1.028180431	1.026376587	0.956051562
1.028195583	1.02654313	0.956047257
1.028210733	1.026709672	0.956043231
1.028225881	1.026876214	0.956039198
1.028241028	1.027042756	0.956035158
1.023379357	1.027209298	0.956031108
1.028271316	1.02737584	0.956027049
1.024737021	1.027542382	0.956022502
1.028301597	1.027708924	0.956018425
1.028633711	1.027875466	0.95601399
1.033348549	1.028042008	0.956009894

1.027390544	1.02820855	0.95600582
1.028679015	1.028375092	0.956002197
1.028694113	1.028541634	0.955997977
1.028709209	1.028708176	0.955993873
1.038097973	1.028874718	0.95598976
1.035927587	1.02904126	0.955985638
1.047710469	1.029207802	0.95598243
1.035957284	1.029374344	0.955978998
1.035972132	1.029540886	0.955976716
1.028165436	1.029707428	0.955973266
1.038500983	1.029873971	0.955969808
1.05307407	1.030040513	0.955965573
1.038530868	1.030207055	0.955962347
1.042973422	1.030373597	0.955960546
1.034193224	1.030540139	0.955957303
1.034208053	1.030706681	0.955954486
1.034222882	1.030873223	0.955950795
1.043034317	1.031039765	0.955947094
1.034563791	1.031206307	0.955943384
1.035512279	1.031372849	0.955940532
1.035527119	1.031539391	0.955936835
1.031493529	1.031705933	0.955933223
1.035556799	1.031872475	0.955929601
1.03152331	1.032039017	0.955925573
1.041866532	1.032205559	0.955921936
1.041881676	1.032372101	0.955917895
1.041896822	1.032538643	0.955914865
1.041911968	1.032705185	0.95591183
1.041927116	1.032871727	0.95590879
1.041624521	1.033038269	0.955905744
1.042912703	1.033204812	0.955902692
1.051726725	1.033371354	0.955899601
1.040403494	1.033537896	0.955896629
1.051758383	1.033704438	0.955894517
1.040433591	1.03387098	0.955891282
1.051790044	1.034037522	0.955889155
1.040463693	1.034204064	0.955885905
1.040478745	1.034370606	0.955883763
1.043034476	1.034537148	0.955880496
1.048880366	1.03470369	0.955877222
1.048896015	1.034870232	0.955874191
1.042760743	1.035036774	0.955871726
1.049584888	1.035203316	0.955869253
1.042791158	1.035369858	0.955866167

1.048958623	1.0355364	0.955863745
1.042821578	1.035702942	0.955860645
1.048989933	1.035869484	0.955858145
1.042852001	1.036036026	0.955855031
1.042867215	1.036202568	0.955852517
1.043202028	1.03636911	0.955849389
1.040358511	1.036535653	0.955846255
1.040373556	1.036702195	0.955843146
1.03600214	1.036868737	0.955839749
1.04040365	1.037035279	0.955836345
1.03603184	1.037201821	0.955832502
1.040433748	1.037368363	0.955829086
1.040448798	1.037534905	0.955825231
1.04046385	1.037701447	0.955821802
1.046238519	1.037867989	0.955818366
1.039234039	1.038034531	0.955814924
1.041776003	1.038201073	0.955812041
1.041791141	1.038367615	0.955808462
1.053217554	1.038534157	0.955805125
1.045022064	1.038700699	0.955801781
1.053583519	1.038867241	0.955799555
1.041217318	1.039033783	0.955796513
1.041232419	1.039200325	0.955794306
1.041247521	1.039366867	0.955790871
1.03374973	1.039533409	0.955787429
1.033141838	1.039699951	0.955783978
1.029086845	1.039866494	0.955779779
1.033171515	1.040033036	0.955775511
1.030689462	1.040199578	0.955770833
1.03320119	1.04036612	0.955766549
1.032904927	1.040532662	0.95576201
1.034164802	1.040699204	0.955757709
1.034179631	1.040865746	0.955753371
1.035750442	1.041032288	0.955749148
1.035454159	1.04119883	0.955744917
1.03827795	1.041365372	0.955740831
1.038292881	1.041531914	0.955736707
1.038307813	1.041698456	0.955732853
1.040840439	1.041864998	0.955728992
1.03833768	1.04203154	0.955725121
1.043096103	1.042198082	0.95572149
1.038367549	1.042364624	0.955717603
1.037443708	1.042531166	0.955714174
1.040916221	1.042697708	0.955710269

1.040931302	1.04286425	0.955706264
1.040946384	1.043030792	0.955702593
1.049680226	1.043197335	0.955698913
1.040976551	1.043363877	0.955695227
1.040991636	1.043530419	0.955692391
1.041006722	1.043696961	0.955688691
1.043889261	1.043863503	0.955684984
1.042306459	1.044030045	0.955681272
1.045529946	1.044196587	0.955677835
1.040434454	1.044363129	0.955674233
1.042670791	1.044529671	0.955670952
1.040464555	1.044696213	0.955667173
1.040479607	1.044862755	0.955663618
1.042716396	1.045029297	0.955659849
1.0427316	1.045195839	0.955656085
1.042746805	1.045362381	0.955652544
1.042762011	1.045528923	0.955649008
1.046641459	1.045695465	0.955645475
1.051917635	1.045862007	0.955641945
1.051933475	1.046028549	0.955638799
1.055291198	1.046195091	0.955636173
1.055307204	1.046361633	0.955633548
1.05801889	1.046528176	0.955631256
1.055339219	1.046694718	0.955628966
1.05805068	1.04686126	0.955626944
1.066901682	1.047027802	0.955624658
1.066917937	1.047194344	0.95562264
1.056749325	1.047360886	0.955621494
1.056765388	1.047527428	0.955620349
1.057793642	1.04769397	0.955618206
1.052108182	1.047860512	0.955616066
1.052124032	1.048027054	0.955614029
1.052139883	1.048193596	0.955611436
1.052155735	1.048360138	0.955608846
1.052171588	1.04852668	0.955606262
1.045622582	1.048693222	0.955603682
1.052203297	1.048859764	0.955601106
1.04662613	1.049026306	0.955597888
1.04664162	1.049192848	0.95559532
1.040254235	1.04935939	0.955592205
1.046997638	1.049525932	0.955589092
1.053950401	1.049692474	0.955585352
1.047028673	1.049859017	0.955582277
1.052313891	1.050025559	0.955579887

1.052329753	1.050192101	0.955576817
1.052345616	1.050358643	0.955574268
1.052361479	1.050525185	0.955571721
1.05404609	1.050691727	0.955569178
1.052393209	1.050858269	0.955566637
1.059130025	1.051024811	0.955564265
1.069014444	1.051191353	0.955561733
1.062555741	1.051357895	0.955559867
1.062571946	1.051524437	0.955558977
1.06258815	1.051690979	0.955557453
1.055835792	1.051857521	0.955555934
1.062280896	1.052024063	0.955554418
1.056540892	1.052190605	0.955552241
1.051538665	1.052357147	0.955550701
1.051886701	1.052523689	0.955548598
1.051902539	1.052690231	0.955546006
1.049602628	1.052856773	0.955543451
1.049618325	1.053023315	0.955540901
1.049634024	1.053189858	0.955538128
1.048335674	1.0533564	0.955535359
1.051981741	1.053522942	0.955532594
1.048366898	1.053689484	0.955529704
1.048382512	1.053856026	0.955527174
1.053696527	1.054022568	0.95552429
1.050388007	1.05418911	0.955521409
1.044854245	1.054355652	0.955519052
1.050419504	1.054522194	0.95551637
1.050435254	1.054688736	0.955513144
1.053107864	1.054855278	0.955510466
1.055804321	1.05502182	0.955507791
1.055820349	1.055188362	0.955505381
1.056172529	1.055354904	0.955503238
1.056862114	1.055521446	0.955501098
1.056878181	1.055687988	0.955498994
1.057569247	1.05585453	0.955496961
1.064711586	1.056021072	0.955494931
1.073609072	1.056187614	0.955492971
1.073625416	1.056354156	0.955491716
1.07364176	1.056520699	0.955491339
1.072972673	1.056687241	0.955490967
1.070935219	1.056853783	0.955490599
1.070951518	1.057020325	0.955490166
1.070967817	1.057186867	0.955489535
1.073723486	1.057353409	0.955488907

1.075113065	1.057519951	0.955488282
1.071016717	1.057686493	0.955487929
1.060470284	1.057853035	0.955487715
1.066951378	1.058019577	0.955487099
1.066967633	1.058186119	0.955485444
1.065279228	1.058352661	0.955484429
1.060534955	1.058519203	0.955483416
1.060551123	1.058685745	0.955482236
1.060567292	1.058852287	0.95548059
1.060583461	1.059018829	0.955478945
1.054860943	1.059185371	0.955477303
1.0606158	1.059351913	0.955475664
1.064030931	1.059518455	0.955473462
1.060987626	1.059684997	0.955471827
1.06474422	1.05985154	0.955470527
1.058309772	1.060018082	0.955468928
1.058325885	1.060184624	0.955467698
1.053299349	1.060351166	0.955465833
1.053315263	1.060517708	0.95546397
1.053331178	1.06068425	0.955461612
1.059405566	1.060850792	0.955459256
1.053696859	1.061017334	0.955456902
1.053712793	1.061183876	0.955455147
1.053728728	1.061350418	0.955452831
1.053076389	1.06151696	0.955450517
1.0537606	1.061683502	0.955448204
1.056125159	1.061850044	0.955445829
1.055468391	1.062016586	0.955443522
1.055484405	1.062183128	0.95544145
1.053155911	1.06234967	0.955439314
1.053171817	1.062516212	0.955437184
1.05285442	1.062682754	0.955434827
1.052870311	1.062849296	0.955432473
1.055228845	1.063015838	0.95543009
1.058615584	1.063182381	0.955427712
1.058970202	1.063348923	0.955425569
1.055612958	1.063515465	0.955423763
1.056301633	1.063682007	0.955421994
1.05631768	1.063848549	0.955419897
1.059034731	1.064015091	0.95541787
1.056349775	1.064181633	0.955415846
1.059066998	1.064348175	0.955414092
1.056381873	1.064514717	0.955412078
1.056397923	1.064681259	0.955410333

1.05139783	1.064847801	0.955408327
1.051413639	1.065014343	0.955406325
1.061181945	1.065180885	0.955403832
1.073479269	1.065347427	0.955401344
1.07349561	1.065513969	0.955399821
1.065653346	1.065680511	0.955399511
1.065669588	1.065847053	0.955399205
1.055853324	1.066013595	0.955398127
1.054861692	1.066180137	0.955397053
1.052537523	1.066346679	0.955395012
1.059276775	1.066513222	0.955392876
1.053904227	1.066679764	0.955390513
1.053920171	1.066846306	0.955388813
1.04830551	1.067012848	0.955386587
1.053952062	1.06717939	0.955384362
1.053968008	1.067345932	0.955381583
1.056333894	1.067512474	0.955379361
1.056349942	1.067679016	0.95537714
1.059405902	1.067845558	0.955375153
1.059422045	1.0680121	0.955373168
1.05639809	1.068178642	0.955371484
1.061489439	1.068345184	0.955369801
1.056430191	1.068511726	0.955367821
1.061521813	1.068678268	0.955366341
1.059163978	1.06884481	0.955364364
1.056478348	1.069011352	0.95536289
1.059196251	1.069177894	0.955361185
1.059212389	1.069344436	0.955359218
1.056526509	1.069510978	0.95535752
1.057216639	1.06967752	0.955355826
1.063675145	1.069844063	0.955353868
1.066416317	1.070010605	0.955351979
1.067114868	1.070177147	0.955350725
1.064404899	1.070343689	0.955349743
1.070220129	1.070510231	0.95534883
1.079515993	1.070676773	0.95534765
1.079532504	1.070843315	0.955347042
1.079549016	1.071009857	0.955347349
1.079565528	1.071176399	0.955347654
1.076818386	1.071176399	0.955347959
1.076834809	1.071176399	0.955348264
1.066238171	1.071176399	0.955348298
1.069325415	1.071176399	0.955348331
1.078609724	1.071176399	0.955347319

1.077590011	1.071176399	0.955346609
1.068008463	1.071176399	0.955346812
1.065296989	1.071176399	0.955346913
1.065313227	1.071176399	0.95534607
1.0643077	1.071176399	0.95534496
1.064323928	1.071176399	0.955343851
1.064340155	1.071176399	0.955342641
1.068447521	1.071176399	0.955341434
1.063691956	1.071176399	0.955340227
1.063708176	1.071176399	0.955339424
1.061004899	1.071176399	0.955338152
1.063059876	1.071176399	0.955336881
1.069894711	1.071176399	0.955335344
1.069910997	1.071176399	0.955334008
1.069927284	1.071176399	0.955333344
1.068577685	1.071176399	0.955332682
1.061781436	1.071176399	0.955332019
1.057394126	1.071176399	0.955331223
1.057410211	1.071176399	0.955329757
1.057426297	1.071176399	0.955327859
1.0584559	1.071176399	0.955325962
1.058472017	1.071176399	0.955324068
1.058488135	1.071176399	0.955322275
1.058504254	1.071176399	0.955320485
1.062930782	1.071176399	0.955318696
1.062946992	1.071176399	0.955316909
1.065686761	1.071176399	0.955315558
1.073905016	1.071176399	0.955314208
1.078396315	1.071176399	0.955313127
1.073937716	1.071176399	0.955312854
1.080160376	1.071176399	0.955313024
1.080176911	1.071176399	0.955312754
1.073986769	1.071176399	0.955313095
1.07400312	1.071176399	0.955313436
1.074019472	1.071176399	0.955313166
1.080590314	1.071176399	0.955312897
1.080606865	1.071176399	0.955312628
1.077853381	1.071176399	0.955313003
1.069293196	1.071176399	0.955313378
1.068626669	1.071176399	0.955313479
1.066936985	1.071176399	0.955312734
1.06695324	1.071176399	0.955311922
1.06662834	1.071176399	0.955310943
1.067326556	1.071176399	0.955309965

1.067342815	1.071176399	0.955308954
1.066677096	1.071176399	0.955308011
1.067375333	1.071176399	0.955307068
1.067391593	1.071176399	0.955306058
1.068773122	1.071176399	0.955305115
1.067424112	1.071176399	0.955304171
1.067440371	1.071176399	0.955303361
1.066774613	1.071176399	0.955302416
1.066450171	1.071176399	0.95530147
1.066466421	1.071176399	0.955300456
1.066482672	1.071176399	0.955299409
1.066498922	1.071176399	0.955298363
1.066515173	1.071176399	0.955297317
1.068578448	1.071176399	0.955296273
1.066547675	1.071176399	0.95529523
1.068610991	1.071176399	0.95529439
1.072730369	1.071176399	0.955293349
1.07652397	1.071176399	0.95529251
1.076540385	1.071176399	0.955292077
1.076556799	1.071176399	0.955292017
1.076573214	1.071176399	0.955291958
1.07658963	1.071176399	0.9552919
1.069407746	1.071176399	0.955291845
1.069424027	1.071176399	0.955291791
1.069440307	1.071176399	0.955291029
1.070139786	1.071176399	0.955290269
1.070156075	1.071176399	0.955289508
1.063012171	1.071176399	0.955288813
1.06438977	1.071176399	0.955288116
1.070546901	1.071176399	0.955286713
1.064422227	1.071176399	0.955285442
1.066823967	1.071176399	0.955284774
1.066840221	1.071176399	0.9552835
1.066856475	1.071176399	0.955282458
1.07336676	1.071176399	0.955281416
1.066888983	1.071176399	0.955280373
1.066905238	1.071176399	0.955279969
1.073415779	1.071176399	0.955278926
1.079286188	1.071176399	0.955277884
1.073448459	1.071176399	0.955277482
1.0734648	1.071176399	0.955277656
1.079335699	1.071176399	0.955277254
1.079352203	1.071176399	0.955276853
1.076261748	1.071176399	0.955277029

1.070107378	1.071176399	0.955277205
1.070807209	1.071176399	0.955277075
1.070823506	1.071176399	0.955276339
1.070839804	1.071176399	0.955275672
1.069147625	1.071176399	0.955275005
1.069163902	1.071176399	0.955274338
1.070888699	1.071176399	0.955273504
1.069196458	1.071176399	0.955272671
1.069212736	1.071176399	0.955272006
1.069229014	1.071176399	0.955271175
1.066856221	1.071176399	0.955270344
1.072339108	1.071176399	0.955269515
1.072355428	1.071176399	0.955268451
1.062817995	1.071176399	0.955267927
1.07238807	1.071176399	0.955267403
1.074804958	1.071176399	0.955265941
1.067636263	1.071176399	0.955265422
1.067652525	1.071176399	0.955265143
1.067668787	1.071176399	0.955264158
1.067685049	1.071176399	0.955263177
1.067701312	1.071176399	0.955262198
1.067717574	1.071176399	0.955261222
1.067733837	1.071176399	0.955260249
1.072534973	1.071176399	0.955259278
1.072551297	1.071176399	0.955258308
1.071540654	1.071176399	0.955257811
1.071899317	1.071176399	0.955257315
1.071915631	1.071176399	0.955256719
1.073301747	1.071176399	0.955256158
1.068871698	1.071176399	0.955255598
1.069912694	1.071176399	0.955255173
1.06992898	1.071176399	0.95525431
1.069945266	1.071176399	0.955253549
1.069961553	1.071176399	0.955252788
1.068611923	1.071176399	0.955252026
1.068628195	1.071176399	0.955251265
1.070010414	1.071176399	0.955250368
1.070026701	1.071176399	0.955249472
1.071068591	1.071176399	0.955248711
1.076229704	1.071176399	0.955247951
1.07624611	1.071176399	0.955247293
1.067701481	1.071176399	0.955247142
1.07147602	1.071176399	0.955246992
1.062284608	1.071176399	0.955245999

1.064342015	1.071176399	0.955245378
1.068107922	1.071176399	0.955243854
1.068124189	1.071176399	0.955242532
1.068140455	1.071176399	0.955241582
1.064406928	1.071176399	0.955240633
1.064423157	1.071176399	0.955239686
1.066824898	1.071176399	0.95523836
1.069229777	1.071176399	0.955237023
1.069246055	1.071176399	0.955235911
1.069262334	1.071176399	0.955235025
1.069278612	1.071176399	0.955234129
1.067588241	1.071176399	0.955233224
1.064877045	1.071176399	0.95523231
1.067620764	1.071176399	0.955231216
1.067637025	1.071176399	0.955229842
1.067653287	1.071176399	0.955228725
1.064941982	1.071176399	0.955227594
1.064958217	1.071176399	0.955226448
1.064974452	1.071176399	0.95522502
1.064990687	1.071176399	0.955223577
1.066710871	1.071176399	0.95522212
1.075967611	1.071176399	0.955220651
1.07598401	1.071176399	0.955219336
1.06675963	1.071176399	0.955218919
1.069506534	1.071176399	0.955218488
1.069181536	1.071176399	0.955217135
1.069197814	1.071176399	0.955216038
1.069214092	1.071176399	0.955214897
1.06923037	1.071176399	0.955213745
1.069246649	1.071176399	0.955212583
1.069262927	1.071176399	0.95521141
1.069279206	1.071176399	0.955210227
1.069295485	1.071176399	0.955209034
1.075820638	1.071176399	0.955207831
1.075837033	1.071176399	0.955206617
1.074822266	1.071176399	0.955206034
1.074838636	1.071176399	0.955205441
1.074855006	1.071176399	0.955204736
1.074871377	1.071176399	0.955204021
1.076262858	1.071176399	0.955203293
1.076968432	1.071176399	0.955202554
1.076984859	1.071176399	0.955201938
1.077001286	1.071176399	0.955201378
1.077017714	1.071176399	0.955200805

1.077034143	1.071176399	0.955200219
1.083996154	1.071176399	0.955199621
1.084012842	1.071176399	0.955199009
1.086135854	1.071176399	0.95519907
1.079172403	1.071176399	0.955199118
1.090411708	1.071176399	0.955199362
1.079205401	1.071176399	0.955198909
1.071313472	1.071176399	0.95519955
1.071329777	1.071176399	0.955199075
1.071346082	1.071176399	0.955197811
1.071362387	1.071176399	0.955196538
1.071378692	1.071176399	0.955195256
1.068661247	1.071176399	0.955193964
1.06867752	1.071176399	0.955192663
1.08072438	1.071176399	0.955191084
1.068710065	1.071176399	0.955189496
1.076952176	1.071176399	0.955189082
1.086370318	1.071176399	0.955187473
1.086387102	1.071176399	0.955186665
1.07459389	1.071176399	0.955186773
1.081171454	1.071176399	0.955186871
1.081188027	1.071176399	0.955185797
1.081204602	1.071176399	0.955185359
1.081221177	1.071176399	0.955184912
1.073303279	1.071176399	0.955184456
1.075379072	1.071176399	0.955183989
1.068889498	1.071176399	0.955182731
1.068905773	1.071176399	0.955181666
1.068922048	1.071176399	0.95517995
1.075444609	1.071176399	0.955178223
1.070320842	1.071176399	0.955176483
1.070337133	1.071176399	0.955175372
1.070353425	1.071176399	0.955173743
1.070369716	1.071176399	0.955172104
1.080014431	1.071176399	0.955170454
1.07933759	1.071176399	0.955168792
1.079354095	1.071176399	0.955168068
1.069751115	1.071176399	0.955167264
1.079040776	1.071176399	0.95516645
1.073205428	1.071176399	0.955164678
1.073221764	1.071176399	0.955163811
1.073238101	1.071176399	0.95516236
1.082233153	1.071176399	0.9551609
1.073270774	1.071176399	0.955159433

1.073287112	1.071176399	0.955158841
1.073303449	1.071176399	0.955157357
1.080559627	1.071176399	0.955155863
1.086169753	1.071176399	0.955154361
1.080592728	1.071176399	0.955153562
1.085851312	1.071176399	0.955153304
1.087630484	1.071176399	0.955152484
1.085884839	1.071176399	0.955152171
1.075477551	1.071176399	0.95515202
1.085918369	1.071176399	0.955151685
1.085935135	1.071176399	0.955150312
1.085951902	1.071176399	0.955149955
1.080378222	1.071176399	0.955149588
1.070077262	1.071176399	0.95514921
1.080411309	1.071176399	0.95514827
1.070109838	1.071176399	0.955146304
1.070126127	1.071176399	0.955145344
1.075625468	1.071176399	0.955143358
1.082565602	1.071176399	0.955141361
1.075658248	1.071176399	0.955139896
1.075674639	1.071176399	0.955139104
1.07569103	1.071176399	0.955137619
1.077774545	1.071176399	0.955136125
1.078136007	1.071176399	0.955134622
1.077807449	1.071176399	0.955133312
1.077823902	1.071176399	0.955132027
1.0781854	1.071176399	0.9551307
1.078201865	1.071176399	0.955129364
1.073059183	1.071176399	0.955128055
1.073075517	1.071176399	0.955126737
1.073434332	1.071176399	0.955124902
1.074823374	1.071176399	0.955123059
1.069703026	1.071176399	0.95512124
1.06767082	1.071176399	0.955119548
1.067687082	1.071176399	0.955117342
1.069751878	1.071176399	0.955114925
1.067719607	1.071301305	0.955112501
1.067735869	1.071426212	0.955110269
1.069117698	1.071551118	0.955107827
1.074611192	1.071676025	0.955105375
1.069150253	1.071800931	0.955103048
1.072586412	1.071925838	0.955101251
1.078448908	1.072050744	0.955098905
1.078465382	1.072175651	0.955096886

1.082299967	1.072300557	0.955095433
1.082316586	1.072425464	0.955093969
1.082333205	1.07255037	0.955092872
1.085852098	1.072675277	0.955091766
1.082366446	1.072800183	0.955090649
1.082383068	1.07292509	0.955089868
1.08590239	1.073049996	0.955088731
1.082416313	1.073174903	0.955087584
1.085935921	1.073299809	0.955086773
1.085952688	1.073424716	0.955085608
1.080725932	1.073549622	0.955084778
1.070761543	1.073674529	0.95508394
1.080759046	1.073799435	0.955082575
1.082167647	1.073924342	0.955080218
1.08218426	1.074049248	0.955078834
1.085350291	1.074174155	0.955077577
1.085367034	1.074299061	0.955076311
1.085383778	1.074423968	0.955075344
1.070191623	1.074548874	0.955074368
1.085417268	1.074673781	0.955073381
1.09393442	1.074798687	0.955070886
1.099631922	1.074923594	0.955069878
1.093968344	1.0750485	0.955069695
1.093985306	1.075173407	0.955070061
1.094002269	1.075298313	0.955069855
1.084815207	1.07542322	0.955069639
1.074775033	1.075548126	0.955069413
1.084848651	1.075673033	0.955068271
1.084865374	1.075797939	0.955066128
1.0793057	1.075922846	0.955064965
1.074840512	1.076047752	0.955063792
1.074856882	1.076172659	0.95506206
1.074873253	1.076297565	0.955059877
1.074889625	1.076422472	0.955057684
1.070110771	1.076547378	0.955055482
1.074578804	1.076672285	0.95505327
1.083597491	1.076797191	0.955050577
1.074611533	1.076922098	0.955048313
1.083630835	1.077047004	0.955046927
1.076707716	1.077171911	0.955044646
1.086120565	1.077296817	0.955043244
1.079850461	1.077421724	0.95504115
1.079866985	1.07754663	0.955039974
1.071625	1.077671537	0.95503817

1.079900033	1.077796443	0.955036356
1.080263557	1.07792135	0.955033721
1.074072448	1.078046256	0.95503189
1.080296636	1.078171163	0.955030085
1.081355528	1.078296069	0.955027659
1.08909684	1.078420976	0.955025838
1.08138869	1.078545882	0.955024111
1.089130597	1.078670789	0.955023137
1.09056589	1.078795695	0.955021395
1.090582807	1.078920602	0.955020407
1.091309835	1.079045508	0.955019553
1.091326769	1.079170415	0.955018693
1.091343703	1.079295321	0.955017896
1.091360637	1.079420228	0.955017101
1.093155382	1.079545134	0.955016308
1.093172341	1.079670041	0.955015516
1.0931893	1.079794947	0.955014901
1.093917812	1.079919854	0.955014285
1.087195037	1.08004476	0.955013671
1.090396909	1.080169667	0.955013128
1.091123762	1.080294573	0.955011923
1.079900636	1.08041948	0.955011033
1.079917161	1.080544386	0.955010214
1.079933687	1.080669293	0.955008291
1.071348544	1.080794199	0.955006371
1.078235912	1.080919106	0.955004454
1.078252378	1.081044012	0.955001694
1.078268846	1.081168919	0.954999615
1.085953648	1.081293825	0.954997541
1.078301782	1.081418732	0.954995471
1.075905265	1.081543638	0.954994161
1.081803444	1.081668545	0.9549921
1.085317329	1.081793451	0.954989804
1.083931667	1.081918358	0.954988091
1.083948353	1.082043264	0.954986725
1.085016697	1.082168171	0.954985225
1.097084977	1.082293077	0.954983729
1.085050158	1.082417984	0.954982338
1.085066889	1.08254289	0.954982137
1.087901154	1.082667797	0.954980751
1.091817981	1.082792703	0.954979366
1.101058751	1.08291761	0.954978261
1.100724128	1.083042516	0.954977543
1.100740899	1.083167423	0.954977735

1.10075767	1.083292329	0.954977895
1.091902697	1.083417236	0.954978056
1.088019043	1.083542142	0.954978218
1.088035887	1.083667049	0.95497751
1.082434062	1.083791955	0.954976419
1.082450686	1.083916862	0.95497533
1.082467311	1.084041768	0.954973689
1.082483937	1.084166675	0.954972051
1.082500563	1.084291581	0.954970415
1.08251719	1.084416488	0.954968782
1.082882981	1.084541394	0.954967152
1.078730156	1.084666301	0.954965525
1.081522144	1.084791207	0.954963935
1.087497967	1.084916114	0.954961937
1.093189653	1.08504102	0.954960216
1.093206612	1.085165927	0.954959084
1.093223571	1.085290833	0.954958514
1.098925759	1.08541574	0.954957946
1.089704952	1.085540646	0.954957381
1.089721848	1.085665553	0.954957379
1.09897635	1.085790459	0.954956468
1.103555849	1.085915366	0.954955561
1.103572406	1.086040272	0.954955567
1.103588962	1.086165179	0.954956026
1.102560706	1.086290085	0.954956488
1.094088227	1.086414992	0.954956953
1.085602646	1.086539898	0.954957318
1.084917116	1.086664805	0.95495685
1.085636154	1.086789711	0.954955548
1.088474022	1.086914618	0.95495418
1.085669664	1.087039524	0.954952885
1.08568642	1.087164431	0.954951871
1.083949135	1.087289337	0.954950581
1.083965821	1.087414244	0.954949294
1.082235835	1.08753915	0.954947838
1.083999196	1.087664057	0.954946386
1.082269068	1.087788963	0.954944767
1.082285686	1.08791387	0.954943323
1.082302304	1.088038776	0.954941712
1.081970241	1.088163683	0.954940105
1.082335543	1.088288589	0.954938501
1.082352163	1.088413496	0.954936866
1.084466556	1.088538402	0.954935269
1.089064227	1.088663309	0.954933675

1.089081105	1.088788215	0.954932291
1.089097983	1.088913122	0.954931359
1.089114861	1.089038028	0.954930429
1.09232655	1.089162935	0.954929501
1.0923435	1.089287841	0.954928576
1.09236045	1.089412748	0.954927968
1.089891449	1.089537654	0.954927366
1.08990835	1.089662561	0.954926769
1.083932623	1.089787467	0.954925932
1.080810618	1.089912374	0.9549251
1.083965995	1.09003728	0.954923685
1.080843738	1.090162187	0.954921965
1.083649679	1.090287093	0.954920559
1.079835659	1.090412	0.954918848
1.079852182	1.090536906	0.954917416
1.082651443	1.090661813	0.95491561
1.082668076	1.090786719	0.954913807
1.082684709	1.090911626	0.954912284
1.082003625	1.091036532	0.954910764
1.080629107	1.091161439	0.954909247
1.08064566	1.091286345	0.954907666
1.079967859	1.091411252	0.954905949
1.079984387	1.091536158	0.954904236
1.080000915	1.091661065	0.954902457
1.083150124	1.091785971	0.954900681
1.078302897	1.091910878	0.954898908
1.078319366	1.092035784	0.954897446
1.078335836	1.092160691	0.954895509
1.081125361	1.092285597	0.954893576
1.090989657	1.092410504	0.954891646
1.091006584	1.09253541	0.954889994
1.104447751	1.092660317	0.954889316
1.092818184	1.092785223	0.954888642
1.09283514	1.09291013	0.954889294
1.092852096	1.093035036	0.954888803
1.096071271	1.093159943	0.954888318
1.088626562	1.093284849	0.954887837
1.085469602	1.093409756	0.954887676
1.086893969	1.093534662	0.954886784
1.086206363	1.093659569	0.954885585
1.08622314	1.093784475	0.954884529
1.086239918	1.093909382	0.954883408
1.087314065	1.094034288	0.954882293
1.089098334	1.094159195	0.954881182

1.090888718	1.094284101	0.95488018
1.091260535	1.094409008	0.954879356
1.094478707	1.094533914	0.95487871
1.094495669	1.094658821	0.954878102
1.094512631	1.094783727	0.954877811
1.099852335	1.094908634	0.954877523
1.099869156	1.09503354	0.954877238
1.105465094	1.095158447	0.954877478
1.10548146	1.095283353	0.95487772
1.110216447	1.09540826	0.954878513
1.115053065	1.095533166	0.954879306
1.120482219	1.095658073	0.954880566
1.11814136	1.095782979	0.954882301
1.118155633	1.095907886	0.954884572
1.118169902	1.096032792	0.954886612
1.109979948	1.096157699	0.954888653
1.123654076	1.096282605	0.954890696
1.123667197	1.096407512	0.954891932
1.110027243	1.096532418	0.954894516
1.100792518	1.096657325	0.954897101
1.100809285	1.096782231	0.954898344
1.100826052	1.096907138	0.954898678
1.100842817	1.097032044	0.954899015
1.100859581	1.097156951	0.954899354
1.100172213	1.097281857	0.954899695
1.111776037	1.097406764	0.954900039
1.104399095	1.09753167	0.954900318
1.104415573	1.097656577	0.95490174
1.108521312	1.097781483	0.954902436
1.109870085	1.09790639	0.954903134
1.109885869	1.098031296	0.954904237
1.109901651	1.098156203	0.954905473
1.114125664	1.098281109	0.954906712
1.114140724	1.098406016	0.954907953
1.114155781	1.098530922	0.954909608
1.114486177	1.098655829	0.954911262
1.11450117	1.098780735	0.954912917
1.10999629	1.098905642	0.954914602
1.110999367	1.099030548	0.954916289
1.109031034	1.099155455	0.954917529
1.109046943	1.099280361	0.954918867
1.099398822	1.099405268	0.954920008
1.092666562	1.099530174	0.954921149
1.099432507	1.099655081	0.954921338

1.105384004	1.099779987	0.954920863
1.099112984	1.099904894	0.954921055
1.09273438	1.1000298	0.954921832
1.092751335	1.100154707	0.954921991
1.09276829	1.100279613	0.954921523
1.099180404	1.10040452	0.954921059
1.092446353	1.100529426	0.954920599
1.098506473	1.100654333	0.954920771
1.098523354	1.100779239	0.954920283
1.091075708	1.100904146	0.954920394
1.091092637	1.101029052	0.95492051
1.088627529	1.101153959	0.954919895
1.088644392	1.101278865	0.954919286
1.091142985	1.101403772	0.954918439
1.091159915	1.101528678	0.954917597
1.091176846	1.101653585	0.954917004
1.097594513	1.101778491	0.954916417
1.091210709	1.101903398	0.954915833
1.097628338	1.102028304	0.954915884
1.103623967	1.102153211	0.954915309
1.108089873	1.102278117	0.95491537
1.112392669	1.102403024	0.954916025
1.1177128	1.10252793	0.954917121
1.11772716	1.102652837	0.954918644
1.115601696	1.102777743	0.954920692
1.111481962	1.10290265	0.954922743
1.111497488	1.103027556	0.954924587
1.111513012	1.103152463	0.954926028
1.115350156	1.103277369	0.954927474
1.115364984	1.103402276	0.954928923
1.11537981	1.103527182	0.954930752
1.108601533	1.103652089	0.954932584
1.108617504	1.103776995	0.954934419
1.10626503	1.103901902	0.954935589
1.095056161	1.104026808	0.954936763
1.09507312	1.104151715	0.954937708
1.095090079	1.104276621	0.954937552
1.096174097	1.104401528	0.954937402
1.093700402	1.104526434	0.954937257
1.090517781	1.104651341	0.954937223
1.090534698	1.104776247	0.954936948
1.090551615	1.104901154	0.954936364
1.090568533	1.10502606	0.954935784
1.092717778	1.105150967	0.954935208

1.098067864	1.105275873	0.954934637
1.103359286	1.10540078	0.95493428
1.108505827	1.105525686	0.954934452
1.111171622	1.105650593	0.954935146
1.116876301	1.105775499	0.954936349
1.121603186	1.105900406	0.954937816
1.121616738	1.106025312	0.954939844
1.116919878	1.106150219	0.954942338
1.116934398	1.106275125	0.954944834
1.121657375	1.106400032	0.954946868
1.121670915	1.106524938	0.954948905
1.121684453	1.106649845	0.954951407
1.125016902	1.106774751	0.954953911
1.125029741	1.106899658	0.954956417
1.128702977	1.107024564	0.954959254
1.119428201	1.107149471	0.954962092
1.119442208	1.107274377	0.954965294
1.119456212	1.107399284	0.954967582
1.113975931	1.10752419	0.954969872
1.113991019	1.107649097	0.954972163
1.114006104	1.107774003	0.954973914
1.1071247	1.10789891	0.954975667
1.09666539	1.108023816	0.954977422
1.099164516	1.108148723	0.9549785
1.09918137	1.108273629	0.954978548
1.099198223	1.108398536	0.954978842
1.096733133	1.108523442	0.954979139
1.099231927	1.108648349	0.954979439
1.099248778	1.108773255	0.954979496
1.099265628	1.108898162	0.9549798
1.099282477	1.109023068	0.954980105
1.100005878	1.109147975	0.954980411
1.100022691	1.109272881	0.954980718
1.100391723	1.109272881	0.954981097
1.100408514	1.109272881	0.954981475
1.100425304	1.109272881	0.95498189
1.100442093	1.109272881	0.954982307
1.100458882	1.109272881	0.954982727
1.103972002	1.109272881	0.954983148
1.103988521	1.109272881	0.954983572
1.108106834	1.109272881	0.954984341
1.10879378	1.109272881	0.954985113
1.110469067	1.109272881	0.954986291
1.110484758	1.109272881	0.954987536

1.110500446	1.109272881	0.954988947
1.112147808	1.109272881	0.954990359
1.112163222	1.109272881	0.954991771
1.112822777	1.109272881	0.954993344
1.112838073	1.109272881	0.954994916
1.112853367	1.109272881	0.954996552
1.112868657	1.109272881	0.954998187
1.112883946	1.109272881	0.954999822
1.115113907	1.109272881	0.955001457
1.115128781	1.109272881	0.955003093
1.108666317	1.109272881	0.955004947
1.106996032	1.109272881	0.9550068
1.107012218	1.109272881	0.955008017
1.102547097	1.109272881	0.955009069
1.10256374	1.109272881	0.955010125
1.103972174	1.109272881	0.955010742
1.104680277	1.109272881	0.955011362
1.107769836	1.109272881	0.955012122
1.104713174	1.109272881	0.955012952
1.102646937	1.109272881	0.955014087
1.102314557	1.109272881	0.955014921
1.102331219	1.109272881	0.955015549
1.104087775	1.109272881	0.955016142
1.104795387	1.109272881	0.955016733
1.108889573	1.109272881	0.955017493
1.112194202	1.109272881	0.955018319
1.112209608	1.109272881	0.955019546
1.112868817	1.109272881	0.955021094
1.112884105	1.109272881	0.95502264
1.11289939	1.109272881	0.955024248
1.112914673	1.109272881	0.955025854
1.112929953	1.109272881	0.955027455
1.108682861	1.109272881	0.955029054
1.107689965	1.109272881	0.955030651
1.107706061	1.109272881	0.955031828
1.107722154	1.109272881	0.955032906
1.103625691	1.109272881	0.955033984
1.103642242	1.109272881	0.955035062
1.104351712	1.109272881	0.955035737
1.104368195	1.109272881	0.955036412
1.105075021	1.109272881	0.955037157
1.105091429	1.109272881	0.955037904
1.104417632	1.109272881	0.955038718
1.102697013	1.109272881	0.955039534

1.101664295	1.109272881	0.955040283
1.101681005	1.109272881	0.955040861
1.100995752	1.109272881	0.955041337
1.099955961	1.109272881	0.955041814
1.099972776	1.109272881	0.955042224
1.09998959	1.109272881	0.955042532
1.100006404	1.109272881	0.95504284
1.100023216	1.109272881	0.955043149
1.100040028	1.109272881	0.95504346
1.100056839	1.109272881	0.955043772
1.100778457	1.109272881	0.955044084
1.100795225	1.109272881	0.955044397
1.100811992	1.109272881	0.955044779
1.10118043	1.109272881	0.955045159
1.101197173	1.109272881	0.955045537
1.101213915	1.109272881	0.955045947
1.101230655	1.109272881	0.955046354
1.101247395	1.109272881	0.95504676
1.100912574	1.109272881	0.955047164
1.100929334	1.109272881	0.955047567
1.100594114	1.109272881	0.955047936
1.100258496	1.109272881	0.955048304
1.100275295	1.109272881	0.955048639
1.09993932	1.109272881	0.955048939
1.099956136	1.109272881	0.955049241
1.099972951	1.109272881	0.955049509
1.099989765	1.109272881	0.955049778
1.100006579	1.109272881	0.95505005
1.100023391	1.109272881	0.955050323
1.100040203	1.109272881	0.955050599
1.102165065	1.109272881	0.955050876
1.10426962	1.109272881	0.955051156
1.10428611	1.109272881	0.955051646
1.104302599	1.109272881	0.955052344
1.104319086	1.109272881	0.955053045
1.104335572	1.109272881	0.955053748
1.101915426	1.109272881	0.955054452
1.099469347	1.109272881	0.955055157
1.099486187	1.109272881	0.955055623
1.099503026	1.109272881	0.955055849
1.099519864	1.109272881	0.955056076
1.099536702	1.109272881	0.955056304
1.100259109	1.109272881	0.955056532
1.104121735	1.109272881	0.95505676

1.107899376	1.109272881	0.955057049
1.109256116	1.109272881	0.955057706
1.109936329	1.109272881	0.955058725
1.112885379	1.109272881	0.955059867
1.116659846	1.109272881	0.955061065
1.117585378	1.109272881	0.955062541
1.12713697	1.109272881	0.955064376
1.12714938	1.109272881	0.95506629
1.126376329	1.109272881	0.955069132
1.126388891	1.109272881	0.955071959
1.12640145	1.109272881	0.955074698
1.111716926	1.109272881	0.955077423
1.105026298	1.109272881	0.955080136
1.104006329	1.109272881	0.955081389
1.102282358	1.109272881	0.955081969
1.101247743	1.109272881	0.955082434
1.100560902	1.109272881	0.955082717
1.099872835	1.109272881	0.955082884
1.099889655	1.109272881	0.955082971
1.099906473	1.109272881	0.955082976
1.100979956	1.109272881	0.955082968
1.104138412	1.109272881	0.955082948
1.106900565	1.109272881	0.95508302
1.105207177	1.109272881	0.955083389
1.105223572	1.109272881	0.955084016
1.10796422	1.109272881	0.955084463
1.107980279	1.109272881	0.955084896
1.107996335	1.109272881	0.955085585
1.105632249	1.109272881	0.95508626
1.10322931	1.109272881	0.955086923
1.10254883	1.109272881	0.955087337
1.102216296	1.109272881	0.955087501
1.102232966	1.109272881	0.955087583
1.101548861	1.109272881	0.95508762
1.101565579	1.109272881	0.955087643
1.101582295	1.109272881	0.955087587
1.101599011	1.109272881	0.955087519
1.102316293	1.109272881	0.955087438
1.102332955	1.109272881	0.955087346
1.103047368	1.109272881	0.95508731
1.10306397	1.109272881	0.955087262
1.10446817	1.109272881	0.95508727
1.106202711	1.109272881	0.955087266
1.108252529	1.109272881	0.955087386

1.111251593	1.109272881	0.955087664
1.118457789	1.109272881	0.95508813
1.118471996	1.109272881	0.955088877
1.126339217	1.109272881	0.955090321
1.129438674	1.109272881	0.955091752
1.131427877	1.109272881	0.955093946
1.13143948	1.109272881	0.955096432
1.13145108	1.109272881	0.955099101
1.131462679	1.109272881	0.955101759
1.131474275	1.109272881	0.955104407
1.118287589	1.109272881	0.955107044
1.118301832	1.109272881	0.955109671
1.108124878	1.109272881	0.955110988
1.107804265	1.109272881	0.955112296
1.105436705	1.109272881	0.955112593
1.105453074	1.109272881	0.955112847
1.108524975	1.109272881	0.955112857
1.111189877	1.109272881	0.955112858
1.111205452	1.109272881	0.95511315
1.111221024	1.109272881	0.955113693
1.111236594	1.109272881	0.955114227
1.111252161	1.109272881	0.95511475
1.108620831	1.109272881	0.955115264
1.105240305	1.109272881	0.955115768
1.108317178	1.109272881	0.955116002
1.10423818	1.109272881	0.955115891
1.104254674	1.109272881	0.955116071
1.104271166	1.109272881	0.955115837
1.104633404	1.109272881	0.955115592
1.106024672	1.109272881	0.955115335
1.106040975	1.109272881	0.955115101
1.106057276	1.109272881	0.955114991
1.106073575	1.109272881	0.95511487
1.106089872	1.109272881	0.955114738
1.106106167	1.109272881	0.955114595
1.106122461	1.109272881	0.955114442
1.106138752	1.109272881	0.95511428
1.104090268	1.109272881	0.955114108
1.10445247	1.109272881	0.955113925
1.104468942	1.109272881	0.95511353
1.106884872	1.109272881	0.955113158
1.106901071	1.109272881	0.955112777
1.107932597	1.109272881	0.955112622
1.109621581	1.109272881	0.955112459

1.109637403	1.109272881	0.955112386
1.109653222	1.109272881	0.955112468
1.109669039	1.109272881	0.95511254
1.109684854	1.109272881	0.955112603
1.109700666	1.109272881	0.955112657
1.10838179	1.109272881	0.955112702
1.10533922	1.109272881	0.955112737
1.105355599	1.109272881	0.955112632
1.104682933	1.109272881	0.955112216
1.103660861	1.109272881	0.955111789
1.102981896	1.109272881	0.955111283
1.102998504	1.109272881	0.955110662
1.10301511	1.109272881	0.955109961
1.101983924	1.109272881	0.955109246
1.100596037	1.109272881	0.955108519
1.100612816	1.109272881	0.955107677
1.102383452	1.109272881	0.955106685
1.100646371	1.109272881	0.955105681
1.100663148	1.109272881	0.955104839
1.101382751	1.109272881	0.955103812
1.103147479	1.109272881	0.955102774
1.104205532	1.109272881	0.955101794
1.106285034	1.109272881	0.955100975
1.111640662	1.109272881	0.955100246
1.111656162	1.109272881	0.955099708
1.109700831	1.109272881	0.955099682
1.10770824	1.109272881	0.955099645
1.107724334	1.109272881	0.955099401
1.107063542	1.109272881	0.955098946
1.107079719	1.109272881	0.955098479
1.107095893	1.109272881	0.955097932
1.109130843	1.109272881	0.955097372
1.109146737	1.109272881	0.9550968
1.10916263	1.109272881	0.955096417
1.111159613	1.109272881	0.955096024
1.109194408	1.109272881	0.955095622
1.111517135	1.109272881	0.955095404
1.112504293	1.109272881	0.95509498
1.115057485	1.109272881	0.955094773
1.115072369	1.109272881	0.955094651
1.112550341	1.109272881	0.95509477
1.111594712	1.109272881	0.955094879
1.108637714	1.109272881	0.955094725
1.105601252	1.109272881	0.955094465

1.104929663	1.109272881	0.955093899
1.104601434	1.109272881	0.955093021
1.10392605	1.109272881	0.955092064
1.103942573	1.109272881	0.955091063
1.103959095	1.109272881	0.955089982
1.104321576	1.109272881	0.955088889
1.105028179	1.109272881	0.955087783
1.105388695	1.109272881	0.9550867
1.106772772	1.109272881	0.955085675
1.107467343	1.109272881	0.955084673
1.107483468	1.109272881	0.955083797
1.107837512	1.109272881	0.955082976
1.107853588	1.109272881	0.955082145
1.109543343	1.109272881	0.955081334
1.107885734	1.109272881	0.955080511
1.107901803	1.109272881	0.955079841
1.109590837	1.109272881	0.955078993
1.109273976	1.109272881	0.955078133
1.110613623	1.109272881	0.955077424
1.111935278	1.109272881	0.95507667
1.113556532	1.109272881	0.955076034
1.113571696	1.109272881	0.955075514
1.113586858	1.109272881	0.955075141
1.113602017	1.109272881	0.955074757
1.113617174	1.109272881	0.955074362
1.113632327	1.109272881	0.955073957
1.113964849	1.109272881	0.955073541
1.113979939	1.109272881	0.955073113
1.112397473	1.109272881	0.955072706
1.118018064	1.109272881	0.955072287
1.118032362	1.109272881	0.955071698
1.118046657	1.109272881	0.95507165
1.114998616	1.109272881	0.955071589
1.112795683	1.109272881	0.955071514
1.117184257	1.109272881	0.955071124
1.117198724	1.109272881	0.955070502
1.117213188	1.109272881	0.955070299
1.120197293	1.109272881	0.955070082
1.120211139	1.109272881	0.955069852
1.117256562	1.109272881	0.955069901
1.117271014	1.109272881	0.955069938
1.117285463	1.109272881	0.95506967
1.122827207	1.109272881	0.955069391
1.129951626	1.109272881	0.955069099

1.134300389	1.109372807	0.95506934
1.136157597	1.109472732	0.95507027
1.13593995	1.109572657	0.955071614
1.135950851	1.109672582	0.955073126
1.135961751	1.109772508	0.955074602
1.135972649	1.109872433	0.955076064
1.135983546	1.109972358	0.955077513
1.134378268	1.110072283	0.955078947
1.127262955	1.110172209	0.955080366
1.117458628	1.110272134	0.955081612
1.116253714	1.110372059	0.955082141
1.116268366	1.110471984	0.955081689
1.116283015	1.110571909	0.955081104
1.112197733	1.110671835	0.955080507
1.109258845	1.11077176	0.955079898
1.114461339	1.110871685	0.955078874
1.114476336	1.11097161	0.955077548
1.118771302	1.111071536	0.95507672
1.118785445	1.111171461	0.955075881
1.118799585	1.111271386	0.955075452
1.114221154	1.111371311	0.955075013
1.108702477	1.111471237	0.955074564
1.108718433	1.111571162	0.955073652
1.108734388	1.111671087	0.955072184
1.10875034	1.111771012	0.955070707
1.108766289	1.111870937	0.955069219
1.106757486	1.111970863	0.955067722
1.106773701	1.112070788	0.955066213
1.107129332	1.112170713	0.955064496
1.105094762	1.112270638	0.955062769
1.107161667	1.112370564	0.955061065
1.108191017	1.112470489	0.955059147
1.105143975	1.112570414	0.95505742
1.108223071	1.112670339	0.955055782
1.112520684	1.112770265	0.955053831
1.113496715	1.11287019	0.955052172
1.114461495	1.112970115	0.955050924
1.113527063	1.11307004	0.955049761
1.114491487	1.113169966	0.955048682
1.118785592	1.113269891	0.955047499
1.12282783	1.113369816	0.9550464
1.11881387	1.113469741	0.955045715
1.118828004	1.113569666	0.955045416
1.113618042	1.113669592	0.955044712

1.112351826	1.113769517	0.955043998
1.113329761	1.113869442	0.955042761
1.11768901	1.113969367	0.955041389
1.122920831	1.114069293	0.955040103
1.118317999	1.114169218	0.955039237
1.118332236	1.114269143	0.955038876
1.118346469	1.114369068	0.955038053
1.126754232	1.114468994	0.955037222
1.126766718	1.114568919	0.955036382
1.128839893	1.114668844	0.955036358
1.128851971	1.114768769	0.955036325
1.128864046	1.114868694	0.955036485
1.127337483	1.11496862	0.955036634
1.127349853	1.115068545	0.955036774
1.12736222	1.11516847	0.955036752
1.123079945	1.115268395	0.955036719
1.123093186	1.115368321	0.955036677
1.123106425	1.115468246	0.955036202
1.127411665	1.115568171	0.955035719
1.124505758	1.115668096	0.955035227
1.120017965	1.115768022	0.95503515
1.119739821	1.115867947	0.955034777
1.119753763	1.115967872	0.955033952
1.119767702	1.116067797	0.95503309
1.119781638	1.116167723	0.955032219
1.119795571	1.116267648	0.955031341
1.119809502	1.116367573	0.955030454
1.119823429	1.116467498	0.955029558
1.119837354	1.116567423	0.955028652
1.119851276	1.116667349	0.955027736
1.123000576	1.116767274	0.955026811
1.121891668	1.116867199	0.955025877
1.125748161	1.116967124	0.955025244
1.129881481	1.11706705	0.955024494
1.133293375	1.117166975	0.955024114
1.135635289	1.1172669	0.955024131
1.13564623	1.117366825	0.955024474
1.139936421	1.117466751	0.955025037
1.139947077	1.117566676	0.955025591
1.135679043	1.117666601	0.955026558
1.132647563	1.117766526	0.955027515
1.133843849	1.117866451	0.955028042
1.132670348	1.117966377	0.955028259
1.132681738	1.118066302	0.955028582

1.130260492	1.118166227	0.955028779
1.130272304	1.118266152	0.955028965
1.132954415	1.118366078	0.955028902
1.130295922	1.118466003	0.955028829
1.13153434	1.118565928	0.955029009
1.131545924	1.118665853	0.955028916
1.135799247	1.118765779	0.955028935
1.134888845	1.118865704	0.955028945
1.134899889	1.118965629	0.955029364
1.130613796	1.119065554	0.955029682
1.130625544	1.11916548	0.955029988
1.126039687	1.119265405	0.955029873
1.122509183	1.11936533	0.95502976
1.123908633	1.119465255	0.955029196
1.123921701	1.11956518	0.955028286
1.123934766	1.119665106	0.955027515
1.119405142	1.119765031	0.955026746
1.119419153	1.119864956	0.955025978
1.119433162	1.119964881	0.955024766
1.123987002	1.120064807	0.955023556
1.12667998	1.120164732	0.95502235
1.126692481	1.120264657	0.955021594
1.130272427	1.120364582	0.955021105
1.130284237	1.120464508	0.95502062
1.124867658	1.120564433	0.95502049
1.122120767	1.120664358	0.955020363
1.123804124	1.120764283	0.955019704
1.123817215	1.120864208	0.955018777
1.122161087	1.120964134	0.955018018
1.122174521	1.121064059	0.955017261
1.122187952	1.121163984	0.955016342
1.123869548	1.121263909	0.955015426
1.123882624	1.121363835	0.95501451
1.126580014	1.12146376	0.955013762
1.126592535	1.121563685	0.955013017
1.130178301	1.12166361	0.95501254
1.135439021	1.121763536	0.955012067
1.139734653	1.121863461	0.955011947
1.139745301	1.121963386	0.955012346
1.135930581	1.122063311	0.95501317
1.139320078	1.122163237	0.955013995
1.135952384	1.122263162	0.955014446
1.133170029	1.122363087	0.955015231
1.130024474	1.122463012	0.955015686

1.130036329	1.122562937	0.955015867
1.124868127	1.122662863	0.955015741
1.120116247	1.122762788	0.955015619
1.120130111	1.122862713	0.955014991
1.115638102	1.122962638	0.955013898
1.115652874	1.123062564	0.95501281
1.115667644	1.123162489	0.955011283
1.115682411	1.123262414	0.955009761
1.117229833	1.123362339	0.955008243
1.11724429	1.123462265	0.955006729
1.119935903	1.12356219	0.955005371
1.121391924	1.123662115	0.955004017
1.125545646	1.12376204	0.95500293
1.125558376	1.123861966	0.955001991
1.125571104	1.123961891	0.955001463
1.128961383	1.124061816	0.955000939
1.128973437	1.124161741	0.955000419
1.12898549	1.124261666	0.955000237
1.129751441	1.124361592	0.955000058
1.132693779	1.124461517	0.954999885
1.137786008	1.124561442	0.954999792
1.140705488	1.124661367	0.954999993
1.137807419	1.124761293	0.955000701
1.137818124	1.124861218	0.955001699
1.140963232	1.124961143	0.955002416
1.142585283	1.125061068	0.955003137
1.142596545	1.125160994	0.95500417
1.142607812	1.125260919	0.955005365
1.137871633	1.125360844	0.955006564
1.129381813	1.125460769	0.955007767
1.124429386	1.125560694	0.955008506
1.124442346	1.12566062	0.955008411
1.125265517	1.125760545	0.955007833
1.125278305	1.12586047	0.95500726
1.12448121	1.125960395	0.955006774
1.123121039	1.126060321	0.955006294
1.123134272	1.126160246	0.95500574
1.123974695	1.126260171	0.955005056
1.126668193	1.126360096	0.955004379
1.131233653	1.126460022	0.955003789
1.131245289	1.126559947	0.95500347
1.127487367	1.126659872	0.955003605
1.13151195	1.126759797	0.955003747
1.136452082	1.126859723	0.955003525

1.145103465	1.126959648	0.955003703
1.145693939	1.127059573	0.955004371
1.147329448	1.127159498	0.955005894
1.147346412	1.127259423	0.955007479
1.145736508	1.127359349	0.955009228
1.143588248	1.127459274	0.955010981
1.145764991	1.127559199	0.955012579
1.147414596	1.127659124	0.955013968
1.149903044	1.12775905	0.955015573
1.147448888	1.127858975	0.955017342
1.143899383	1.1279589	0.95501936
1.139724339	1.128058825	0.955021137
1.135219824	1.128158751	0.955022566
1.139745634	1.128258676	0.955023586
1.139756282	1.128358601	0.955024166
1.14448052	1.128458526	0.955025194
1.141809227	1.128558451	0.955026226
1.139564775	1.128658377	0.955027724
1.139575419	1.128758302	0.955028961
1.141842125	1.128858227	0.955029979
1.141853098	1.128958152	0.955030999
1.141864075	1.129058078	0.955032245
1.143540988	1.129158003	0.955033492
1.141886038	1.129257928	0.955034741
1.134889708	1.129357853	0.955036155
1.127314928	1.129457779	0.955037405
1.126543611	1.129557704	0.955037966
1.12655614	1.129657629	0.955037783
1.126568665	1.129757554	0.955037524
1.126581188	1.12985748	0.955037267
1.126330378	1.129957405	0.955037011
1.127129148	1.13005733	0.955036758
1.12475272	1.130157255	0.955036483
1.122563653	1.13025718	0.955036288
1.122577003	1.130357106	0.955035862
1.127954276	1.130457031	0.955035223
1.138650493	1.130556956	0.955034589
1.138661147	1.130656881	0.955034489
1.148352702	1.130756807	0.955035448
1.147605525	1.130856732	0.955036412
1.147623032	1.130956657	0.955038336
1.147640575	1.131056582	0.95504019
1.147658152	1.131156508	0.955042052
1.149747873	1.131256433	0.95504392

1.147693414	1.131356358	0.955045795
1.146025372	1.131456283	0.955047879
1.138085696	1.131556208	0.955049765
1.139660961	1.131656134	0.955051489
1.133755874	1.131756059	0.955052434
1.132100376	1.131855984	0.955053536
1.132111862	1.131955909	0.955054059
1.131152501	1.132055835	0.95505442
1.132134828	1.13215576	0.955054785
1.132146309	1.132255685	0.955055057
1.132157787	1.13235561	0.955055429
1.132169263	1.132455536	0.955055804
1.131210736	1.132555461	0.955056184
1.127191308	1.132655386	0.955056568
1.125623512	1.132755311	0.955056861
1.125636227	1.132855237	0.955056762
1.124572877	1.132955162	0.955056512
1.124585808	1.133055087	0.955056267
1.123779437	1.133155012	0.955055919
1.121270496	1.133254937	0.955055577
1.120131194	1.133354863	0.955055159
1.12214919	1.133454788	0.955054497
1.120158911	1.133554713	0.955053728
1.119589032	1.133654638	0.955053161
1.119603006	1.133754564	0.955052404
1.119616976	1.133854489	0.955051594
1.120504263	1.133954414	0.95505079
1.122510853	1.134054339	0.955049992
1.124183988	1.134154265	0.955049285
1.126079146	1.13425419	0.955048779
1.131176226	1.134354115	0.955048443
1.131187873	1.13445404	0.955048297
1.135231451	1.134553966	0.955048658
1.138640338	1.134653891	0.955049024
1.138650993	1.134753816	0.955049792
1.142983514	1.134853741	0.955050901
1.142994984	1.134953666	0.955052016
1.14300646	1.135053592	0.955053563
1.143017944	1.135153517	0.955055115
1.138480579	1.135253442	0.95505667
1.140055116	1.135353367	0.955058231
1.140964073	1.135453293	0.955059348
1.132524067	1.135553218	0.955060624
1.128588379	1.135653143	0.955061995

1.131824777	1.135753068	0.955062538
1.13183631	1.135852994	0.9550627
1.133284025	1.135952919	0.955063184
1.139012918	1.136052844	0.955063674
1.139023561	1.136152769	0.955064311
1.141963938	1.136252694	0.955065518
1.14197495	1.13635262	0.955066729
1.139948909	1.136452545	0.955068235
1.139959566	1.13655247	0.955069746
1.139970223	1.136652395	0.955071062
1.139980881	1.136752321	0.955072382
1.142262247	1.136852246	0.955073707
1.142744336	1.136952171	0.955075037
1.137776528	1.137052096	0.955076595
1.136203688	1.137152022	0.955078204
1.136214556	1.137251947	0.955079327
1.129800415	1.137351872	0.955080298
1.129812312	1.137451797	0.955081273
1.129824207	1.137551723	0.95508162
1.131801824	1.137651648	0.95508197
1.1325356	1.137751573	0.955082323
1.132547013	1.137851498	0.955082875
1.132558423	1.137951423	0.955083501
1.132569832	1.138051349	0.955084131
1.132581239	1.138151274	0.955084766
1.132592644	1.138251199	0.955085405
1.136105928	1.138351124	0.955086049
1.136116808	1.13845105	0.955086699
1.138832167	1.138550975	0.9550877
1.143924886	1.1386509	0.955088707
1.147163502	1.138750825	0.955089986
1.147180147	1.138850751	0.955091771
1.147196823	1.138950676	0.95509388
1.147213532	1.139050601	0.955095995
1.149191048	1.139150526	0.955098116
1.14563908	1.139250451	0.955100243
1.143756158	1.139350377	0.955102568
1.143029673	1.139450302	0.955104546
1.142801215	1.139550227	0.95510634
1.142812585	1.139650152	0.955108066
1.142823961	1.139750078	0.955109772
1.143075701	1.139850003	0.955111482
1.141679464	1.139949928	0.955113197
1.138097382	1.140049853	0.95511494

1.134225619	1.140149779	0.955116548
1.136084277	1.140249704	0.955117806
1.13812943	1.140349629	0.955118687
1.140825179	1.140449554	0.955119754
1.142676944	1.14054948	0.955121024
1.141526663	1.140649405	0.955122563
1.141537546	1.14074933	0.955124286
1.140868161	1.140849255	0.955125899
1.140204686	1.14094918	0.955127516
1.14021536	1.141049106	0.955129069
1.140226034	1.141149031	0.95513056
1.140236709	1.141248956	0.955132055
1.134359211	1.141348881	0.955133553
1.127501249	1.141448807	0.955135055
1.127513587	1.141548732	0.955135982
1.127525922	1.141648657	0.955136237
1.125165189	1.141748582	0.955136495
1.125177997	1.141848508	0.955136757
1.125190803	1.141948433	0.955136789
1.126794672	1.142048358	0.955136826
1.129371769	1.142148283	0.955136866
1.12963462	1.142248208	0.955137067
1.133768257	1.142348134	0.955137524
1.14289276	1.142448059	0.955138009
1.142904179	1.142547984	0.955138903
1.143649164	1.142647909	0.955140696
1.144972445	1.142747835	0.955142492
1.153301668	1.14284776	0.955144361
1.156060644	1.142947685	0.955146361
1.162392098	1.14304761	0.955149184
1.156130386	1.143147536	0.955152281
1.156165332	1.143147536	0.955156003
1.153452417	1.143147536	0.95515911
1.144256497	1.143147536	0.955162222
1.135298145	1.143147536	0.95516507
1.130050343	1.143147536	0.955167015
1.128806982	1.143147536	0.955168082
1.128819066	1.143147536	0.955168636
1.128577072	1.143147536	0.955169073
1.128334147	1.143147536	0.955169515
1.128090599	1.143147536	0.955169937
1.128102821	1.143147536	0.955170339
1.128370664	1.143147536	0.955170721
1.128637689	1.143147536	0.95517111

1.128649805	1.143147536	0.955171529
1.129168378	1.143147536	0.955171979
1.13018058	1.143147536	0.955172435
1.130932194	1.143147536	0.955172947
1.131188783	1.143147536	0.955173562
1.131200428	1.143147536	0.955174254
1.13121207	1.143147536	0.955174977
1.130978943	1.143147536	0.955175707
1.13123535	1.143147536	0.955176442
1.131246986	1.143147536	0.95517716
1.131258621	1.143147536	0.955177907
1.131513942	1.143147536	0.95517866
1.13152553	1.143147536	0.955179418
1.131537115	1.143147536	0.955180206
1.131305138	1.143147536	0.955180998
1.132044353	1.143147536	0.955181795
1.134891204	1.143147536	0.955182573
1.139428536	1.143147536	0.955183427
1.139439177	1.143147536	0.955184564
1.144635387	1.143147536	0.955186149
1.140579225	1.143147536	0.955187737
1.144660714	1.143147536	0.955189838
1.144673566	1.143147536	0.95519154
1.144686432	1.143147536	0.955193646
1.144699312	1.143147536	0.955195756
1.144712206	1.143147536	0.955197868
1.135462895	1.143147536	0.955199983
1.1304756	1.143147536	0.955202102
1.128480533	1.143147536	0.955203312
1.12849268	1.143147536	0.955204034
1.128504824	1.143147536	0.955204564
1.128516966	1.143147536	0.9552051
1.128529105	1.143147536	0.955205641
1.128795462	1.143147536	0.955206188
1.129813861	1.143147536	0.95520674
1.13322914	1.143147536	0.955207323
1.139184299	1.143147536	0.95520801
1.13919494	1.143147536	0.955209036
1.145739471	1.143147536	0.955210652
1.15309563	1.143147536	0.955212273
1.153125239	1.143147536	0.955214542
1.153154909	1.143147536	0.955217539
1.149446477	1.143147536	0.955220542
1.149467957	1.143147536	0.95522355

1.149489486	1.143147536	0.955226193
1.149511064	1.143147536	0.955228839
1.14953269	1.143147536	0.955231487
1.153334201	1.143147536	0.955234136
1.15468076	1.143147536	0.955236787
1.160818058	1.143147536	0.955239811
1.160856589	1.143147536	0.955242966
1.160895124	1.143147536	0.955246725
1.167088726	1.143147536	0.955250475
1.167121802	1.143147536	0.955254215
1.167154819	1.143147536	0.955258552
1.165757262	1.143147536	0.955262879
1.165792439	1.143147536	0.955267198
1.151827523	1.143147536	0.955271368
1.141213683	1.143147536	0.955275532
1.141224494	1.143147536	0.955278309
1.141235308	1.143147536	0.955280031
1.142871009	1.143147536	0.955281743
1.142882417	1.143147536	0.955283448
1.139248254	1.143147536	0.955285306
1.139258895	1.143147536	0.955287156
1.136573604	1.143147536	0.955288637
1.136584428	1.143147536	0.955290111
1.132878333	1.143147536	0.955291309
1.128964585	1.143147536	0.955292498
1.128976639	1.143147536	0.955293312
1.128735248	1.143147536	0.955293732
1.128237806	1.143147536	0.955294143
1.12825	1.143147536	0.95529452
1.129277122	1.143147536	0.955294839
1.130039602	1.143147536	0.955295149
1.131038306	1.143147536	0.95529555
1.131049977	1.143147536	0.955296015
1.131549362	1.143147536	0.955296569
1.135342825	1.143147536	0.955297113
1.135353805	1.143147536	0.955297697
1.141224607	1.143147536	0.955298646
1.14462331	1.143147536	0.955299586
1.144636121	1.143147536	0.955301094
1.153156301	1.143147536	0.955302927
1.14780332	1.143147536	0.95530475
1.135189345	1.143147536	0.955307402
1.135200346	1.143147536	0.955309518
1.135211346	1.143147536	0.955310381

1.130945284	1.143147536	0.955311237
1.130464807	1.143147536	0.955312086
1.130228982	1.143147536	0.955312506
1.129492683	1.143147536	0.955312869
1.129001179	1.143147536	0.955313201
1.129013226	1.143147536	0.95531345
1.129528539	1.143147536	0.95531364
1.129790747	1.143147536	0.955313822
1.131038731	1.143147536	0.955314046
1.133691006	1.143147536	0.955314286
1.141648073	1.143147536	0.955314638
1.148471895	1.143147536	0.955315241
1.148491211	1.143147536	0.955316617
1.149362259	1.143147536	0.955318654
1.149383549	1.143147536	0.955320682
1.150345246	1.143147536	0.955322783
1.149426272	1.143147536	0.955324874
1.148588421	1.143147536	0.955327048
1.140826522	1.143147536	0.955329119
1.140837265	1.143147536	0.955331096
1.133342394	1.143147536	0.955332296
1.133353674	1.143147536	0.955333486
1.133836789	1.143147536	0.955333925
1.133847991	1.143147536	0.955334355
1.136400865	1.143147536	0.955334821
1.136411709	1.143147536	0.955335277
1.138002646	1.143147536	0.955335973
1.143008553	1.143147536	0.95533666
1.143020038	1.143147536	0.955337492
1.143031529	1.143147536	0.955338806
1.143043027	1.143147536	0.955340109
1.140291711	1.143147536	0.955341401
1.145418683	1.143147536	0.95534268
1.145432465	1.143147536	0.955343674
1.153068081	1.143147536	0.955345159
1.164427367	1.143147536	0.955346632
1.164464162	1.143147536	0.955348842
1.147419321	1.143147536	0.955352157
1.147436459	1.143147536	0.955355462
1.138589391	1.143147536	0.955357077
1.147470837	1.143147536	0.95535868
1.155996346	1.143147536	0.955359401
1.1560311	1.143147536	0.955360985
1.142242331	1.143147536	0.955363395

1.134082828	1.143147536	0.955365794
1.134093992	1.143147536	0.955366823
1.142275686	1.143147536	0.955367037
1.147592235	1.143147536	0.95536724
1.161745805	1.143147536	0.955368239
1.170254897	1.143147536	0.955369751
1.161822729	1.143147536	0.955372646
1.166488097	1.143147536	0.95537637
1.150229474	1.143147536	0.955379252
1.150252741	1.143147536	0.955382584
1.150276063	1.143147536	0.955384306
1.150299437	1.143147536	0.955386021
1.150322866	1.143147536	0.955387731
1.147402839	1.143147536	0.955389433
1.145783357	1.143147536	0.955391127
1.143135677	1.143147536	0.955392523
1.143147237	1.143147536	0.955393747
1.136574337	1.143147536	0.955394697
1.141528817	1.143147536	0.955395635
1.144446233	1.143147536	0.955395913
1.144458857	1.143147536	0.955396667
1.137746467	1.143147536	0.955397696
1.132674501	1.143147536	0.955398715
1.13268589	1.143147536	0.955399062
1.131977075	1.143147536	0.955398898
1.131016051	1.143147536	0.955398724
1.131027727	1.143147536	0.95539847
1.130052257	1.143147536	0.955398111
1.130064107	1.143147536	0.955397745
1.130075955	1.143147536	0.955397272
1.131074408	1.143147536	0.95539679
1.133725318	1.143147536	0.9553963
1.136053889	1.143147536	0.955395896
1.136747379	1.143147536	0.955395741
1.136758184	1.143147536	0.955395804
1.136768987	1.143147536	0.955395924
1.136097427	1.143147536	0.955396034
1.136108308	1.143147536	0.955396133
1.135890822	1.143272442	0.955396154
1.135901729	1.143397349	0.955396163
1.136596378	1.143522255	0.955396137
1.135694647	1.143647162	0.955396098
1.135245367	1.143772068	0.955396115
1.133859541	1.143896975	0.95539603

1.133635617	1.144021881	0.955395887
1.134116837	1.144146788	0.955395593
1.134127996	1.144271694	0.955395263
1.132000507	1.144396601	0.955394965
1.13201201	1.144521507	0.955394654
1.131539287	1.144646414	0.95539412
1.13155087	1.14477132	0.955393576
1.131562451	1.144896227	0.955392973
1.13157403	1.145021133	0.955392359
1.134439633	1.14514604	0.955391735
1.138994458	1.145270946	0.955391102
1.134461851	1.145395853	0.95539074
1.131132834	1.145520759	0.955390817
1.131631896	1.145645666	0.955390438
1.136336788	1.145770572	0.95538972
1.14106389	1.145895479	0.95538904
1.141074673	1.146020385	0.955388812
1.137049477	1.146145292	0.955389037
1.13706025	1.146270198	0.955389251
1.133613262	1.146395105	0.955389056
1.133624499	1.146520011	0.955388849
1.134105793	1.146644918	0.95538829
1.134116954	1.146769824	0.95538772
1.139579244	1.146894731	0.955387184
1.139589887	1.147019637	0.955386636
1.136683031	1.147144544	0.955386612
1.136693843	1.14726945	0.955386577
1.142110046	1.147394357	0.955386244
1.14212111	1.147519263	0.955385901
1.142132177	1.14764417	0.955386079
1.142143249	1.147769076	0.955386247
1.146373508	1.147893983	0.955386404
1.146388741	1.148018889	0.955386549
1.147787495	1.148143796	0.955387097
1.142895257	1.148268702	0.955387634
1.142432525	1.148393609	0.955388294
1.142443718	1.148518515	0.955388461
1.142454916	1.148643422	0.955388568
1.148264295	1.148768328	0.955388663
1.150023985	1.148893235	0.955388745
1.150046782	1.149018141	0.955389384
1.150069631	1.149143048	0.955390183
1.146873589	1.149267954	0.955390971
1.145928159	1.149392861	0.955391747

1.145942677	1.149517767	0.955392197
1.148004441	1.149642674	0.955392542
1.145971781	1.14976758	0.955392878
1.145986366	1.149892487	0.955393405
1.146000975	1.150017393	0.955393722
1.146015605	1.1501423	0.955394029
1.15720673	1.150267206	0.955394327
1.157243049	1.150392113	0.955394616
1.172432411	1.150517019	0.955395997
1.17245386	1.150641926	0.95539737
1.175826044	1.150766832	0.955400226
1.177623334	1.150891739	0.955403071
1.177636858	1.151016645	0.955406235
1.177650376	1.151141552	0.955409562
1.178226143	1.151266458	0.955412878
1.177677393	1.151391365	0.955416181
1.174932706	1.151516271	0.955419527
1.176543356	1.151641178	0.955422804
1.173485294	1.151766084	0.955425797
1.17350441	1.151890991	0.955428934
1.173523486	1.152015897	0.955431755
1.15286701	1.152140804	0.955434564
1.156279428	1.15226571	0.955437362
1.146283351	1.152390617	0.955438113
1.143783791	1.152515523	0.955439186
1.146971262	1.15264043	0.955439262
1.165087363	1.152765336	0.955439077
1.165123419	1.152890243	0.955439194
1.16515943	1.153015149	0.955441082
1.165195398	1.153140056	0.955442961
1.16523132	1.153264962	0.95544483
1.176742018	1.153389869	0.955446689
1.168123579	1.153514775	0.955448538
1.177351708	1.153639682	0.955451508
1.177365373	1.153764588	0.955453615
1.174548852	1.153889495	0.955456619
1.174565879	1.154014401	0.95545961
1.172602848	1.154139308	0.95546231
1.175286372	1.154264214	0.955464999
1.176255224	1.154389121	0.955467479
1.174633685	1.154514027	0.955470211
1.172686797	1.154638934	0.955473024
1.172707665	1.15476384	0.955475664
1.17025794	1.154888747	0.955478099

1.167161691	1.155013653	0.955480523
1.16346465	1.15513856	0.955482692
1.163502297	1.155263466	0.955484543
1.163539915	1.155388373	0.955486017
1.157207486	1.155513279	0.955487482
1.154363704	1.155638186	0.955488936
1.154395777	1.155763092	0.955489754
1.151384379	1.155887999	0.955490279
1.14942973	1.156012905	0.955490793
1.149451172	1.156137812	0.955490997
1.147824309	1.156262718	0.955490994
1.14784226	1.156387625	0.955490979
1.144434737	1.156512531	0.955490789
1.14444735	1.156637438	0.955490588
1.144459976	1.156762344	0.955490037
1.144472614	1.156887251	0.955489475
1.145029045	1.157012157	0.9554889
1.154752406	1.157137064	0.955488315
1.179975664	1.15726197	0.955487773
1.179989014	1.157386877	0.955488176
1.180002366	1.157511783	0.955491052
1.180015721	1.15763669	0.955493918
1.18002908	1.157761596	0.955496773
1.17892852	1.157886503	0.955499618
1.183594657	1.158011409	0.955502454
1.179232669	1.158136316	0.955505169
1.168644917	1.158261222	0.955508332
1.146699802	1.158386129	0.955511052
1.141934704	1.158511035	0.955512718
1.141715493	1.158635942	0.95551221
1.141726428	1.158760848	0.95551122
1.141737366	1.158885755	0.955510197
1.143160251	1.159010661	0.955509162
1.141075796	1.159135568	0.955508117
1.140410978	1.159260474	0.9555072
1.14042167	1.159385381	0.955506066
1.140432362	1.159510287	0.955504853
1.140443056	1.159635194	0.95550363
1.140453751	1.1597601	0.955502396
1.139346347	1.159885007	0.955501152
1.1425342	1.160009913	0.955499897
1.142545438	1.16013482	0.955498521
1.142556682	1.160259726	0.955497446
1.14662141	1.160384633	0.955496358

1.149302529	1.160509539	0.955495259
1.14665275	1.160634446	0.955494548
1.14332298	1.160759352	0.955494088
1.1466842	1.160884259	0.955493355
1.146699966	1.161009165	0.955492281
1.146715761	1.161134072	0.95549154
1.147069942	1.161258978	0.955490805
1.146420477	1.161383885	0.955490074
1.144148758	1.161508791	0.955489383
1.144161093	1.161633698	0.955488632
1.144173439	1.161758604	0.95548766
1.146481877	1.161883511	0.955486693
1.153986026	1.162008417	0.955485731
1.163990745	1.162133324	0.955484999
1.180512727	1.16225823	0.95548501
1.185436261	1.162383137	0.95548601
1.189797669	1.162508043	0.955488639
1.189816156	1.16263295	0.955491754
1.189834651	1.162757856	0.955495301
1.188333891	1.162882763	0.95549885
1.188351595	1.163007669	0.955502403
1.182655595	1.163132576	0.95550581
1.182670043	1.163257482	0.955509221
1.180919204	1.163382389	0.955512071
1.180932846	1.163507295	0.955514925
1.180946493	1.163632202	0.955517607
1.179273001	1.163757108	0.955520293
1.179286239	1.163882015	0.95552298
1.177624391	1.164006921	0.955525503
1.176168893	1.164131828	0.955528031
1.17618355	1.164256734	0.955530397
1.176501572	1.164381641	0.955532622
1.179909727	1.164506547	0.955534851
1.179923062	1.164631454	0.955537113
1.180783209	1.16475636	0.955539713
1.1807968	1.164881267	0.955542316
1.179405441	1.165006173	0.955545006
1.185452463	1.16513108	0.9555477
1.185468507	1.165255986	0.95555026
1.185484561	1.165380893	0.955553417
1.179180491	1.165505799	0.955556579
1.177229429	1.165630706	0.955559745
1.168555229	1.165755612	0.955562293
1.177256898	1.165880519	0.955564654

1.17727062	1.166005425	0.955566163
1.177284333	1.166130332	0.955568533
1.182411437	1.166255238	0.955570909
1.181248083	1.166380145	0.95557329
1.181261856	1.166505051	0.955576181
1.184002371	1.166629958	0.955578961
1.187773063	1.166754864	0.955581747
1.187790454	1.166879771	0.955584806
1.187807855	1.167004677	0.955588238
1.184063206	1.167129584	0.955591675
1.180770118	1.16725449	0.955595115
1.174950521	1.167379397	0.95559819
1.166998682	1.167504303	0.955600941
1.167031914	1.16762921	0.955603123
1.169633191	1.167754116	0.955604525
1.169661141	1.167879023	0.955605933
1.167131266	1.168003929	0.955607601
1.157832781	1.168128836	0.955609275
1.156352903	1.168253742	0.955610704
1.156388157	1.168378649	0.955611221
1.150758334	1.168503555	0.955611597
1.156458809	1.168628462	0.95561198
1.166630621	1.168753368	0.955611813
1.15652965	1.168878275	0.955612212
1.150350879	1.169003181	0.955613616
1.150374425	1.169128088	0.95561403
1.146748343	1.169252994	0.955613839
1.148612685	1.169377901	0.955613656
1.148632307	1.169502807	0.95561312
1.148651972	1.169627714	0.955612774
1.160713918	1.16975262	0.955612435
1.16075244	1.169877527	0.955612103
1.173056907	1.170002433	0.955612967
1.185421154	1.17012734	0.95561384
1.188636566	1.170252246	0.95561593
1.185453215	1.170377153	0.955619241
1.185469259	1.170502059	0.955622874
1.185485313	1.170626966	0.955626199
1.18386671	1.170751872	0.95562953
1.183881828	1.170876779	0.955632867
1.183269671	1.171001685	0.955636051
1.176373326	1.171126592	0.955639241
1.158767866	1.171251498	0.955642376
1.151889382	1.171376405	0.955644838

1.151916412	1.171501311	0.955645575
1.151943501	1.171626218	0.955645642
1.15197065	1.171751124	0.955645721
1.151997859	1.171876031	0.955645809
1.154529199	1.172000937	0.955645907
1.154561571	1.172125844	0.955646014
1.156004668	1.17225075	0.955646375
1.157540016	1.172375657	0.955646746
1.159946754	1.172500563	0.955647265
1.159985107	1.17262547	0.955647939
1.157650174	1.172750376	0.955648854
1.159259473	1.172875283	0.955649776
1.159297522	1.173000189	0.95565047
1.159335592	1.173125096	0.955651326
1.161792484	1.173250002	0.955652191
1.16183094	1.173374909	0.955653065
1.161869387	1.173499815	0.955654185
1.181180092	1.173624722	0.955655314
1.188018254	1.173749628	0.955656452
1.188035783	1.173874535	0.955659495
1.188053322	1.173999441	0.955663217
1.191964085	1.174124348	0.955666943
1.181538842	1.174249254	0.955670673
1.18155274	1.174374161	0.955674792
1.177051302	1.174499067	0.955677888
1.17706516	1.174623974	0.955680991
1.181594474	1.17474888	0.955683655
1.177092846	1.174873787	0.955686327
1.177961546	1.174998693	0.955689452
1.185727431	1.1751236	0.95569214
1.185743637	1.175248506	0.95569492
1.181084179	1.175373413	0.95569847
1.17657434	1.175498319	0.955702026
1.168405801	1.175623226	0.955705127
1.173935755	1.175748132	0.955707788
1.177203215	1.175873039	0.955709649
1.17721697	1.175997945	0.955712058
1.185857348	1.176122852	0.955714792
1.180893356	1.176247758	0.955717532
1.17949913	1.176372665	0.955721125
1.17698201	1.176497571	0.955724232
1.176995917	1.176622478	0.955727207
1.180662516	1.176747384	0.955729938
1.187360403	1.176872291	0.955732675

1.181553247	1.176997197	0.955735779
1.181567152	1.177122104	0.955739548
1.180151129	1.17724701	0.955742751
1.180164522	1.177371917	0.955745962
1.180177918	1.177496823	0.955749038
1.177961685	1.17762173	0.955752121
1.177975075	1.177746636	0.955755212
1.181944505	1.177871543	0.955758091
1.181664664	1.177996449	0.955760978
1.180244953	1.178121356	0.955764262
1.177175821	1.178246262	0.955767524
1.177189594	1.178371169	0.955770652
1.177203358	1.178496075	0.955773484
1.17397276	1.178620982	0.955776322
1.167877978	1.178745888	0.955779168
1.160294153	1.178870795	0.955781704
1.167941166	1.178995701	0.955783647
1.160371066	1.179120608	0.955784849
1.160409535	1.179245514	0.955786811
1.160448011	1.179370421	0.955788034
1.167396693	1.179495327	0.95578927
1.17291728	1.179620234	0.955790519
1.175524154	1.17974514	0.955792461
1.175210286	1.179870047	0.955794953
1.175226211	1.179994953	0.95579771
1.175242111	1.18011986	0.955800442
1.173431414	1.180244766	0.955803183
1.178255333	1.180369673	0.955805934
1.187861933	1.180494579	0.955808515
1.178838533	1.180619486	0.955811578
1.179685462	1.180744392	0.955815594
1.175001147	1.180869299	0.955818728
1.175017402	1.180994205	0.955821951
1.179725341	1.181119112	0.955824721
1.183552386	1.181119112	0.955827498
1.186201902	1.181119112	0.955830747
1.186218387	1.181119112	0.955834379
1.183597228	1.181119112	0.95583828
1.179513014	1.181119112	0.955842189
1.180084845	1.181119112	0.955845847
1.176417576	1.181119112	0.955849111
1.176431971	1.181119112	0.955852438
1.176446352	1.181119112	0.955855412
1.175524315	1.181119112	0.955858392

1.17553978	1.181119112	0.955861379
1.172543473	1.181119112	0.955864279
1.168222799	1.181119112	0.955867186
1.168253747	1.181119112	0.955869804
1.17260691	1.181119112	0.955872
1.17262796	1.181119112	0.955874204
1.175305637	1.181119112	0.95587684
1.175647433	1.181119112	0.955879484
1.175662729	1.181119112	0.955882394
1.175678004	1.181119112	0.955885343
1.172732493	1.181119112	0.955888298
1.176632056	1.181119112	0.955891261
1.177231504	1.181119112	0.955893938
1.177245242	1.181119112	0.955897006
1.176067839	1.181119112	0.955900138
1.174045387	1.181119112	0.955903278
1.169268491	1.181119112	0.95590631
1.169297247	1.181119112	0.955909148
1.16932594	1.181119112	0.955911521
1.17411707	1.181119112	0.955913904
1.180138507	1.181119112	0.955916296
1.180151896	1.181119112	0.955919166
1.180165289	1.181119112	0.955922637
1.180178686	1.181119112	0.955926114
1.180192085	1.181119112	0.9559296
1.180205489	1.181119112	0.955933092
1.180218896	1.181119112	0.955936593
1.174968898	1.181119112	0.955940101
1.172670133	1.181119112	0.955943618
1.171771269	1.181119112	0.955946624
1.171794254	1.181119112	0.95594941
1.171817186	1.181119112	0.955952114
1.171840063	1.181119112	0.955954826
1.171862887	1.181119112	0.955957547
1.178375619	1.181119112	0.955960276
1.178667222	1.181119112	0.955963013
1.178680464	1.181119112	0.955966397
1.189178798	1.181119112	0.955969817
1.189196961	1.181119112	0.955973244
1.189215133	1.181119112	0.95597771
1.179566704	1.181119112	0.955982183
1.175525041	1.181119112	0.955986665
1.173772317	1.181119112	0.955990202
1.173790837	1.181119112	0.955993348

1.173809319	1.181119112	0.955996327
1.175586765	1.181119112	0.955999315
1.175919876	1.181119112	0.956002311
1.17495316	1.181119112	0.956005488
1.17594975	1.181119112	0.956008703
1.178296354	1.181119112	0.956011829
1.178309653	1.181119112	0.95601506
1.177761669	1.181119112	0.956018529
1.177775133	1.181119112	0.956022005
1.17505076	1.181119112	0.956025433
1.175066935	1.181119112	0.956028869
1.173623468	1.181119112	0.956032042
1.173642294	1.181119112	0.956035222
1.177559502	1.181119112	0.956038266
1.17367983	1.181119112	0.95604132
1.171977031	1.181119112	0.956044765
1.17371721	1.181119112	0.956047835
1.174117906	1.181119112	0.956050743
1.179301893	1.181119112	0.956053828
1.179315134	1.181119112	0.956056959
1.175555705	1.181119112	0.956060607
1.175571125	1.181119112	0.956064262
1.174920585	1.181119112	0.956067553
1.174936971	1.181119112	0.95607085
1.175290739	1.181119112	0.956074087
1.175306542	1.181119112	0.956077332
1.175322321	1.181119112	0.956080616
1.175338077	1.181119112	0.956083908
1.175353809	1.181119112	0.956087207
1.183227375	1.181119112	0.956090516
1.188710997	1.181119112	0.956093833
1.188728908	1.181119112	0.956097933
1.188746829	1.181119112	0.95610258
1.182977893	1.181119112	0.956107235
1.1887827	1.181119112	0.956111898
1.19319259	1.181119112	0.956116
1.189197339	1.181119112	0.956120679
1.183036422	1.181119112	0.956125799
1.177328274	1.181119112	0.95613053
1.172502938	1.181119112	0.956134659
1.171586769	1.181119112	0.956138232
1.172545462	1.181119112	0.956141335
1.177382941	1.181119112	0.956144354
1.173433419	1.181119112	0.956147476

1.172608886	1.181119112	0.956151082
1.171703315	1.181119112	0.956154307
1.172650928	1.181119112	0.956157461
1.173100378	1.181119112	0.956160535
1.173529141	1.181119112	0.956163711
1.174672229	1.181119112	0.95616694
1.175370091	1.181119112	0.956170221
1.175385776	1.181119112	0.956173624
1.175401438	1.181119112	0.956177104
1.175417077	1.181119112	0.956180595
1.175432694	1.181119112	0.956184096
1.175448289	1.181119112	0.956187608
1.175463861	1.181119112	0.956191129
1.175800504	1.181119112	0.956194661
1.175815599	1.181119112	0.956198201
1.175830675	1.181119112	0.956201782
1.175845731	1.181119112	0.956205372
1.175212112	1.181119112	0.956208972
1.174536861	1.181119112	0.956212583
1.174189397	1.181119112	0.956216139
1.174207091	1.181119112	0.956219637
1.174587916	1.181119112	0.956223109
1.175617979	1.181119112	0.95622659
1.175950605	1.181119112	0.956230102
1.175648629	1.181119112	0.956233708
1.174295058	1.181119112	0.956237339
1.174312552	1.181119112	0.956240934
1.174689213	1.181119112	0.956244387
1.175385939	1.181119112	0.956247835
1.178641631	1.181119112	0.956251313
1.18327198	1.181119112	0.956254853
1.182371796	1.181119112	0.956258705
1.179793032	1.181119112	0.956263004
1.178416358	1.181119112	0.956267207
1.178429634	1.181119112	0.956271147
1.178442907	1.181119112	0.956274944
1.178456177	1.181119112	0.956278734
1.180990738	1.181119112	0.956282517
1.179038477	1.181119112	0.956286294
1.178495977	1.181119112	0.956290313
1.178509239	1.181119112	0.956294132
1.1785225	1.181119112	0.95629789
1.178257343	1.181119112	0.956301641
1.178549361	1.181119112	0.956305385

1.180234053	1.181119112	0.956309095
1.182272419	1.181119112	0.956312825
1.182286669	1.181119112	0.956316713
1.180274309	1.181119112	0.956320795
1.17944872	1.181119112	0.956324869
1.178906335	1.181119112	0.956328735
1.176647505	1.181119112	0.95633251
1.176361659	1.181119112	0.956336223
1.17637611	1.181119112	0.956339704
1.176390547	1.181119112	0.956343151
1.176404969	1.181119112	0.956346592
1.176419376	1.181119112	0.956350027
1.17643377	1.181119112	0.956353454
1.176746498	1.181119112	0.956356874
1.176760592	1.181119112	0.956360287
1.177641294	1.181119112	0.956363721
1.17765481	1.181119112	0.95636715
1.17766832	1.181119112	0.956370656
1.177681823	1.181119112	0.956374155
1.17653413	1.181119112	0.956377646
1.175618699	1.181119112	0.956381131
1.174970768	1.181119112	0.956384493
1.174278354	1.181119112	0.956387758
1.174295879	1.181119112	0.95639095
1.174313371	1.181119112	0.956394067
1.17433083	1.181119112	0.956397178
1.174348257	1.181119112	0.956400283
1.177233222	1.181119112	0.956403383
1.176957066	1.181119112	0.956406478
1.176970992	1.181119112	0.956409848
1.176984908	1.181119112	0.956413184
1.182102646	1.181119112	0.956416513
1.182717578	1.181119112	0.956419834
1.185958591	1.181119112	0.95642365
1.185974934	1.181119112	0.956427515
1.185650077	1.181119112	0.956431689
1.185666238	1.181119112	0.956435855
1.182790057	1.181119112	0.956439977
1.182804576	1.181119112	0.956444092
1.182819103	1.181119112	0.956447913
1.182833638	1.181119112	0.956451724
1.180220921	1.181119112	0.956455527
1.180801049	1.181119112	0.95645932
1.180814646	1.181119112	0.956462846

1.180828249	1.181119112	0.956466417
1.180841857	1.181119112	0.95646998
1.178058104	1.181119112	0.956473534
1.178071463	1.181119112	0.95647708
1.178084818	1.181119112	0.956480343
1.180896338	1.181119112	0.956483597
1.181779408	1.181119112	0.956486845
1.186602916	1.181119112	0.956490362
1.189564336	1.181119112	0.956493958
1.184281477	1.181119112	0.95649802
1.178721838	1.181119112	0.956502366
1.176143895	1.181119112	0.956506184
1.176158579	1.181119112	0.956509448
1.176173246	1.181119112	0.956512451
1.181598026	1.181119112	0.956515449
1.181611952	1.181119112	0.95651844
1.181625883	1.181119112	0.956521954
1.181639821	1.181119112	0.956525459
1.176246342	1.181119112	0.956528955
1.176260914	1.181119112	0.956532444
1.17498826	1.181119112	0.956535393
1.17746613	1.181119112	0.956538334
1.177479732	1.181119112	0.956541141
1.179171532	1.181119112	0.956544184
1.175053205	1.181119112	0.956547221
1.173606963	1.181119112	0.956550415
1.172355036	1.181119112	0.956553196
1.171424013	1.181119112	0.956555824
1.170399546	1.181119112	0.956558318
1.170961858	1.181119112	0.95656071
1.172880386	1.181119112	0.956562989
1.172002344	1.181119112	0.956565313
1.172921206	1.181119112	0.956567814
1.172941547	1.181119112	0.956570219
1.174155758	1.181119112	0.956572705
1.172982095	1.181119112	0.956575184
1.173002301	1.181119112	0.956577773
1.173022463	1.181119112	0.956580238
1.17304258	1.181119112	0.956582695
1.173062653	1.181119112	0.956585146
1.171232448	1.181119112	0.95658759
1.175967066	1.181119112	0.956590027
1.177466272	1.181119112	0.956592274
1.177193108	1.181119112	0.956594979

1.174690962	1.181119112	0.956597821
1.179462529	1.181119112	0.956600627
1.179475787	1.181119112	0.956603174
1.179489046	1.181119112	0.95660618
1.179502307	1.181119112	0.956609177
1.17951557	1.181119112	0.956612163
1.179528834	1.181119112	0.95661514
1.178151815	1.181119112	0.956618107
1.177316298	1.181119112	0.956621064
1.176449122	1.181119112	0.956623874
1.176463486	1.181119112	0.95662659
1.175213522	1.181285654	0.95662921
1.175558196	1.181452196	0.956631821
1.175573613	1.181618738	0.956634299
1.176520803	1.18178528	0.956636801
1.175604384	1.181951822	0.956639294
1.174244854	1.182118364	0.956641869
1.174262443	1.182284906	0.956644342
1.174279998	1.182451448	0.95664667
1.17429752	1.18261799	0.956648989
1.174315009	1.182784532	0.9566513
1.174332465	1.182951074	0.956653602
1.174349889	1.183117616	0.956655894
1.175726637	1.183284158	0.956658177
1.175741827	1.1834507	0.95666045
1.177261616	1.183617242	0.956662849
1.177275334	1.183783784	0.956665238
1.177289045	1.183950326	0.956667767
1.177302747	1.184116868	0.956670287
1.178444427	1.184283411	0.956672798
1.179569123	1.184449953	0.956675297
1.182762024	1.184616495	0.956677898
1.186703903	1.184783037	0.956680596
1.186720682	1.184949579	0.956683597
1.18673747	1.185116121	0.956686975
1.186754267	1.185282663	0.956690343
1.186771075	1.185449205	0.956693701
1.186787892	1.185615747	0.956697049
1.186804719	1.185782289	0.956700388
1.186821556	1.185948831	0.956703718
1.184144697	1.186115373	0.956707039
1.184159975	1.186281915	0.956710351
1.182019275	1.186448457	0.95671339
1.182033399	1.186614999	0.95671642

1.183259437	1.186781541	0.956719229
1.183274212	1.186948083	0.956722029
1.185554571	1.187114625	0.956724939
1.185570675	1.187281167	0.956727841
1.183947044	1.187447709	0.956730958
1.181822404	1.187614252	0.956734067
1.181543329	1.187780794	0.956737007
1.179291411	1.187947336	0.95673973
1.179861622	1.188113878	0.956742417
1.182174648	1.18828042	0.956744876
1.182188848	1.188446962	0.956747382
1.182203056	1.188613504	0.956750108
1.181047157	1.188780046	0.956752828
1.179093044	1.188946588	0.956755542
1.179106274	1.18911313	0.956758133
1.179119503	1.189279672	0.956760523
1.179132734	1.189446214	0.956762904
1.181115667	1.189612756	0.956765277
1.178881531	1.189779298	0.956767642
1.17889476	1.18994584	0.956770192
1.178907989	1.190112382	0.956772514
1.181170932	1.190278924	0.956774827
1.185539063	1.190445466	0.956777132
1.187296955	1.190612008	0.956779648
1.187314075	1.19077855	0.956782584
1.19074022	1.190945093	0.95678568
1.190759178	1.191111635	0.956788766
1.190778145	1.191278177	0.956792179
1.193679309	1.191444719	0.956795581
1.194988015	1.191611261	0.956798974
1.195444146	1.191777803	0.956802642
1.195903942	1.191944345	0.956806427
1.201353994	1.192110887	0.956810245
1.19594591	1.192277429	0.956814096
1.195966904	1.192443971	0.956818473
1.195987903	1.192610513	0.956822307
1.196008909	1.192777055	0.956826133
1.193861689	1.192943597	0.956829951
1.19019536	1.193110139	0.95683376
1.190214048	1.193276681	0.956837346
1.188323475	1.193443223	0.956840561
1.185172223	1.193609765	0.956843766
1.185188102	1.193776307	0.956846774
1.184872454	1.193942849	0.95684946

1.188394329	1.194109391	0.956852136
1.188412066	1.194275934	0.95685477
1.184919589	1.194442476	0.95685774
1.184283236	1.194609018	0.956860698
1.184298594	1.19477556	0.956863299
1.184313962	1.194942102	0.956865824
1.184982566	1.195108644	0.956868335
1.190439038	1.195275186	0.956870833
1.190457848	1.195441728	0.956873382
1.190476667	1.19560827	0.956876455
1.190495496	1.195774812	0.956879514
1.190514334	1.195941354	0.95688256
1.206022612	1.196107896	0.956885593
1.225445762	1.196274438	0.956888614
1.226023411	1.19644098	0.956893148
1.226036674	1.196607522	0.956899582
1.226049928	1.196774064	0.956906059
1.220802688	1.196940606	0.956912523
1.221158903	1.197107148	0.956918976
1.229648505	1.19727369	0.9569249
1.22218681	1.197440232	0.956930845
1.221207221	1.197606775	0.956937613
1.220884163	1.197773317	0.956943633
1.217668455	1.197939859	0.956949543
1.212473539	1.198106401	0.956955407
1.20766796	1.198272943	0.956960942
1.211661202	1.198439485	0.956965953
1.210816808	1.198606027	0.956970477
1.210837542	1.198772569	0.956975381
1.21085827	1.198939111	0.956980189
1.200435895	1.199105653	0.956984986
1.210899707	1.199272195	0.956989771
1.210920416	1.199438737	0.95699354
1.216315809	1.199605279	0.956998348
1.216334416	1.199771821	0.957003167
1.216353014	1.199938363	0.957008527
1.207452238	1.200104905	0.957013897
1.196072947	1.200271447	0.957019278
1.196093977	1.200437989	0.957023791
1.196115013	1.200604531	0.957027194
1.207538441	1.200771073	0.95703061
1.207559982	1.200937616	0.957034038
1.207581518	1.201104158	0.957038603
1.197985487	1.2012707	0.957043182

1.193197392	1.201437242	0.957047775
1.193217438	1.201603784	0.957051435
1.198956452	1.201770326	0.957054635
1.211661416	1.201936868	0.957057851
1.205892895	1.20210341	0.957061645
1.200851765	1.202269952	0.957066703
1.195905144	1.202436494	0.957071206
1.193337883	1.202603036	0.957075226
1.192108602	1.202769578	0.957078773
1.195092758	1.20293612	0.957082079
1.200961319	1.203102662	0.957085277
1.200983235	1.203269204	0.957088783
1.194720965	1.203435746	0.95709288
1.192206639	1.203602288	0.957096991
1.191815988	1.20376883	0.957100497
1.191026638	1.203935372	0.957103766
1.189861834	1.204101914	0.957107009
1.189880353	1.204268457	0.957110184
1.189511971	1.204434999	0.957113256
1.189147888	1.204601541	0.957116339
1.188413083	1.204768083	0.957119397
1.189184189	1.204934625	0.95712243
1.189202355	1.205101167	0.957125402
1.18922053	1.205267709	0.957128461
1.191198821	1.205434251	0.957131533
1.193700913	1.205600793	0.957134619
1.195884387	1.205767335	0.957137911
1.195905362	1.205933877	0.957141462
1.198610599	1.206100419	0.95714524
1.201838284	1.206266961	0.957149033
1.201860253	1.206433503	0.957153105
1.201882222	1.206600045	0.957157507
1.201904192	1.206766587	0.957161921
1.201926162	1.206933129	0.957166347
1.193458821	1.207099671	0.957170786
1.1951967	1.207266213	0.957175238
1.186174792	1.207432755	0.957178866
1.186191261	1.207599298	0.957182676
1.18620774	1.20776584	0.957185608
1.186224228	1.207932382	0.957188553
1.189917613	1.208098924	0.957191511
1.194454658	1.208265466	0.957194482
1.194475175	1.208432008	0.957197828
1.197579688	1.20859855	0.957201633

1.197601099	1.208765092	0.957205452
1.196728343	1.208931634	0.957209587
1.19674955	1.209098176	0.957213736
1.196770763	1.209264718	0.95721781
1.197686788	1.20943126	0.957221898
1.197708222	1.209597802	0.957225999
1.19908757	1.209764344	0.957230202
1.199109266	1.209930886	0.957234417
1.210942305	1.210097428	0.957238779
1.220738453	1.21026397	0.957243154
1.225460649	1.210430512	0.957248703
1.225474251	1.210597054	0.957255226
1.230076364	1.210763596	0.957262222
1.231817107	1.210930139	0.957269229
1.231826926	1.211096681	0.957276697
1.235374365	1.211263223	0.957284346
1.235382468	1.211429765	0.957292005
1.235390568	1.211596307	0.957300022
1.231866147	1.211762849	0.95730805
1.226143379	1.211929391	0.957316089
1.211190234	1.212095933	0.957323791
1.218450611	1.212262475	0.957330941
1.218468177	1.212429017	0.957336629
1.218485735	1.212595559	0.957343044
1.218503283	1.212762101	0.95734947
1.218520823	1.212928643	0.957355909
1.210880934	1.213095185	0.957362361
1.210901649	1.213261727	0.957368824
1.21511016	1.213428269	0.957374545
1.218222075	1.213594811	0.957380277
1.218239758	1.213761353	0.957386433
1.222420316	1.213927895	0.957392904
1.217902444	1.214094437	0.957399386
1.217920288	1.21426098	0.957406291
1.217938122	1.214427522	0.95741276
1.217955947	1.214594064	0.957419241
1.217973764	1.214760606	0.957425734
1.217991572	1.214927148	0.95743224
1.221876078	1.21509369	0.957438757
1.225569378	1.215260232	0.957445287
1.225582914	1.215426774	0.957452211
1.22472886	1.215593316	0.957459508
1.224742901	1.215759858	0.957466816
1.221954541	1.2159264	0.957474051

1.213375353	1.216092942	0.957481296
1.210403211	1.216259484	0.957488276
1.216632685	1.216426026	0.957494422
1.216651144	1.216592568	0.957500285
1.211765065	1.21675911	0.957506772
1.209163678	1.216925652	0.957513271
1.209184867	1.217092194	0.957519297
1.20920605	1.217258736	0.957525078
1.211846753	1.217425278	0.957530871
1.209248401	1.217591821	0.957536677
1.211887556	1.217758363	0.957542752
1.209290731	1.217924905	0.957548581
1.209311888	1.218091447	0.95755468
1.211088011	1.218257989	0.957560533
1.214065466	1.218424531	0.957566398
1.216093999	1.218591073	0.957572448
1.216112708	1.218757615	0.957578801
1.216131408	1.218924157	0.957585364
1.21414375	1.219090699	0.957591939
1.217705954	1.219257241	0.957598525
1.217723895	1.219423783	0.957604926
1.222938522	1.219590325	0.957611688
1.227285953	1.219756867	0.95761846
1.233500641	1.219923409	0.957625756
1.233509566	1.220089951	0.95763349
1.227323423	1.220256493	0.957641845
1.225132664	1.220423035	0.95765021
1.230704052	1.220589577	0.957657975
1.230714505	1.220756119	0.957665533
1.230724952	1.220922662	0.957673647
1.228893653	1.221089204	0.957681769
1.226051584	1.221255746	0.9576899
1.223104517	1.221422288	0.957697861
1.223119519	1.22158883	0.957705551
1.220145623	1.221755372	0.957712959
1.220509741	1.221921914	0.957720377
1.223164473	1.222088456	0.957727513
1.220542681	1.222254998	0.957734695
1.217291716	1.22242154	0.957742149
1.21730986	1.222588082	0.957749355
1.218080846	1.222754624	0.957756251
1.217724082	1.222921166	0.957763157
1.217742014	1.223087708	0.957770148
1.217759937	1.22325425	0.957777113

1.218151809	1.223420792	0.957784088
1.218905232	1.223587334	0.957791073
1.218922565	1.223753876	0.957798105
1.21965852	1.223920418	0.95780522
1.225718112	1.22408696	0.957812344
1.225731559	1.224253503	0.95781955
1.23028121	1.224420045	0.957827363
1.23029191	1.224586587	0.957835187
1.22816613	1.224753129	0.95784347
1.223104283	1.224919671	0.957851765
1.23119869	1.225086213	0.95785986
1.231208858	1.225252755	0.95786747
1.225256704	1.225419297	0.957875887
1.220870091	1.225585839	0.957884314
1.220886367	1.225752381	0.957892164
1.225297853	1.225918923	0.95789959
1.225311553	1.226085465	0.957907024
1.223843415	1.226252007	0.9579149
1.223857983	1.226418549	0.957922786
1.226742526	1.226585091	0.957930535
1.226755353	1.226751633	0.957938296
1.225945956	1.226918175	0.95794635
1.225959265	1.227084717	0.957954415
1.225972566	1.227251259	0.95796241
1.22598586	1.227417801	0.957970417
1.225999145	1.227584344	0.957978436
1.221080971	1.227750886	0.957986466
1.225174683	1.227917428	0.957994512
1.225188457	1.22808397	0.958002087
1.220095898	1.228250512	0.95801008
1.220112598	1.228417054	0.958018087
1.223119832	1.228583596	0.958025604
1.227173854	1.228750138	0.958033135
1.227186419	1.22891668	0.958040974
1.227198977	1.229083222	0.958049224
1.227211527	1.229249764	0.958057486
1.230152819	1.229416306	0.958065761
1.227236605	1.229582848	0.958074048
1.230174364	1.22974939	0.958082635
1.231280243	1.229915932	0.958090945
1.231501492	1.230082474	0.958099556
1.23130048	1.230249016	0.958108286
1.231521478	1.230415558	0.958117049
1.231531463	1.2305821	0.958125803

1.231541442	1.230748642	0.95813459
1.231340886	1.230915185	0.95814339
1.231561383	1.231081727	0.958152202
1.231571346	1.231248269	0.958161005
1.236985045	1.231414811	0.958169842
1.236992722	1.231581353	0.958178691
1.243758618	1.231747895	0.958188084
1.243771501	1.231914437	0.958197488
1.246298285	1.232080979	0.958207569
1.250255045	1.232247521	0.958217664
1.246333049	1.232414063	0.958228019
1.246350479	1.232580605	0.958238774
1.241493728	1.232747147	0.958249153
1.241503427	1.232913689	0.958259546
1.238344348	1.233080231	0.958269471
1.235253693	1.233246773	0.958279409
1.235090172	1.233413315	0.958289046
1.229506378	1.233579857	0.95829839
1.229282205	1.233746399	0.958307729
1.229293498	1.233912941	0.958316528
1.225678495	1.234079483	0.958325316
1.224828066	1.234246026	0.958334114
1.225705428	1.234412568	0.958342569
1.226276422	1.23457911	0.95835095
1.229816505	1.234745652	0.958359429
1.231581406	1.234912194	0.958367975
1.229606859	1.235078736	0.958376881
1.229617958	1.235245278	0.958385971
1.229629051	1.23541182	0.958394877
1.229640137	1.235578362	0.958403796
1.234507622	1.235744904	0.958412726
1.237832376	1.235911446	0.958421667
1.237840003	1.236077988	0.958431098
1.234532968	1.23624453	0.958440867
1.231250054	1.236411072	0.958450648
1.235073827	1.236577614	0.958460114
1.23933398	1.236744156	0.958469269
1.241532691	1.236910698	0.958478812
1.241542434	1.23707724	0.958488783
1.240043287	1.23707724	0.958498981
1.240051665	1.23707724	0.95850919
1.240060048	1.23707724	0.958519262
1.240068436	1.23707724	0.958529345
1.240076831	1.23707724	0.958539439

1.240085231	1.23707724	0.958549544
1.238421253	1.23707724	0.958559659
1.23617201	1.23707724	0.958569782
1.236179858	1.23707724	0.958579749
1.232416718	1.23707724	0.958589504
1.228179216	1.23707724	0.958599268
1.227162001	1.23707724	0.958608673
1.227174574	1.23707724	0.95861767
1.227187139	1.23707724	0.958626578
1.228226999	1.23707724	0.958635497
1.228975385	1.23707724	0.958644428
1.228986861	1.23707724	0.958653472
1.228998331	1.23707724	0.9586626
1.229009794	1.23707724	0.95867174
1.23211052	1.23707724	0.958680894
1.236924027	1.23707724	0.958690061
1.239526663	1.23707724	0.958699543
1.238061567	1.23707724	0.958709511
1.23546464	1.23707724	0.958719746
1.232359773	1.23707724	0.958729848
1.235480779	1.23707724	0.958739705
1.236320562	1.23707724	0.958749268
1.238260223	1.23707724	0.95875915
1.238753114	1.23707724	0.958769128
1.236992962	1.23707724	0.958779308
1.236351787	1.23707724	0.958789549
1.234843113	1.23707724	0.958799628
1.231621642	1.23707724	0.958809656
1.235033081	1.23707724	0.958819548
1.237031331	1.23707724	0.958829135
1.235561299	1.23707724	0.958839072
1.233439393	1.23707724	0.958849219
1.231036646	1.23707724	0.958859233
1.228263194	1.23707724	0.95886905
1.2282751	1.23707724	0.958878643
1.228779765	1.23707724	0.958887975
1.230642515	1.23707724	0.958897323
1.232727189	1.23707724	0.958906734
1.232736512	1.23707724	0.958916341
1.23274583	1.23707724	0.958926168
1.231955749	1.23707724	0.958936008
1.235311128	1.23707724	0.958945861
1.241199284	1.23707724	0.958955648
1.245191491	1.23707724	0.958965777

1.245206835	1.23707724	0.9589765
1.245222205	1.23707724	0.958987629
1.245237604	1.23707724	0.95899877
1.243773046	1.23707724	0.959009926
1.242307754	1.23707724	0.959021094
1.237825186	1.23707724	0.959032128
1.237832813	1.23707724	0.959043028
1.23784044	1.23707724	0.959053498
1.237848068	1.23707724	0.959063979
1.237855696	1.23707724	0.959074472
1.237863324	1.23707724	0.959084976
1.238031167	1.23707724	0.959095492
1.241952346	1.23707724	0.959106019
1.242635413	1.23707724	0.959116573
1.242646577	1.23707724	0.959127524
1.242657756	1.23707724	0.959138555
1.24481695	1.23707724	0.959149601
1.242680163	1.23707724	0.959160661
1.242459026	1.23707724	0.959171948
1.242024349	1.23707724	0.959183039
1.24120876	1.23707724	0.959194121
1.236824436	1.23707724	0.959205174
1.236180308	1.23707724	0.959216159
1.232615364	1.23707724	0.959226726
1.230314906	1.23707724	0.959237244
1.229169792	1.23707724	0.959247422
1.229181152	1.23707724	0.959257387
1.2316417	1.23707724	0.959267252
1.232662227	1.23707724	0.959277131
1.236235183	1.23707724	0.959287264
1.236243014	1.23707724	0.959297509
1.238023808	1.23707724	0.959308118
1.238514069	1.23707724	0.95931874
1.242394236	1.23707724	0.959329549
1.242405064	1.23707724	0.959340419
1.242415906	1.23707724	0.959351686
1.240592415	1.23707724	0.959362968
1.239374647	1.23707724	0.959374264
1.23743693	1.23707724	0.959385396
1.235481241	1.23707724	0.959396423
1.237452194	1.23707724	0.959407273
1.23940665	1.23707724	0.959417946
1.23941466	1.23707724	0.959428827
1.238761325	1.23707724	0.959439915

1.239944062	1.23707724	0.959451019
1.239952373	1.23707724	0.959462072
1.23996069	1.23707724	0.959473256
1.240689631	1.23707724	0.959484455
1.240698515	1.23707724	0.959495671
1.240707408	1.23707724	0.959506973
1.239994012	1.23707724	0.959518291
1.238823531	1.23707724	0.959529625
1.236901482	1.23707724	0.959540881
1.236909172	1.23707724	0.959552011
1.233475468	1.23707724	0.959562943
1.230197508	1.23707724	0.959573866
1.228311455	1.23707724	0.959584441
1.227561598	1.23707724	0.959594684
1.227573928	1.23707724	0.959604732
1.227586251	1.23707724	0.959614697
1.228358918	1.23707724	0.959624654
1.230261911	1.23707724	0.959634604
1.231362315	1.23707724	0.95964462
1.23137239	1.23707724	0.959654816
1.231799809	1.23707724	0.959665111
1.238606756	1.23707724	0.959675398
1.244048852	1.23707724	0.959685718
1.244062213	1.23707724	0.9596967
1.244075597	1.23707724	0.959708209
1.244089002	1.23707724	0.959719709
1.244102431	1.23707724	0.959731199
1.242816203	1.23707724	0.959742679
1.242827627	1.23707724	0.959754148
1.242839068	1.23707724	0.959765479
1.240375286	1.23707724	0.9597768
1.238522139	1.23707724	0.959788111
1.236601175	1.23707724	0.959799168
1.235449332	1.23707724	0.959810033
1.236940199	1.23707724	0.959820697
1.237909736	1.23707724	0.959831237
1.238560703	1.23707724	0.959841912
1.238568421	1.23707724	0.959852672
1.238576141	1.23707724	0.959863487
1.238583862	1.23707724	0.959874293
1.237947894	1.23707724	0.959885088
1.236993961	1.23707724	0.959895874
1.237001637	1.23707724	0.959906586
1.237009312	1.23707724	0.959917195

1.236531479	1.23707724	0.959927795
1.234150904	1.23707724	0.959938385
1.234159516	1.23707724	0.959948917
1.234168125	1.23707724	0.959959206
1.23417673	1.23707724	0.959969483
1.234185331	1.23707724	0.959979751
1.239665268	1.23707724	0.959990007
1.249397196	1.23707724	0.960000252
1.252108114	1.23707724	0.960011025
1.25213628	1.23707724	0.960022745
1.252164497	1.23707724	0.96003472
1.252192763	1.23707724	0.960046686
1.249513621	1.23707724	0.960058645
1.247305919	1.23707724	0.960070596
1.247325173	1.23707724	0.960082274
1.247344463	1.23707724	0.960093725
1.246583401	1.23707724	0.960105167
1.246601298	1.23707724	0.960116603
1.246619228	1.23707724	0.960127954
1.247837978	1.23707724	0.960139297
1.247858236	1.23707724	0.960150634
1.247878532	1.23707724	0.960162082
1.247898867	1.23707724	0.960173523
1.244375945	1.23707724	0.960184957
1.244389859	1.23707724	0.960196384
1.244403797	1.23707724	0.960207457
1.244129635	1.23707724	0.960218522
1.244143132	1.23707724	0.960229581
1.244156651	1.23707724	0.960240602
1.242394969	1.23707724	0.960251615
1.246458777	1.23707724	0.960262618
1.251022741	1.23707724	0.960273438
1.253424767	1.23707724	0.960284648
1.253455233	1.23707724	0.960296297
1.251101511	1.23707724	0.960308173
1.248166196	1.23707724	0.960320042
1.248187074	1.23707724	0.960331671
1.244572928	1.23707724	0.960343
1.241831525	1.23707724	0.960354322
1.242056215	1.23707724	0.96036528
1.242066594	1.23707724	0.960375958
1.242076987	1.23707724	0.96038665
1.242087393	1.23707724	0.960397333
1.237024818	1.23707724	0.960408009

1.232848825	1.23707724	0.960418678
1.232858084	1.23707724	0.960428839
1.239145222	1.23707724	0.960438581
1.243851953	1.23707724	0.960448316
1.246782307	1.23707724	0.960458663
1.243878045	1.23707724	0.960469467
1.237879456	1.23707724	0.960480551
1.237887085	1.23707724	0.960491342
1.237894715	1.23707724	0.960501534
1.244502179	1.23707724	0.960511718
1.24903259	1.23707724	0.960521894
1.249055103	1.23707724	0.960532711
1.245162399	1.23707724	0.960543963
1.241811473	1.23707724	0.960555208
1.239240191	1.23707724	0.960566059
1.24458733	1.23707724	0.960576571
1.245552875	1.23707724	0.96058682
1.241851838	1.23707724	0.960597586
1.239271953	1.23707724	0.960608437
1.239279902	1.23707724	0.960618914
1.239287854	1.23707724	0.960629128
1.236055469	1.23707724	0.960639332
1.236063349	1.23707724	0.960649527
1.236071227	1.23707724	0.960659393
1.229464414	1.23707724	0.960669249
1.229475598	1.23707724	0.960679094
1.229486776	1.23707724	0.96068828
1.229960655	1.23707724	0.960697455
1.232522441	1.23707724	0.960706622
1.232531873	1.23707724	0.960715827
1.2325413	1.23707724	0.960725275
1.232550723	1.23707724	0.960734716
1.23256014	1.23707724	0.960744151
1.232569552	1.23707724	0.960753579
1.232578959	1.23707724	0.960763
1.232588361	1.23707724	0.960772415
1.232597758	1.23707724	0.960781823
1.233188447	1.23707724	0.960791226
1.23319753	1.23707724	0.960800621
1.233206608	1.23707724	0.960810067
1.233215681	1.23707724	0.960819504
1.237666557	1.23707724	0.960828931
1.240514645	1.23707724	0.96083835
1.240896978	1.23707724	0.960848197

1.240906053	1.23707724	0.960858315
1.240915137	1.23707724	0.960868458
1.24131401	1.23707724	0.960878591
1.241731796	1.23707724	0.960888714
1.24133302	1.23707724	0.960898867
1.241751761	1.23707724	0.960909051
1.241352071	1.23707724	0.960919185
1.241361613	1.23707724	0.96092935
1.24041936	1.23707724	0.960939465
1.239552356	1.23707724	0.960949572
1.239560439	1.23707724	0.960959576
1.240815776	1.23707724	0.960969486
1.240824775	1.23707724	0.960979389
1.241219665	1.23707724	0.960989408
1.24522485	1.23707724	0.960999421
1.241043006	1.23707724	0.961009466
1.245928852	1.23707724	0.961019899
1.250236864	1.23707724	0.961029915
1.250261628	1.23707724	0.961040406
1.250286437	1.23707724	0.961051314
1.250311292	1.23707724	0.961062218
1.24674644	1.23707724	0.961073117
1.241505752	1.23707724	0.96108401
1.241515465	1.23707724	0.961094544
1.241525188	1.23707724	0.961104553
1.240205313	1.23707724	0.961114553
1.235778919	1.23707724	0.961124546
1.232923529	1.23707724	0.961134398
1.230665397	1.23707724	0.961143806
1.232151221	1.23707724	0.961152922
1.2340742	1.23707724	0.961161806
1.235818715	1.23707724	0.961170826
1.236485776	1.23707724	0.961180025
1.234459082	1.23707724	0.961189385
1.234108769	1.23707724	0.961198801
1.232006175	1.23707724	0.961208009
1.234305999	1.23707724	0.961217174
1.236686965	1.23707724	0.961226124
1.237499207	1.23707724	0.961235293
1.235546977	1.23707724	0.961244689
1.235555019	1.23707724	0.961254158
1.23343216	1.23707724	0.961263427
1.231028358	1.23707724	0.961272691
1.229928289	1.23707724	0.96128174

1.229939198	1.23707724	0.961290546
1.230177448	1.23707724	0.961299239
1.230188209	1.23707724	0.961307928
1.230198964	1.23707724	0.961316635
1.230209713	1.23707724	0.961325338
1.230220456	1.23707724	0.961334035
1.232747625	1.23707724	0.961342728
1.235133454	1.23707724	0.961351415
1.239225074	1.23707724	0.961360344
1.246567118	1.23707724	0.9613695
1.24777974	1.23707724	0.961379052
1.247799887	1.23707724	0.961389318
1.247820073	1.23707724	0.961399696
1.247840297	1.23707724	0.961410067
1.244938205	1.23707724	0.961420432
1.243788441	1.23707724	0.96143079
1.243026202	1.23707724	0.961440854
1.242109605	1.23707724	0.961450795
1.240708937	1.23707724	0.961460653
1.239821523	1.23707724	0.961470413
1.239657935	1.23707724	0.961480027
1.239666074	1.23707724	0.961489545
1.239674218	1.23707724	0.961499037
1.239682366	1.23707724	0.961508521
1.240213982	1.23707724	0.961517997
1.239027828	1.23707724	0.961527465
1.235117143	1.23707724	0.961536975
1.231110693	1.23707724	0.96154636
1.236142766	1.23707724	0.96155535
1.2408073	1.23707724	0.961563936
1.244560329	1.23707724	0.96157301
1.240825291	1.23707724	0.961582534
1.240104372	1.23707724	0.961592417
1.238926276	1.23707724	0.961601923
1.235010552	1.23707724	0.961611347
1.234845104	1.23707724	0.961620644
1.238949738	1.23707724	0.961629545
1.240146528	1.23707724	0.961638421
1.239978859	1.23707724	0.96164769
1.239987193	1.23707724	0.961657067
1.238981056	1.23707724	0.961666417
1.236079923	1.23707724	0.961675758
1.237064244	1.23707724	0.96168499
1.240559209	1.23707724	0.961693928

1.24056798	1.23707724	0.961702954
1.240576759	1.23707724	0.961712314
1.237094904	1.23707724	0.961721665
1.237102567	1.23707724	0.961731004
1.237270848	1.23707724	0.96173999
1.237278491	1.23707724	0.961748966
1.243971905	1.23707724	0.961757949
1.243985138	1.23707724	0.961766922
1.240096265	1.23707724	0.961776544
1.234289616	1.23707724	0.961786155
1.240113098	1.23707724	0.961795373
1.25126437	1.23707724	0.961804008
1.251291019	1.23707724	0.961813206
1.242773037	1.23707724	0.961823491
1.24087956	1.23707724	0.961833767
1.240515554	1.23707724	0.961843194
1.238485018	1.23707724	0.961852422
1.23849272	1.23707724	0.961861603
1.238500424	1.23707724	0.96187057
1.238508129	1.23707724	0.961879526
1.236911054	1.23707724	0.96188847
1.235771491	1.23707724	0.961897403
1.235779458	1.23707724	0.961906169
1.235787422	1.23707724	0.96191481
1.235117527	1.23707724	0.96192344
1.233134897	1.23707724	0.961932059
1.233144007	1.23707724	0.961940602
1.233153113	1.23707724	0.961948939
1.236158639	1.23707724	0.961957266
1.23778173	1.23707724	0.961965584
1.238592994	1.23707724	0.961974186
1.239593444	1.23707724	0.961982939
1.238608446	1.23707724	0.961991764
1.237812236	1.23707724	0.962000678
1.237819863	1.23707724	0.962009487
1.23782749	1.23707724	0.962018208
1.237835117	1.23707724	0.962026919
1.23671859	1.23707724	0.962035622
1.234712172	1.23707724	0.962044317
1.234720536	1.23707724	0.962052892
1.233080232	1.23707724	0.962061261
1.233089371	1.23707724	0.96206962
1.232514183	1.237327053	0.962077806
1.231919526	1.237576867	0.962085983

1.23422984	1.23782668	0.962094091
1.237423493	1.238076493	0.96210213
1.236625896	1.238326306	0.962110385
1.236307461	1.238576119	0.962118943
1.236315273	1.238825932	0.962127411
1.233352261	1.239075745	0.962135835
1.236330892	1.239325558	0.962144247
1.236338699	1.239575371	0.962152355
1.240656292	1.239825184	0.962160743
1.242289435	1.240074998	0.96216912
1.238778725	1.240324811	0.962177911
1.238786498	1.240574624	0.96218685
1.242321534	1.240824437	0.962195433
1.245635746	1.24107425	0.962204005
1.245651892	1.241324063	0.962212914
1.248857811	1.241573876	0.962222139
1.248879995	1.241823689	0.962231354
1.24260491	1.242073502	0.962240873
1.238354544	1.242323315	0.962250382
1.236432229	1.242573129	0.96225926
1.240394288	1.242822942	0.962267708
1.238539126	1.243072755	0.962275954
1.238546839	1.243322568	0.962284579
1.238554554	1.243572381	0.962293008
1.238400833	1.243822194	0.962301425
1.23840852	1.244072007	0.962309831
1.238416207	1.24432182	0.96231821
1.245523708	1.244571633	0.962326576
1.26396623	1.244821446	0.962334931
1.264010949	1.24507126	0.962343974
1.249683024	1.245321073	0.962354821
1.249706757	1.245570886	0.962365659
1.245604128	1.245820699	0.962375074
1.245620217	1.246070512	0.96238448
1.245636335	1.246320325	0.962393472
1.245652482	1.246570138	0.962402454
1.248403031	1.246819951	0.962411426
1.248424357	1.247069764	0.962420388
1.248445723	1.247319577	0.962429609
1.248467129	1.247569391	0.96243882
1.248488576	1.247819204	0.962448022
1.245089901	1.248069017	0.962457213
1.252171706	1.24831883	0.962466394
1.252804452	1.248568643	0.96247523

1.252833845	1.248818456	0.962484751
1.241382271	1.249068269	0.962494323
1.236641737	1.249318082	0.962503888
1.236649473	1.249567895	0.962512315
1.236657208	1.249817708	0.962520264
1.238593436	1.250067522	0.962528206
1.244604585	1.250317335	0.962536139
1.251835821	1.250567148	0.962544253
1.243777009	1.250816961	0.962552948
1.238624348	1.251066774	0.962562343
1.243802856	1.251316587	0.962570931
1.248360169	1.2515664	0.962578999
1.266203341	1.251816213	0.962587563
1.248402698	1.252066026	0.962596561
1.248424023	1.252315839	0.962607302
1.248445389	1.252565653	0.962616274
1.244448967	1.252815466	0.962625232
1.244463006	1.253065279	0.962634178
1.252143036	1.253315092	0.962642716
1.264597687	1.253564905	0.962651242
1.252199543	1.253814718	0.962660511
1.261917861	1.254064531	0.962670994
1.254821877	1.254314344	0.962680243
1.254854679	1.254564157	0.962690438
1.249105205	1.25481397	0.962699922
1.256349518	1.255063784	0.962709398
1.256384739	1.255313597	0.962718295
1.249173282	1.25556341	0.962727895
1.249196059	1.255813223	0.962737485
1.24105381	1.256063036	0.962746354
1.241063039	1.256312849	0.962755213
1.241072277	1.256562662	0.96276326
1.237515498	1.256812475	0.962771296
1.237523126	1.257062288	0.962779321
1.237530754	1.257312101	0.962786984
1.23608062	1.257561915	0.962794635
1.23657995	1.257811728	0.962802274
1.236911655	1.258061541	0.962809757
1.236919343	1.258311354	0.962817278
1.244187364	1.258561167	0.96282482
1.27055665	1.25881098	0.962832349
1.265603046	1.259060793	0.962840581
1.265649177	1.259310606	0.962851395
1.242452155	1.259560419	0.962861706

1.265741546	1.259810232	0.962872008
1.268762821	1.260060046	0.962880011
1.268810983	1.260309859	0.962890292
1.268859168	1.260559672	0.962900856
1.268907378	1.260809485	0.962911407
1.265973096	1.261059298	0.962921946
1.266019512	1.261309111	0.962932505
1.2605408	1.261558924	0.962942791
1.262346644	1.261808737	0.962953097
1.262389734	1.26205855	0.962962878
1.2581761	1.262308363	0.962972853
1.258214006	1.262558177	0.962982848
1.258251964	1.26280799	0.962992445
1.256001902	1.263057803	0.963002061
1.254627704	1.263307616	0.963011699
1.254660189	1.263557429	0.963021131
1.254692727	1.263807242	0.963030446
1.254725318	1.264057055	0.96303978
1.252200869	1.264306868	0.963049136
1.254790661	1.264556681	0.963058511
1.242452268	1.264806494	0.963067656
1.242463175	1.265056308	0.963077074
1.295966861	1.265306121	0.963085294
1.3095651	1.265555934	0.963093533
1.309575774	1.265805747	0.963107056
1.309586447	1.26605556	0.963121933
1.287641701	1.266305373	0.963136827
1.287681903	1.266555186	0.963151736
1.300035145	1.266804999	0.963164501
1.260582243	1.267054812	0.963177286
1.271195226	1.267304625	0.963191302
1.271244306	1.267554439	0.96320145
1.2712934	1.267804252	0.963212661
1.271342505	1.268054065	0.963223893
1.271391623	1.268303878	0.963235148
1.271440753	1.268553691	0.963246426
1.286331195	1.268803504	0.963257726
1.271539047	1.269053317	0.963269049
1.271588211	1.26930313	0.963281857
1.271637386	1.269552943	0.963293227
1.257727142	1.269802756	0.96330462
1.25776441	1.27005257	0.963316037
1.248172716	1.270302383	0.963326104
1.246952608	1.270552196	0.963336194

1.246971197	1.270802009	0.96334536
1.250669045	1.271051822	0.963354425
1.250694606	1.271301635	0.963363512
1.250720214	1.271551448	0.963372982
1.250745869	1.271801261	0.963382474
1.265100673	1.272051074	0.963391988
1.265146396	1.272300887	0.963401524
1.265192156	1.272550701	0.963412493
1.291009395	1.272800514	0.963423486
1.298792253	1.273050327	0.963434502
1.298814417	1.27330014	0.96344808
1.291115472	1.273549953	0.963462442
1.279665202	1.273799766	0.963476825
1.279712946	1.274049579	0.963490468
1.277736273	1.274299392	0.963503001
1.277784944	1.274549205	0.963515556
1.277833596	1.274799018	0.963527933
1.277882229	1.275048832	0.963540333
1.276907977	1.275298645	0.963552755
1.250593569	1.275548458	0.963565199
1.277005876	1.275798271	0.963577565
1.25064446	1.276048084	0.963587358
1.277103714	1.276297897	0.96359977
1.292248742	1.27654771	0.963609604
1.294280751	1.276797523	0.963622063
1.262263064	1.277047336	0.963636031
1.262306064	1.277297149	0.963650216
1.262349111	1.277546963	0.963661266
1.288632391	1.277796776	0.963672337
1.288671272	1.278046589	0.96368343
1.288710098	1.278296402	0.963697127
1.297341481	1.278546215	0.963710843
1.288787591	1.278796028	0.96372458
1.283558956	1.279045841	0.963739184
1.288044259	1.279295654	0.963752963
1.298903489	1.279545467	0.963766243
1.304916653	1.27979528	0.963779981
1.30492978	1.280045094	0.963794805
1.304942893	1.280294907	0.963810237
1.304955992	1.28054472	0.963825686
1.295941227	1.280794533	0.963841151
1.263883658	1.281044346	0.963856633
1.270854841	1.281294159	0.963871242
1.296022654	1.281543972	0.963882707

1.270952828	1.281793785	0.963894874
1.267952865	1.282043598	0.963909533
1.263175675	1.282293411	0.963921737
1.258868728	1.282543225	0.963933661
1.258907588	1.282793038	0.963945129
1.263307663	1.283042851	0.963956188
1.26066678	1.283292664	0.963967267
1.264330768	1.283542477	0.963978795
1.264375822	1.28379229	0.963990078
1.264420916	1.284042103	0.964001738
1.264466051	1.284291916	0.964013416
1.260873119	1.284541729	0.964025114
1.259220357	1.284791542	0.964036833
1.259259688	1.285041356	0.964048216
1.260997518	1.285291169	0.964059454
1.261039083	1.285540982	0.964070714
1.261080697	1.285790795	0.964082164
1.26112236	1.286040608	0.964093635
1.267761475	1.286290421	0.964105128
1.267809094	1.286540234	0.964116644
1.269879463	1.286790047	0.964128829
1.282153114	1.28703986	0.964141036
1.282199132	1.287289673	0.964153464
1.295528904	1.287539487	0.964167115
1.295556817	1.2877893	0.964180785
1.282336961	1.288039113	0.964195781
1.297268232	1.288288926	0.964210794
1.282428658	1.288538739	0.964224519
1.287037806	1.288788552	0.964239729
1.295695651	1.289038365	0.96425349
1.304298824	1.289288178	0.964267721
1.304312618	1.289537991	0.964282818
1.304326397	1.289787804	0.964298776
1.30434016	1.290037618	0.964314749
1.298926488	1.290287431	0.964330737
1.298948418	1.290537244	0.96434674
1.292049676	1.290787057	0.964362224
1.292083454	1.29103687	0.964377726
1.301485117	1.291286683	0.964392564
1.292150848	1.291536496	0.96440742
1.291467503	1.291786309	0.964423215
1.28306617	1.292036122	0.964438104
1.274056535	1.292285935	0.964452939
1.274105956	1.292535749	0.964466962

1.274155377	1.292785562	0.964480113
1.274204796	1.293035375	0.964493286
1.277350827	1.293285188	0.96450648
1.276371852	1.293535001	0.964519694
1.276420958	1.293784814	0.964533233
1.276470051	1.294034627	0.964546691
1.276519132	1.29428444	0.964560171
1.282566391	1.294534253	0.964573671
1.282612065	1.294784066	0.964587193
1.282657701	1.29503388	0.964601327
1.288905636	1.295283693	0.96461548
1.288944138	1.295533506	0.964629653
1.288982587	1.295783319	0.964644456
1.301449892	1.296033132	0.964659277
1.30146761	1.296282945	0.964674116
1.301485301	1.296532758	0.964690198
1.307264684	1.296782571	0.964706297
1.307276014	1.297032384	0.964722414
1.307287339	1.297282197	0.964739114
1.302269571	1.297532011	0.96475583
1.288480005	1.297781824	0.964772563
1.302302488	1.298031637	0.964788818
1.296685927	1.29828145	0.964803732
1.28859711	1.298531263	0.964820025
1.286084015	1.298781076	0.96483578
1.288674914	1.299030889	0.964850756
1.288713735	1.299280702	0.964865502
1.288752504	1.299530515	0.96488052
1.296840503	1.299780328	0.964895559
1.308110779	1.300030142	0.96491062
1.308121753	1.300279955	0.964926497
1.307196994	1.300529768	0.9649435
1.311518147	1.300779581	0.964960521
1.311528894	1.301029394	0.964977469
1.312908234	1.301279207	0.96499486
1.311550394	1.30152902	0.96501227
1.311561146	1.301778833	0.965029834
1.3115719	1.302028646	0.965047281
1.311582656	1.302278459	0.965064746
1.300774647	1.302528273	0.965082228
1.306074255	1.302778086	0.965099726
1.287647777	1.303027899	0.965116175
1.28768797	1.303277712	0.965133162
1.299166083	1.303527525	0.965148351

1.299187597	1.303777338	0.96516356
1.299209074	1.304027151	0.965179916
1.299230514	1.304276964	0.96519629
1.299251917	1.304526777	0.965212685
1.299273283	1.30477659	0.965229099
1.299294612	1.305026404	0.965245531
1.306194743	1.305276217	0.965261984
1.31286459	1.30552603	0.965278457
1.311282566	1.305775843	0.965295627
1.311293283	1.306025656	0.965313471
1.309961062	1.306275469	0.965331176
1.309971714	1.306525282	0.965348899
1.306515584	1.306775095	0.965366508
1.309993015	1.307024908	0.965384136
1.306539121	1.307274721	0.965401441
1.306550877	1.307524535	0.965419106
1.306562626	1.307774348	0.96543645
1.306574367	1.308024161	0.965453812
1.3065861	1.308273974	0.965471192
1.313228849	1.308523787	0.965488589
1.310738074	1.3087736	0.965506004
1.317173803	1.309023413	0.965524089
1.317186159	1.309273226	0.965541944
1.317198519	1.309523039	0.965560448
1.318803458	1.309772852	0.965578968
1.318816402	1.310022666	0.965597504
1.318829351	1.310272479	0.965616214
1.323467887	1.310522292	0.96563494
1.321681293	1.310772105	0.965653682
1.321695284	1.311021918	0.965672898
1.32170928	1.311271731	0.965691956
1.321430457	1.311521544	0.965711033
1.321737288	1.311771357	0.965730131
1.316550104	1.31202117	0.965749219
1.31656224	1.312270983	0.965768356
1.318674412	1.312520797	0.965787
1.318687309	1.31277061	0.965805662
1.316092601	1.313020423	0.965824549
1.318713117	1.313270236	0.965843456
1.318726028	1.313520049	0.965862124
1.318738943	1.313769862	0.965881067
1.318751864	1.314019675	0.965900028
1.316659164	1.314269488	0.965919007
1.321597233	1.314519301	0.965938001

1.316683517	1.314769114	0.965956804
1.317471239	1.315018928	0.965976107
1.311690522	1.315268741	0.965994941
1.317496167	1.315518554	0.966013868
1.309021555	1.315768367	0.96603224
1.309032299	1.31601818	0.966051198
1.305965718	1.316267993	0.966069337
1.314296051	1.316517806	0.966087492
1.314307446	1.316767619	0.966105362
1.312220352	1.316767619	0.966124069
1.328423372	1.316767619	0.966142794
1.312930903	1.316767619	0.966161332
1.328455936	1.316767619	0.966181483
1.31508999	1.316767619	0.966200125
1.321514082	1.316767619	0.966220314
1.315113281	1.316767619	0.966239203
1.315866045	1.316767619	0.966258742
1.313462908	1.316767619	0.966277668
1.315889851	1.316767619	0.966296686
1.314194056	1.316767619	0.966315485
1.314205421	1.316767619	0.96633454
1.31421679	1.316767619	0.966353446
1.313518731	1.316767619	0.96637237
1.312832214	1.316767619	0.966391313
1.311251037	1.316767619	0.966410205
1.312394488	1.316767619	0.966429047
1.311272464	1.316767619	0.96644775
1.310610743	1.316767619	0.966466584
1.309503371	1.316767619	0.966485326
1.309064912	1.316767619	0.966504024
1.308850171	1.316767619	0.966522632
1.308860947	1.316767619	0.966541216
1.308871721	1.316767619	0.966559798
1.308882493	1.316767619	0.966578401
1.308893262	1.316767619	0.966597023
1.308677559	1.316767619	0.966615666
1.308688372	1.316767619	0.966634329
1.309823282	1.316767619	0.966652989
1.309833939	1.316767619	0.966671669
1.311637185	1.316767619	0.966690477
1.311874299	1.316767619	0.966709304
1.311433337	1.316767619	0.966728327
1.309876561	1.316767619	0.966747391
1.310110767	1.316767619	0.96676643

1.311917524	1.316767619	0.966785333
1.311928335	1.316767619	0.966804276
1.310142711	1.316767619	0.966823414
1.310153359	1.316767619	0.96684257
1.311960779	1.316767619	0.966861569
1.318341928	1.316767619	0.966880587
1.318087507	1.316767619	0.966899802
1.327060533	1.316767619	0.966919665
1.327076378	1.316767619	0.966939522
1.327092228	1.316767619	0.966960282
1.328457124	1.316767619	0.966981063
1.328473414	1.316767619	0.967001865
1.328489709	1.316767619	0.967022822
1.325198356	1.316767619	0.9670438
1.323337457	1.316767619	0.967064798
1.316635842	1.316767619	0.96708549
1.316648008	1.316767619	0.967106016
1.312558382	1.316767619	0.9671259
1.31120842	1.316767619	0.967145801
1.31010023	1.316767619	0.967165318
1.310110878	1.316767619	0.967184722
1.310121526	1.316767619	0.967204036
1.309236888	1.316767619	0.96722337
1.309247599	1.316767619	0.967242725
1.309258308	1.316767619	0.967262012
1.309269016	1.316767619	0.96728132
1.309279722	1.316767619	0.967300649
1.309065415	1.316767619	0.96732
1.309301131	1.316767619	0.967339371
1.30975983	1.316767619	0.96735874
1.309097619	1.316767619	0.967378152
1.308882717	1.316767619	0.967397628
1.30911908	1.316767619	0.967417059
1.309578609	1.316767619	0.967436455
1.310259949	1.316767619	0.967455861
1.310270597	1.316767619	0.967475299
1.315382767	1.316767619	0.96749479
1.321570634	1.316767619	0.967514268
1.321584586	1.316767619	0.967534236
1.323091031	1.316767619	0.967554799
1.323105526	1.316767619	0.967575349
1.323120026	1.316767619	0.967596034
1.323134531	1.316767619	0.967616705
1.323149041	1.316767619	0.967637364

1.319668588	1.316767619	0.967658009
1.312406015	1.316767619	0.967678643
1.31241692	1.316767619	0.967698922
1.312427827	1.316767619	0.967718474
1.312438737	1.316767619	0.967738015
1.312449649	1.316767619	0.967757543
1.315300824	1.316767619	0.967777059
1.327092971	1.316767619	0.967796563
1.328457463	1.316767619	0.967816335
1.328473754	1.316767619	0.967837255
1.328490049	1.316767619	0.967858296
1.328506349	1.316767619	0.967879326
1.328522655	1.316767619	0.967900343
1.320404116	1.316767619	0.967921349
1.316393789	1.316767619	0.967942343
1.314194529	1.316767619	0.967962526
1.312340747	1.316767619	0.967982302
1.312351639	1.316767619	0.968001851
1.312362532	1.316767619	0.968021206
1.312373428	1.316767619	0.968040553
1.312384326	1.316767619	0.968059891
1.314262773	1.316767619	0.96807922
1.316237546	1.316767619	0.968098541
1.316249573	1.316767619	0.968118036
1.317547704	1.316767619	0.968137715
1.317560193	1.316767619	0.968157383
1.317572687	1.316767619	0.968177167
1.31338575	1.316767619	0.968196941
1.309994347	1.316767619	0.968216705
1.309781649	1.316767619	0.968236044
1.309792308	1.316767619	0.968255039
1.309802966	1.316767619	0.968274004
1.309813624	1.316767619	0.968292961
1.309824281	1.316767619	0.968311909
1.309611126	1.316767619	0.968330846
1.309845594	1.316767619	0.968349775
1.310974129	1.316767619	0.968368673
1.310984813	1.316767619	0.968387586
1.310547634	1.316767619	0.9684066
1.312362929	1.316767619	0.968425607
1.314240426	1.316767619	0.96844456
1.314251805	1.316767619	0.968463683
1.312395624	1.316767619	0.968482982
1.311498502	1.316767619	0.968502271

1.312417432	1.316767619	0.968521366
1.311745922	1.316767619	0.968540363
1.310856774	1.316767619	0.968559442
1.310867448	1.316767619	0.968578444
1.313385866	1.316767619	0.968597351
1.313397004	1.316767619	0.968616249
1.313408145	1.316767619	0.968635385
1.31341929	1.316767619	0.968654512
1.313430437	1.316767619	0.96867363
1.312976276	1.316767619	0.968692739
1.314161281	1.316767619	0.96871184
1.315137923	1.316767619	0.968730885
1.323984612	1.316767619	0.968750035
1.331744312	1.316767619	0.968769272
1.331400155	1.316767619	0.968789371
1.324029053	1.316767619	0.968810223
1.31721288	1.316767619	0.968831033
1.313766137	1.316767619	0.968851107
1.317237624	1.316767619	0.968870502
1.319669348	1.316767619	0.96888955
1.318050802	1.316767619	0.96890893
1.318063473	1.316767619	0.96892854
1.315760912	1.316767619	0.968947981
1.315772778	1.316767619	0.968967416
1.315289786	1.316767619	0.968986615
1.315301495	1.316767619	0.969005808
1.315313207	1.316767619	0.969024945
1.317874024	1.316767619	0.969044074
1.315336643	1.316767619	0.969063197
1.313901249	1.316767619	0.969082563
1.315360095	1.316767619	0.96910167
1.317398567	1.316767619	0.969120628
1.320689879	1.316767619	0.969139721
1.317949404	1.316767619	0.969159004
1.321004529	1.316767619	0.969178603
1.321018276	1.316767619	0.969197923
1.316685801	1.316767619	0.969217535
1.314709813	1.316767619	0.969237139
1.318547908	1.316767619	0.969256307
1.321073311	1.316767619	0.969275272
1.318573614	1.316767619	0.969294607
1.31379999	1.316767619	0.969314184
1.311962019	1.316767619	0.969333509
1.312200378	1.316767619	0.969352357

1.311983658	1.316767619	0.969371018
1.312450388	1.316767619	0.969389695
1.31457194	1.316767619	0.969408342
1.315313329	1.316767619	0.969427029
1.312483137	1.316767619	0.969445916
1.312037791	1.316767619	0.969464869
1.312504982	1.316767619	0.969483535
1.315360217	1.316767619	0.969502149
1.31512651	1.316767619	0.969520802
1.322745963	1.316767619	0.969539728
1.328556386	1.316767619	0.969558621
1.322774714	1.316767619	0.969578257
1.314929365	1.316767619	0.969598455
1.311220634	1.316767619	0.969618074
1.311908348	1.316767619	0.969636914
1.312603686	1.316767619	0.96965538
1.311929968	1.316767619	0.969673905
1.311940781	1.316767619	0.969692488
1.312636535	1.316767619	0.969710993
1.31264749	1.316767619	0.969729492
1.311973232	1.316767619	0.96974805
1.312669407	1.316767619	0.969766601
1.312680369	1.316767619	0.969785079
1.312691334	1.316767619	0.969803618
1.312702301	1.316767619	0.969822149
1.311800468	1.316767619	0.969840672
1.3138905	1.316767619	0.969859187
1.313901777	1.316767619	0.969877605
1.311832847	1.316767619	0.969896221
1.311391861	1.316767619	0.96991483
1.310953487	1.316767619	0.969933228
1.310293114	1.316767619	0.969951574
1.310303762	1.316767619	0.969969866
1.310985537	1.316767619	0.969988084
1.311897371	1.316767619	0.970006295
1.313054206	1.316767619	0.970024564
1.313065259	1.316767619	0.970042914
1.313076315	1.316767619	0.970061369
1.313087374	1.316767619	0.970079816
1.313098436	1.316767619	0.970098256
1.3131095	1.316767619	0.970116688
1.314298424	1.316767619	0.970135114
1.320002581	1.316767619	0.970153534
1.318638231	1.316767619	0.970172064

1.323866946	1.316767619	0.970191147
1.318127292	1.316767619	0.970210088
1.31340919	1.316767619	0.970229535
1.313420334	1.316767619	0.970248408
1.318165401	1.316767619	0.970266807
1.318178113	1.316767619	0.9702852
1.31819083	1.316767619	0.970304051
1.317411716	1.316767619	0.970322892
1.317424157	1.316767619	0.970341725
1.319314179	1.316767619	0.970360472
1.316419656	1.316767619	0.970379212
1.319340445	1.316767619	0.970398128
1.327604143	1.316767619	0.97041675
1.327620163	1.316767619	0.970435651
1.327636188	1.316767619	0.970455357
1.333317718	1.316767619	0.970475054
1.333335417	1.316767619	0.970494742
1.333353121	1.316767619	0.970514981
1.32976097	1.316767619	0.97053521
1.32977766	1.316767619	0.97055543
1.329794355	1.316767619	0.970575285
1.324807049	1.316767619	0.970595134
1.316565022	1.316767619	0.970614977
1.312484047	1.316767619	0.970634321
1.312494969	1.316767619	0.970652847
1.312505892	1.316767619	0.970670964
1.311157016	1.316767619	0.970689075
1.309825502	1.316767619	0.970707178
1.309836159	1.316767619	0.970725141
1.313242895	1.316767619	0.970742968
1.318497753	1.316767619	0.970760789
1.315174127	1.316767619	0.970778938
1.317201481	1.316767619	0.970797595
1.317213847	1.316767619	0.970815916
1.317226217	1.316767619	0.970834427
1.317238591	1.316767619	0.97085293
1.31725097	1.316767619	0.970871426
1.317263354	1.316767619	0.970889915
1.315255902	1.316767619	0.970908398
1.315513706	1.316767619	0.970926873
1.315279301	1.316767619	0.97094514
1.314561539	1.316767619	0.970963424
1.31223371	1.316767619	0.970981675
1.314584496	1.316767619	0.970999847

1.31459598	1.316767619	0.971017781
1.312266326	1.316767619	0.971035938
1.312277202	1.316767619	0.971054087
1.31228808	1.316767619	0.971071997
1.312298961	1.316767619	0.971089898
1.312081867	1.316767619	0.971107792
1.311865579	1.316767619	0.971125678
1.31210355	1.316767619	0.971143535
1.312114394	1.316767619	0.971161364
1.311897991	1.316767619	0.971179209
1.311908798	1.316767619	0.971197047
1.311919607	1.316767619	0.971214855
1.311930419	1.316767619	0.971232655
1.311714933	1.316767619	0.971250448
1.311500125	1.316767619	0.971268235
1.311736489	1.316767619	0.971285992
1.319433142	1.316767619	0.971303722
1.319446315	1.316767619	0.971321468
1.315291128	1.316767619	0.971339963
1.315302837	1.316767619	0.971358453
1.31531455	1.316767619	0.971376525
1.315326266	1.316767619	0.971394592
1.320352374	1.316767619	0.971412654
1.320365884	1.316767619	0.971430709
1.320379398	1.316767619	0.971449251
1.320392917	1.316767619	0.971467786
1.315631829	1.316767619	0.971486313
1.315396647	1.316767619	0.971504833
1.31540839	1.316767619	0.971522877
1.320164253	1.316767619	0.971540891
1.315431889	1.316767619	0.971558899
1.315443644	1.316767619	0.971577367
1.315455404	1.316767619	0.97159536
1.331471462	1.316767619	0.971613346
1.331488653	1.316767619	0.971631326
1.315490704	1.316767619	0.971650874
1.315502479	1.316767619	0.971670416
1.311747382	1.316767619	0.971688374
1.311758164	1.316767619	0.971706325
1.311768949	1.316767619	0.971723899
1.314094752	1.316767619	0.971741466
1.314106088	1.316767619	0.971759028
1.314117427	1.316767619	0.971776812
1.31412877	1.316767619	0.97179459

1.313666913	1.316767619	0.971812361
1.327159317	1.316767619	0.971830126
1.329214739	1.317100703	0.971847836
1.329231264	1.317433787	0.971866867
1.329247793	1.317766871	0.971886093
1.326560547	1.318099955	0.971905311
1.32339916	1.318433039	0.971924523
1.330703491	1.318766123	0.97194346
1.323428373	1.319099207	0.971962076
1.323442986	1.319432291	0.971981403
1.323457605	1.319765375	0.972000005
1.323472229	1.320098459	0.9720186
1.323180875	1.320431543	0.972037189
1.330805382	1.320764627	0.972055772
1.330822381	1.321097711	0.972074318
1.330839385	1.321430795	0.972093608
1.324477494	1.321763879	0.97211289
1.316553706	1.322096963	0.972132167
1.325141146	1.322430047	0.972150809
1.325156353	1.322763131	0.972168664
1.325171564	1.323096215	0.972187357
1.326481973	1.323429299	0.972206044
1.340936632	1.323762383	0.972224724
1.332058228	1.324095467	0.972243526
1.340975483	1.324428551	0.972263743
1.335455734	1.324761635	0.972283078
1.344324917	1.325094719	0.972303284
1.344344909	1.325427803	0.972322939
1.344364903	1.325760887	0.972343459
1.336299171	1.326093971	0.972363973
1.337095393	1.326427055	0.97238448
1.340704536	1.326760139	0.972404186
1.329017184	1.327093223	0.972423962
1.339129371	1.327426307	0.972444086
1.329050116	1.327759391	0.972463089
1.339167515	1.328092475	0.972483115
1.339186593	1.328425559	0.972502178
1.339205675	1.328758643	0.972522265
1.323560234	1.329091727	0.972542383
1.339243851	1.329424811	0.972562531
1.350598351	1.329757895	0.972581164
1.332006822	1.330090979	0.972601368
1.332024163	1.330424063	0.972622715
1.326171081	1.330757147	0.972642257

1.334658921	1.331090231	0.972661827
1.325233018	1.331423315	0.972680846
1.315152011	1.331756399	0.972700726
1.321588292	1.332089483	0.972719704
1.322192367	1.332422567	0.972737714
1.322206542	1.332755651	0.972756385
1.320191909	1.333088735	0.972775144
1.331095662	1.333421819	0.97279393
1.331112745	1.333754903	0.972812545
1.325035557	1.334087987	0.972832261
1.321393558	1.334421071	0.972852005
1.325065903	1.334754155	0.972871178
1.325081084	1.335087239	0.97289002
1.32509627	1.335420323	0.972909252
1.334155843	1.335753407	0.972928513
1.334173761	1.336086491	0.972947803
1.330891225	1.336419575	0.972968011
1.332354572	1.336752659	0.972988245
1.33237201	1.337085743	0.973008178
1.344225941	1.337418827	0.973028279
1.348497143	1.337751911	0.973048405
1.348517664	1.338084995	0.973069722
1.348538188	1.338418079	0.97309148
1.348558714	1.338751163	0.973113262
1.337806309	1.339084247	0.973135067
1.332494211	1.339417331	0.973156898
1.346053732	1.339750415	0.973177692
1.346073966	1.340083499	0.973197987
1.356532087	1.340416583	0.973219642
1.361420735	1.340749667	0.973241322
1.350433627	1.341082751	0.973264056
1.361462952	1.341415835	0.973287294
1.361484061	1.341748919	0.973309473
1.350495749	1.342082003	0.973332761
1.361526278	1.342415087	0.973356074
1.361547386	1.342748171	0.973378328
1.352739645	1.343081255	0.973401692
1.354957392	1.343414339	0.973425082
1.342108832	1.343747423	0.973447629
1.339282938	1.344080507	0.973470418
1.342148104	1.344413591	0.973491966
1.339723055	1.344746675	0.97351326
1.339742245	1.345079759	0.973534861
1.337376173	1.345412843	0.973556249

1.337394867	1.345745927	0.973577663
1.337413565	1.346079011	0.97359887
1.337432268	1.346412095	0.973620105
1.337450974	1.346745179	0.973641366
1.341893287	1.347078263	0.973662653
1.341912883	1.347411347	0.973683965
1.337507119	1.347744431	0.973705739
1.340319009	1.348077515	0.97372754
1.347863238	1.348410599	0.973748931
1.365468071	1.348743683	0.973770625
1.365489145	1.349076767	0.973793087
1.348785388	1.349409851	0.973817307
1.361548156	1.349742935	0.973841552
1.361569264	1.350076019	0.973864175
1.359376289	1.350409103	0.97388808
1.359397392	1.350742187	0.973912011
1.375353943	1.351075271	0.97393575
1.362982086	1.351408355	0.973959514
1.350227636	1.351741439	0.973984871
1.350248324	1.352074523	0.974009032
1.348970426	1.352407607	0.974031961
1.363066486	1.352740691	0.974054916
1.349011568	1.353073775	0.974077769
1.349032143	1.353406859	0.974102031
1.349052719	1.353739943	0.97412493
1.360050874	1.354073027	0.974147853
1.344826738	1.354406111	0.974170799
1.344846803	1.354739195	0.97419485
1.360114196	1.355072279	0.974217424
1.344886941	1.355405363	0.974240022
1.359271	1.355738447	0.974264145
1.359292102	1.356071531	0.974286792
1.359313203	1.356404615	0.974310877
1.359334305	1.356737699	0.974334986
1.360240841	1.357070783	0.974359121
1.354957829	1.357403867	0.974383281
1.360283057	1.357736951	0.974407556
1.360304164	1.358070035	0.974431334
1.343391236	1.358403119	0.974455661
1.360346381	1.358736203	0.974480015
1.365236676	1.359069287	0.974502728
1.349402289	1.359402371	0.974527136
1.349422901	1.359735455	0.974552051
1.337413955	1.360068539	0.974575431

1.337432657	1.360401623	0.974598838
1.337451364	1.360734707	0.974621087
1.328625445	1.361067791	0.974643363
1.33748879	1.361400875	0.974665666
1.337507509	1.361733959	0.974687125
1.337526233	1.362067043	0.974709479
1.33754496	1.362400127	0.974731859
1.343233295	1.362733211	0.974754263
1.339169502	1.363066295	0.974776691
1.339188581	1.363399379	0.974799703
1.339207663	1.363732463	0.974822338
1.33922675	1.364065547	0.974844999
1.356723305	1.364398631	0.974867683
1.338865473	1.364731715	0.974890392
1.356765415	1.365064799	0.974914846
1.356786471	1.365397883	0.974937562
1.356807527	1.365730967	0.974962063
1.358154968	1.366064051	0.974986588
1.358618181	1.366397135	0.975011136
1.364837155	1.366730219	0.975035837
1.364858236	1.367063303	0.975060604
1.371065329	1.367396387	0.975086005
1.372413458	1.367729471	0.975111428
1.374653985	1.368062555	0.975137482
1.374675212	1.368395639	0.975163687
1.380120978	1.368728723	0.975190133
1.380142968	1.369061807	0.975216601
1.380164964	1.369394891	0.975243625
1.38690825	1.369727975	0.97527067
1.386932192	1.370061059	0.975297735
1.391091956	1.370394143	0.975325483
1.391117277	1.370727227	0.975353253
1.391142604	1.371060311	0.97538145
1.394962725	1.371393395	0.975409668
1.391193282	1.371726479	0.975437906
1.37850703	1.372059563	0.975466538
1.391243991	1.372392647	0.975494817
1.391269357	1.372725731	0.975521864
1.39129473	1.373058815	0.975550183
1.391854557	1.373391899	0.975578522
1.394047861	1.373724983	0.975606881
1.397948159	1.374058067	0.975635312
1.397974716	1.374391151	0.975663977
1.394674434	1.374724235	0.975693042

1.394700644	1.375057319	0.975722127
1.394726858	1.375390403	0.975750904
1.394753077	1.375723487	0.975779701
1.394779301	1.376056571	0.97580852
1.39480553	1.376389655	0.975837359
1.385910662	1.376722739	0.975866221
1.369907998	1.377055823	0.975895104
1.36992905	1.377388907	0.97592313
1.392186505	1.377721991	0.975949599
1.39221214	1.378055075	0.975976089
1.407998772	1.378388159	0.976004791
1.408022834	1.378721243	0.976033513
1.389119167	1.379054327	0.976063809
1.37182349	1.379387411	0.976094126
1.367868376	1.379720495	0.9761226
1.37186566	1.380053579	0.976149389
1.371886746	1.380386663	0.976175809
1.371907833	1.380719747	0.976202641
1.371928921	1.381052831	0.976229495
1.371950009	1.381385915	0.976256371
1.371971098	1.381718999	0.976283268
1.374654427	1.382052083	0.976310186
1.38581717	1.382385167	0.976337126
1.400308158	1.382718251	0.976364349
1.413959784	1.383051335	0.976392691
1.418854464	1.383384419	0.976422477
1.41887254	1.383717503	0.976453624
1.415304058	1.384050587	0.976485271
1.395436351	1.384383671	0.976516936
1.395462687	1.384716755	0.976548268
1.399937743	1.385049839	0.976577663
1.406461652	1.385382923	0.97660708
1.38171618	1.385716007	0.976636956
1.373575518	1.386049091	0.976667493
1.373596675	1.386382175	0.976695612
1.373617833	1.386715259	0.97672295
1.373638993	1.387048343	0.97675031
1.367469903	1.387381427	0.976777694
1.359968646	1.387714511	0.976805101
1.375483346	1.388047595	0.97683192
1.360010859	1.388380679	0.976858021
1.360031966	1.388713763	0.976885672
1.371550973	1.389046847	0.97691182
1.3873175	1.389379931	0.976937993

1.387341581	1.389713015	0.976965321
1.38736567	1.390046099	0.976994223
1.395357639	1.390379183	0.977023147
1.395383964	1.390712267	0.977052092
1.395410292	1.391045351	0.977081844
1.395436625	1.391378435	0.977111616
1.395462961	1.391711519	0.977141409
1.396043025	1.392044603	0.977171221
1.412325318	1.392377687	0.977201054
1.396095865	1.392710771	0.977230963
1.39612229	1.393043855	0.977262493
1.396148718	1.393376939	0.977292441
1.400626418	1.393710023	0.977322409
1.400652838	1.394043107	0.977352396
1.400679256	1.394376191	0.97738284
1.40070567	1.394709275	0.977413305
1.40073208	1.395042359	0.977443788
1.399091147	1.395375443	0.977474291
1.385771144	1.395708527	0.977504813
1.399144242	1.396041611	0.97753519
1.387824923	1.396374695	0.977564274
1.387849176	1.396707779	0.977594693
1.387873438	1.397040863	0.977624015
1.400362311	1.397373947	0.977653357
1.409374644	1.397707031	0.977682721
1.409398008	1.398040115	0.977713332
1.418528581	1.398373199	0.977744847
1.418546822	1.398706283	0.977776382
1.418565053	1.399039367	0.977808833
1.422222056	1.399372451	0.977841303
1.422238614	1.399705535	0.977873792
1.422255166	1.400038619	0.977906658
1.422271712	1.400371703	0.977939541
1.413311844	1.400704787	0.977972442
1.396202547	1.401037871	0.97800536
1.396228984	1.401370955	0.978037411
1.38520908	1.401704039	0.978067793
1.388751466	1.402037123	0.978098194
1.388776033	1.402370207	0.978127526
1.388800608	1.402703291	0.978157223
1.387293929	1.403036375	0.978186939
1.383867067	1.403369459	0.978216673
1.387342083	1.403702543	0.978246275
1.394781077	1.404035627	0.978275556

1.394807306	1.404368711	0.978305197
1.39483354	1.404701795	0.978335589
1.406363908	1.405034879	0.978366002
1.416561223	1.405367963	0.978396435
1.40641351	1.405701047	0.97842802
1.402698371	1.406034131	0.978460625
1.402724409	1.406367215	0.978492248
1.419729974	1.406700299	0.978523522
1.416657556	1.407033383	0.978554814
1.398374922	1.407366467	0.978587798
1.398401484	1.407699551	0.978620495
1.38867807	1.408032635	0.978651408
1.376337092	1.408365719	0.97868234
1.376358476	1.408698803	0.978712332
1.376379863	1.409031887	0.978741126
1.386244104	1.409364971	0.978769942
1.395227584	1.409698055	0.978798778
1.395253889	1.410031139	0.978828605
1.395280197	1.410364223	0.978859336
1.409304967	1.410697307	0.978890088
1.411247954	1.411030391	0.978920861
1.41127029	1.411363475	0.978953035
1.411292613	1.411696559	0.978985418
1.411314924	1.412029643	0.979017822
1.411337223	1.412362727	0.979050249
1.412283087	1.412695811	0.979082697
1.412304832	1.413028895	0.979115166
1.412326563	1.413361979	0.979147747
1.412348282	1.413695063	0.979180347
1.410024382	1.414028147	0.979212968
1.401706088	1.414361231	0.979245609
1.40173234	1.414694315	0.979278039
1.412435033	1.415027399	0.979309667
1.419818314	1.415360483	0.979341315
1.420927887	1.415693567	0.979374034
1.426968983	1.416026651	0.979407497
1.420962079	1.416359735	0.979441086
1.406191136	1.416692819	0.979475287
1.406216015	1.417025903	0.979508914
1.41917967	1.417358987	0.979541105
1.419941841	1.417692071	0.979573316
1.420325381	1.418025155	0.979606821
1.420342751	1.418358239	0.97964042
1.420360113	1.418691323	0.979674074

1.420377468	1.419024407	0.979707746
1.416154808	1.419357491	0.979741437
1.420412152	1.419690575	0.979775146
1.420429483	1.420023659	0.979808455
1.417811271	1.420356743	0.9798422
1.417040452	1.420356743	0.979875963
1.418236705	1.420356743	0.979909485
1.419018229	1.420356743	0.979942947
1.419036223	1.420356743	0.979976544
1.419054208	1.420356743	0.980010234
1.418693453	1.420356743	0.980043945
1.419090152	1.420356743	0.980077673
1.418729758	1.420356743	0.980111384
1.419126061	1.420356743	0.980145151
1.418766026	1.420356743	0.9801789
1.419536302	1.420356743	0.980212705
1.419180324	1.420356743	0.980246493
1.420671736	1.420356743	0.980280374
1.417287469	1.420356743	0.980314236
1.417700071	1.420356743	0.980348262
1.417718742	1.420356743	0.980381973
1.417737404	1.420356743	0.980415743
1.417756055	1.420356743	0.980449533
1.417774696	1.420356743	0.980483342
1.417793328	1.420356743	0.980517173
1.41781195	1.420356743	0.980551024
1.417437827	1.420356743	0.980584895
1.40804989	1.420356743	0.980618786
1.419018885	1.420356743	0.980652658
1.410071478	1.420356743	0.980685624
1.426289403	1.420356743	0.980719688
1.410117446	1.420356743	0.980752889
1.426319836	1.420356743	0.980787704
1.409189736	1.420356743	0.980820943
1.426350254	1.420356743	0.980855796
1.409236646	1.420356743	0.980888978
1.426380655	1.420356743	0.980923869
1.426395849	1.420356743	0.980957092
1.416869655	1.420356743	0.980992022
1.416077559	1.420356743	0.981026972
1.416097117	1.420356743	0.981061002
1.416116663	1.420356743	0.981094972
1.414884766	1.420356743	0.981128963
1.421472036	1.420356743	0.981162973

1.420412965	1.420356743	0.98119688
1.42150576	1.420356743	0.981231455
1.420447617	1.420356743	0.981265908
1.420464931	1.420356743	0.98130045
1.420482238	1.420356743	0.981334873
1.422961787	1.420356743	0.981369281
1.420516827	1.420356743	0.981403675
1.422994329	1.420356743	0.981438298
1.423010591	1.420356743	0.981472666
1.423026846	1.420356743	0.981507262
1.421657169	1.420356743	0.981541844
1.421673957	1.420356743	0.981576413
1.421690738	1.420356743	0.981610832
1.425739682	1.420356743	0.981645237
1.422074202	1.420356743	0.981679629
1.42209082	1.420356743	0.981714405
1.419959937	1.420356743	0.981748805
1.419977476	1.420356743	0.981783191
1.422831545	1.420356743	0.981817353
1.416136402	1.420356743	0.981851503
1.421824738	1.420356743	0.98188592
1.415764065	1.420356743	0.981919666
1.421858169	1.420356743	0.98195396
1.421874874	1.420356743	0.981987643
1.421891573	1.420356743	0.982021913
1.421908264	1.420356743	0.98205617
1.421924949	1.420356743	0.982090416
1.415882302	1.420356743	0.982124648
1.418293378	1.420356743	0.982158867
1.418311739	1.420356743	0.982192476
1.418330091	1.420356743	0.982226308
1.418348433	1.420356743	0.982260129
1.418366765	1.420356743	0.982293937
1.411628025	1.420356743	0.982327734
1.418017012	1.420356743	0.982361519
1.409283994	1.420356743	0.982394627
1.418054011	1.420356743	0.98242835
1.418072496	1.420356743	0.982461199
1.411738499	1.420356743	0.982494896
1.418494831	1.420356743	0.98252858
1.411782601	1.420356743	0.982561626
1.418531337	1.420356743	0.982595322
1.41658272	1.420356743	0.982628345
1.416601996	1.420356743	0.982662019

1.411870653	1.420356743	0.98269549
1.416640517	1.420356743	0.98272895
1.414149841	1.420356743	0.982761931
1.41825634	1.420356743	0.982795369
1.420153058	1.420356743	0.982828551
1.417905975	1.420356743	0.982862124
1.419820607	1.420356743	0.982895874
1.417943091	1.420356743	0.98292939
1.417961634	1.420356743	0.982963082
1.417980167	1.420356743	0.982996578
1.419890978	1.420356743	0.983030062
1.418017205	1.420356743	0.983063537
1.419926114	1.420356743	0.983097187
1.417664452	1.420356743	0.983130641
1.412632295	1.420356743	0.983164272
1.417701822	1.420356743	0.983197668
1.412675367	1.420356743	0.983230556
1.423528015	1.420356743	0.98326393
1.418146532	1.420356743	0.983296798
1.418164969	1.420356743	0.983330722
1.411848888	1.420356743	0.983364106
1.418201814	1.420356743	0.98339748
1.418220223	1.420356743	0.983430223
1.423959325	1.420356743	0.98346358
1.423975245	1.420356743	0.983496928
1.423991158	1.420356743	0.983530829
1.424007067	1.420356743	0.983564722
1.42402297	1.420356743	0.983598604
1.42502916	1.420356743	0.983632478
1.425044736	1.420356743	0.983666342
1.426351283	1.420356743	0.983700293
1.424418667	1.420356743	0.983734234
1.424434434	1.420356743	0.983768292
1.418422088	1.420356743	0.983802148
1.423463851	1.420356743	0.983835994
1.418458668	1.420356743	0.983869236
1.423496028	1.420356743	0.983902964
1.418495211	1.420356743	0.983936188
1.418894558	1.420356743	0.983969897
1.418912614	1.420356743	0.984003103
1.418930661	1.420356743	0.984036336
1.418948698	1.420356743	0.984069559
1.418966727	1.420356743	0.984102773
1.418984747	1.420356743	0.984135978

1.423624926	1.420356743	0.984169174
1.423640961	1.420356743	0.984202361
1.414192117	1.420356743	0.984235994
1.422652695	1.420356743	0.984269618
1.415507849	1.420356743	0.984302301
1.415527729	1.420356743	0.984335806
1.41250323	1.420356743	0.984368597
1.415567453	1.420356743	0.984401381
1.412546452	1.420356743	0.984433856
1.415607133	1.420356743	0.984466624
1.412589623	1.420356743	0.984499083
1.415646767	1.420356743	0.984531833
1.423141568	1.420356743	0.984564275
1.414418457	1.420356743	0.984597009
1.423512694	1.420356743	0.984630469
1.423528768	1.420356743	0.984663058
1.432599263	1.420356743	0.984696533
1.423560899	1.420356743	0.984729997
1.423576956	1.420356743	0.984764343
1.423593008	1.420356743	0.984797789
1.423609053	1.420356743	0.984831225
1.411452113	1.420356743	0.984864653
1.411474334	1.420356743	0.984898072
1.409588712	1.420356743	0.984930284
1.409611962	1.420356743	0.984962489
1.41753393	1.420356743	0.984994499
1.409658425	1.420356743	0.985026503
1.409681638	1.420356743	0.985059277
1.417590178	1.420356743	0.985091267
1.409728027	1.420356743	0.985123249
1.417627627	1.420356743	0.985156002
1.419926662	1.420356743	0.985187972
1.419944217	1.420356743	0.985220712
1.419961764	1.420356743	0.985253668
1.419979303	1.420356743	0.985286617
1.41267604	1.420356743	0.985319558
1.417346471	1.420356743	0.98535249
1.417365331	1.420356743	0.985384692
1.417384181	1.420356743	0.985417344
1.41740302	1.420356743	0.985449987
1.41742185	1.420356743	0.985482621
1.415409218	1.420356743	0.985515247
1.415429153	1.420356743	0.985547864
1.415449077	1.420356743	0.985580272

1.41546899	1.420356743	0.985612671
1.415488891	1.420356743	0.98564506
1.426625034	1.420356743	0.985677441
1.42943766	1.420356743	0.985709814
1.427912234	1.420356743	0.985743272
1.427927067	1.420356743	0.985776998
1.427941896	1.420356743	0.985810564
1.427956722	1.420356743	0.985844122
1.418423136	1.420356743	0.985877672
1.419201129	1.420356743	0.985911214
1.419219033	1.420356743	0.985943808
1.419236928	1.420356743	0.985976471
1.419254815	1.420356743	0.986009128
1.418514515	1.420356743	0.986041778
1.415746068	1.420356743	0.986074423
1.416584729	1.420356743	0.986106987
1.412762815	1.420356743	0.986139272
1.416623268	1.420356743	0.986171635
1.4215421	1.420356743	0.986203614
1.420484851	1.420356743	0.986235968
1.415449285	1.420356743	0.9862688
1.415469197	1.420356743	0.986301521
1.412439546	1.420356743	0.986333742
1.415508989	1.420356743	0.98636596
1.417158203	1.420356743	0.986397874
1.417177164	1.420356743	0.986430086
1.418751013	1.420356743	0.986462457
1.418769141	1.420356743	0.986494824
1.417233986	1.420356743	0.986527343
1.41304091	1.420356743	0.98655986
1.412612425	1.420356743	0.986592222
1.413083507	1.420356743	0.986624167
1.418859643	1.420356743	0.986656067
1.419628425	1.420356743	0.986688009
1.415727034	1.420356743	0.986720516
1.413611055	1.420356743	0.986753095
1.413632028	1.420356743	0.986785284
1.407981847	1.420356743	0.986817261
1.406986242	1.420356743	0.986849236
1.412353031	1.420356743	0.986880651
1.417062945	1.420356743	0.986911963
1.417081958	1.420356743	0.986943799
1.415884248	1.420356743	0.986976093
1.411051505	1.420356743	0.987008384

1.415923569	1.420356743	0.987040555
1.415943213	1.420356743	0.987072245
1.415962846	1.420356743	0.987104413
1.415982468	1.420356743	0.987136577
1.419892534	1.420356743	0.987168738
1.419910105	1.420356743	0.987200897
1.419927668	1.420356743	0.987233435
1.431636134	1.420356743	0.987265969
1.447997429	1.420356743	0.9872985
1.448013097	1.420731463	0.987332179
1.448028769	1.421106183	0.987367463
1.448044446	1.421480903	0.987402741
1.442383585	1.421855623	0.987438015
1.451465042	1.422230343	0.987473284
1.44425748	1.422605063	0.98750799
1.451498366	1.422979783	0.987543585
1.444287125	1.423354503	0.987578466
1.444301951	1.423729223	0.987614055
1.434751933	1.424103943	0.987648931
1.444331613	1.424478663	0.987683804
1.43477998	1.424853383	0.987717732
1.434794004	1.425228103	0.9877526
1.430651052	1.425602823	0.987786524
1.423045889	1.425977543	0.987820447
1.423062132	1.426352263	0.987853959
1.423078368	1.426726983	0.987886719
1.43070847	1.427101703	0.987919477
1.444139552	1.427476423	0.987952234
1.444154351	1.427851143	0.987985737
1.432246867	1.428225863	0.988020558
1.437566623	1.428600583	0.988055376
1.451067527	1.428975303	0.988089016
1.454302344	1.429350023	0.988123174
1.454319921	1.429724743	0.988158657
1.443009009	1.430099463	0.988194454
1.441806111	1.430474183	0.988230247
1.436473848	1.430848903	0.988264923
1.436487858	1.431223623	0.988299476
1.437090249	1.431598343	0.988333499
1.440660062	1.431973063	0.98836752
1.442181477	1.432347783	0.988401597
1.442195948	1.432722503	0.988436022
1.437440807	1.433097223	0.988470592
1.437454842	1.433471943	0.988505161

1.442239373	1.433846663	0.988539259
1.44992322	1.434221383	0.988573355
1.449939417	1.434596103	0.988607917
1.441074534	1.434970823	0.988643233
1.441088857	1.435345543	0.988678545
1.43518651	1.435720263	0.98871298
1.435200524	1.436094983	0.988747413
1.435214537	1.436469703	0.98878126
1.447050786	1.436844423	0.988815105
1.447066216	1.437219143	0.988848946
1.442384264	1.437593863	0.988883949
1.435270589	1.437968583	0.988918949
1.424578074	1.438343303	0.988953485
1.429336938	1.438718023	0.988987316
1.436488004	1.439092743	0.989020091
1.436502014	1.439467463	0.989053332
1.43445813	1.439842183	0.989087275
1.43000385	1.440216903	0.989121217
1.4300183	1.440591623	0.989154955
1.435088918	1.440966343	0.989188251
1.435102934	1.441341063	0.989221545
1.435116949	1.441715783	0.989255336
1.441046043	1.442090503	0.989289124
1.441060363	1.442465223	0.989322908
1.441074684	1.442839943	0.989357273
1.446345883	1.443214663	0.989391633
1.446361145	1.443589383	0.989425988
1.44899268	1.443964103	0.989460857
1.452814268	1.444338823	0.98949572
1.451416048	1.444713543	0.989530836
1.451432693	1.445088263	0.98956632
1.451449342	1.445462983	0.989601658
1.447748346	1.445837703	0.989636992
1.44776395	1.446212423	0.989672321
1.44616317	1.446587143	0.989707279
1.444287974	1.446961863	0.989742232
1.444302801	1.447336583	0.989777022
1.44186447	1.447711303	0.989811622
1.439779464	1.448086023	0.98984622
1.441893323	1.448460743	0.989880573
1.441907753	1.448835463	0.989914718
1.441922184	1.449210183	0.989949067
1.444391822	1.449584903	0.989983415
1.452355366	1.449959623	0.990017762

1.455672784	1.450334343	0.990052349
1.468880414	1.450709063	0.990087716
1.468903024	1.451083783	0.990123404
1.468925641	1.451458503	0.990160386
1.483288652	1.451833223	0.990197363
1.477461305	1.452207943	0.990234334
1.463959171	1.452582663	0.990272711
1.47751103	1.452957383	0.990310505
1.458945067	1.453332103	0.990346962
1.458164678	1.453706823	0.990384745
1.458183598	1.454081543	0.990420692
1.458202525	1.454456263	0.990456554
1.465874835	1.454830983	0.990492411
1.467272701	1.455205703	0.990528263
1.467294797	1.455580423	0.990564861
1.4663958	1.455955143	0.990601589
1.466417608	1.456329863	0.990638308
1.466439422	1.456704583	0.99067493
1.467849309	1.457079303	0.990711543
1.467871592	1.457454023	0.990748148
1.467893883	1.457828743	0.990784882
1.466526752	1.458203463	0.990821607
1.466548603	1.458578183	0.990858323
1.466570461	1.458952903	0.990894893
1.466592326	1.459327623	0.990931456
1.468005441	1.459702343	0.990968013
1.468027774	1.460077063	0.991004564
1.455781809	1.460451783	0.991041245
1.455799892	1.460826503	0.991077919
1.475981567	1.461201223	0.991113378
1.476006109	1.461575943	0.991148831
1.476547167	1.461950663	0.991186262
1.486186377	1.462325383	0.991223685
1.486212373	1.462700103	0.99126115
1.48623837	1.463074823	0.991299556
1.47664587	1.463449543	0.991337954
1.476670559	1.463824263	0.991376343
1.476695253	1.464198983	0.991413778
1.452661716	1.464573703	0.991451205
1.452678753	1.464948423	0.991488625
1.471711848	1.465323143	0.991523669
1.463376509	1.465697863	0.991558706
1.463397282	1.466072583	0.991595609
1.450986635	1.466447303	0.991631682

1.443831103	1.466822023	0.991667747
1.451019666	1.467196743	0.991702581
1.451036189	1.467571463	0.991736701
1.451052717	1.467946183	0.991771521
1.45106925	1.468320903	0.991806332
1.444215116	1.468695623	0.991841137
1.444229929	1.469070343	0.991875934
1.444244745	1.469445063	0.991910047
1.444259564	1.469819783	0.991944153
1.43264464	1.470194503	0.991978251
1.434431377	1.470569223	0.992012344
1.434150714	1.470943943	0.992045283
1.43416476	1.471318663	0.99207839
1.448835062	1.471693383	0.992111459
1.457508423	1.472068103	0.99214452
1.467828078	1.472442823	0.992179016
1.467850354	1.472817543	0.992214355
1.457564506	1.473192263	0.9922507
1.466049117	1.473566983	0.992287037
1.466070808	1.473941703	0.99232235
1.490298494	1.474316423	0.992358486
1.503536409	1.474691143	0.992394614
1.503560143	1.475065863	0.992433117
1.490376606	1.475440583	0.992472912
1.493668583	1.475815303	0.992512697
1.481189126	1.476190023	0.992551171
1.481214634	1.476564743	0.992589955
1.481240146	1.476939463	0.992627499
1.465811218	1.477314183	0.992665033
1.46583283	1.477688903	0.992702556
1.455118311	1.478063623	0.992738549
1.448455697	1.478438343	0.992774533
1.448471486	1.478813063	0.992809452
1.439172315	1.479187783	0.992843706
1.439186448	1.479562503	0.992877952
1.439200583	1.479937223	0.992911273
1.439214718	1.480311943	0.992944588
1.446271213	1.480686663	0.992977898
1.446286458	1.481061383	0.993011202
1.452356954	1.481436103	0.993045194
1.452373893	1.481810823	0.993079178
1.452390838	1.482185543	0.993113752
1.458831815	1.482560263	0.993148319
1.469402933	1.482934983	0.993182878

1.480603998	1.483309703	0.993218061
1.48062942	1.483684423	0.993254274
1.469471269	1.484059143	0.993291579
1.458927674	1.484433863	0.993328873
1.453589228	1.484808583	0.993365057
1.453606568	1.485183303	0.993400191
1.466746841	1.485558023	0.993434789
1.473511909	1.485932743	0.993469378
1.48187842	1.486307463	0.993505251
1.481904022	1.486682183	0.993541779
1.477686914	1.487056903	0.99357912
1.477190763	1.487431623	0.993616452
1.477215567	1.487806343	0.993653358
1.477240377	1.488181063	0.993690204
1.477265192	1.488555783	0.993727038
1.477290012	1.488930503	0.993763861
1.477314837	1.489305223	0.993800673
1.475787089	1.489679943	0.993837475
1.471292411	1.490054663	0.993874266
1.471315745	1.490429383	0.993910893
1.461318369	1.490804103	0.993947063
1.461338415	1.491178823	0.993983221
1.471385785	1.491553543	0.994018381
1.484969266	1.491928263	0.994053529
1.498531494	1.492302983	0.994089653
1.510267223	1.492677703	0.994127101
1.50014755	1.493052423	0.994165869
1.508509541	1.493427143	0.994205777
1.508531388	1.493801863	0.994244671
1.500221613	1.494176583	0.994284372
1.508575053	1.494551303	0.994324059
1.508596871	1.494926023	0.994362912
1.52025657	1.495300743	0.994402572
1.520271587	1.495675463	0.994442219
1.520286592	1.496050183	0.994482997
1.520301587	1.496424903	0.994523759
1.527045759	1.496799623	0.994564506
1.527054566	1.497174343	0.994605238
1.530182835	1.497549063	0.994646617
1.530183169	1.497923783	0.994687982
1.527080921	1.498298503	0.994729637
1.520702078	1.498673223	0.994771273
1.523291579	1.499047943	0.994812585
1.523304174	1.499422663	0.99485325

1.512379313	1.499797383	0.99489415
1.502149896	1.500172103	0.994935033
1.502174054	1.500546823	0.994974823
1.510289032	1.500921543	0.995013587
1.502222349	1.501296263	0.995052336
1.484013179	1.501670983	0.995091865
1.484039019	1.502045703	0.995130581
1.502294739	1.502420423	0.995167487
1.484090706	1.502795143	0.995204381
1.492843919	1.503169863	0.995243056
1.493412723	1.503544583	0.995279922
1.517255847	1.503919303	0.995317634
1.497754202	1.504294023	0.995355385
1.505892982	1.504668743	0.995395468
1.528916332	1.505043463	0.995433612
1.528922262	1.505418183	0.995472542
1.528928179	1.505792903	0.995513722
1.521294848	1.506167623	0.995554884
1.528939977	1.506542343	0.995596027
1.521323295	1.506917063	0.9956364
1.521337502	1.507291783	0.995677507
1.520746628	1.507666503	0.995717843
1.520761278	1.508041223	0.995758161
1.516738408	1.508415943	0.995798401
1.50848871	1.508790663	0.995838621
1.51677327	1.509165383	0.995878426
1.508532412	1.509540103	0.995917397
1.508554249	1.509914823	0.995957163
1.501787825	1.510289543	0.995996098
1.496056171	1.510664263	0.996035016
1.488478828	1.511038983	0.996073247
1.488504889	1.511413703	0.996110895
1.48853095	1.511788423	0.996147778
1.492921855	1.512163143	0.996184646
1.496717819	1.512537863	0.9962215
1.489156156	1.512912583	0.996258769
1.477863437	1.513287303	0.996296395
1.488114266	1.513662023	0.996333259
1.492507683	1.514036743	0.996368993
1.506960104	1.514411463	0.996405719
1.521910329	1.514786183	0.996442859
1.525488916	1.515160903	0.996481402
1.521937805	1.515535623	0.996521397
1.512816578	1.515910343	0.996561723

1.512836321	1.516285063	0.996601677
1.521978936	1.516659783	0.99664071
1.521992624	1.517034503	0.996679724
1.523380104	1.517409223	0.996719616
1.51957091	1.517783943	0.996759488
1.517187684	1.518158663	0.996799474
1.51720483	1.518533383	0.996839064
1.509973016	1.518908103	0.996878398
1.508641728	1.519282823	0.996917712
1.517256204	1.519657543	0.996956293
1.517273306	1.520032263	0.996994725
1.517290397	1.520406983	0.997033985
1.517307477	1.520781703	0.997073229
1.505457365	1.521156423	0.997112455
1.509676203	1.521531143	0.997151665
1.502537332	1.521905863	0.997189689
1.502561376	1.522280583	0.997228112
1.510184932	1.522655303	0.997265812
1.524323283	1.523030023	0.997303494
1.524334956	1.523404743	0.997341906
1.522517414	1.523779463	0.997381688
1.511143209	1.524154183	0.997421448
1.511163822	1.524528903	0.997461006
1.511184425	1.524903623	0.997499421
1.516050929	1.525278343	0.997537816
1.524158842	1.525653063	0.997576189
1.516825841	1.526027783	0.997615018
1.524182481	1.526402503	0.997654621
1.51686059	1.526777223	0.997693479
1.516877948	1.527151943	0.99773304
1.512180316	1.527526663	0.997771856
1.516912631	1.527901383	0.997810651
1.527046586	1.528276103	0.997848961
1.530185913	1.528650823	0.997887715
1.528998238	1.529025543	0.997927444
1.528989916	1.529400263	0.99796746
1.528981559	1.529774983	0.998007335
1.529561477	1.530149703	0.998047186
1.529555776	1.530524423	0.998087014
1.530185327	1.530899143	0.998126876
1.530185161	1.531273863	0.998166715
1.528134833	1.531648583	0.998206593
1.528142142	1.532023303	0.998246447
1.529516146	1.532398023	0.998286074

1.528306844	1.532772743	0.998325678
1.521717199	1.533147463	0.998365392
1.526795797	1.533522183	0.998404962
1.525351485	1.533896903	0.998443858
1.52393268	1.534271623	0.998483231
1.528192986	1.534646343	0.998522438
1.528200204	1.535021063	0.998561481
1.529933169	1.535395783	0.998600922
1.529936039	1.535770503	0.998640339
1.528221789	1.536145223	0.998679905
1.528228961	1.536519943	0.998719448
1.528236122	1.536894663	0.998758797
1.528390688	1.537269383	0.998798123
1.530005248	1.537644103	0.998837426
1.530007702	1.538018823	0.99887672
1.530010141	1.538393543	0.998916148
1.530012563	1.538768263	0.998955551
1.53001497	1.539142983	0.998994928
1.526356216	1.539517703	0.99903428
1.52748989	1.539892423	0.999073606
1.527498126	1.540267143	0.999112545
1.527506351	1.540641863	0.999151571
1.529219185	1.541016583	0.999190572
1.522020751	1.541391303	0.999229549
1.529116901	1.541766023	0.999268669
1.521759364	1.542140743	0.999307056
1.521773227	1.542515463	0.999346118
1.52178708	1.542890183	0.999384429
1.529138821	1.543264903	0.999422717
1.529885676	1.543639623	0.999460981
1.527407013	1.544014343	0.999499944
1.530182523	1.544389063	0.999538956
1.527763411	1.544763783	0.9995777
1.529816718	1.545138503	0.999616692
1.527779119	1.545513223	0.99965542
1.529823536	1.545887943	0.999694325
1.529826923	1.546262663	0.999733004
1.529830296	1.546637383	0.999771858
1.527810399	1.547012103	0.999810687
1.527818192	1.547386823	0.999849491
1.527825973	1.547761543	0.99988807
